# A new quinoxaline-containing peptide induces apoptosis in cancer cells by autophagy modulation†
†Electronic supplementary information (ESI) available: Supplemental figures, experimental details and characterization data. See DOI: 10.1039/c5sc00125k



**DOI:** 10.1039/c5sc00125k

**Published:** 2015-05-20

**Authors:** Rubí Zamudio-Vázquez, Saška Ivanova, Miguel Moreno, Maria Isabel Hernandez-Alvarez, Ernest Giralt, Axel Bidon-Chanal, Antonio Zorzano, Fernando Albericio, Judit Tulla-Puche

**Affiliations:** a Institute for Research in Biomedicine , Baldiri Reixac 10 , 08028 Barcelona , Spain . Email: jtulla6@gmail.com ; Email: albericio@irbbarcelona.org ; Email: antonio.zorzano@irbbarcelona.org ; Fax: +34 934037126 ; Tel: +34 934037127; b CIBER-BBN , Networking Centre on Bioengineering , Biomaterials and Nanomedicine , Baldiri Reixac 10 , 08028 Barcelona , Spain; c Department of Biochemistry and Molecular Biology , Faculty of Biology , University of Barcelona , Barcelona , Spain; d CIBER de Diabetes y Enfermedades Metabólicas Asociadas (CIBERDEM) , Instituto de Salud Carlos III , Barcelona , Spain; e Department of Organic Chemistry , Faculty of Chemistry , University of Barcelona , Barcelona , Spain; f Department of Physical Chemistry and Institute of Biomedicine (IBUB) , Faculty of Pharmacy , University of Barcelona , Santa Coloma de Gramenet , Spain; g School of Chemistry , Yachay Tech , Yachay City of Knowledge , Urcuquí 100119 , Ecuador

## Abstract

The synthesis of a new small library of quinoxaline-containing peptides is described. After cytotoxic evaluation in four human cancer cell lines, as well as detailed biological studies, it was found that the most active compound, RZ2, promotes the formation of acidic compartments, where it accumulates, blocking the progression of autophagy. Further disruption of the mitochondrial membrane potential and an increase in mitochondrial ROS was observed, causing cells to undergo apoptosis. Given its cytotoxic activity and protease-resistant features, RZ2 could be a potential drug candidate for cancer treatment and provide a basis for future research into the crosstalk between autophagy and apoptosis and its relevance in cancer therapy.

## Abstract 2

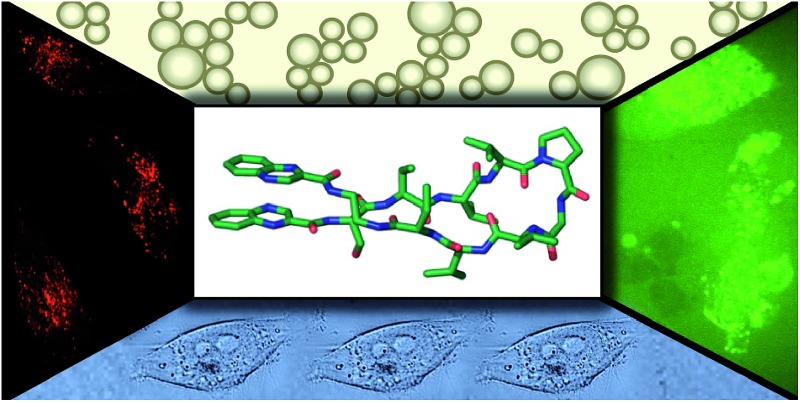
The most cytotoxic compound from a library of quinoxaline-containing peptides is endocyted into HeLa cells, accumulates in acidic compartments, and blocks autophagy by altering lysosomal function, leading to apoptosis activation.

## Introduction

Cancer is a global health concern, accounting for 13% of deaths worldwide.^1^ Cancer cells acquire biological capacities during tumor development; *i.e.* continuous proliferation; avoidance of growth suppressors; resistance to cell death, thus allowing replicative immortality; promotion of angiogenesis, invasion and metastasis; reprogramming of energy metabolism and evasion of immune recognition.^2^ Metabolic and therapeutic stresses activate signal transduction pathways that regulate adaptive responses and suicide signals, and the sum of these determines cell death or cell survival.^3^ Based on biochemical hallmarks and morphology of a dying cell there are three well defined forms of cell death: apoptosis, autophagy, and necrosis.^4^

Apoptosis is an inherently controlled and continual phenomenon throughout the life of an organism. It plays a vital role in development, physiology, and homeostasis under both physiological and pathological conditions and can be initiated or inhibited by a variety of environmental stimuli.^5^ Apoptosis is triggered by the extrinsic (through cell death receptors) or intrinsic pathway (caused by cell damage that cannot be repaired), and it is recognized by widespread proteolysis by caspases, nucleosomal fragmentation by endonucleases, and cell surface tagging for phagocyte engulfment.^6^ Although extrinsic and intrinsic apoptotic pathways act independently at the beginning, they converge on mitochondria in order to activate effector caspases.^7^ One of the hallmarks of cancer cells is their ability to evade apoptosis. This can occur by an increase in antiapoptotic molecules or by a decrease or defective function in proapoptotic proteins.^8^ Given that the number of genetic and epigenetic defects that can suppress apoptosis in most cancers is expanding, understanding the significance of the alternative stress fates, autophagy and necrosis, is becoming increasingly important.^3^

Autophagy (self-eating) is a tightly regulated catabolic process where cytoplasmic components are sequestered in double-membraned autophagosomes that fuse with lysosomes for breakdown by resident hydrolases.^9^ It is stimulated by nutrient or growth factor deprivation, hypoxia, reactive oxygen species (ROS), DNA damage, protein aggregates, damaged organelles, or intracellular pathogens.^10^ Autophagy is generally considered as a protective response preventing the apoptotic pathway to be activated.^11,12^ However, if the intensity of the stress is too high or the duration is prolonged, autophagy cannot relieve it and apoptosis is activated. These two processes cross-regulate each other, mostly in an inhibitory manner.^13^ In cancer, autophagy plays a dual role. In the non-transformed cells it has a tumour suppressive function, eliminating damaged and dysfunctional organelles, thus preventing tumorigenesis.^13^ On the other hand, transformed/cancerous cells depend on autophagy in various stress conditions, including starvation or cancer therapy.^13^ Currently, different autophagic inhibitors are in different stages of clinical trials.^13^

**Fig. 1:**
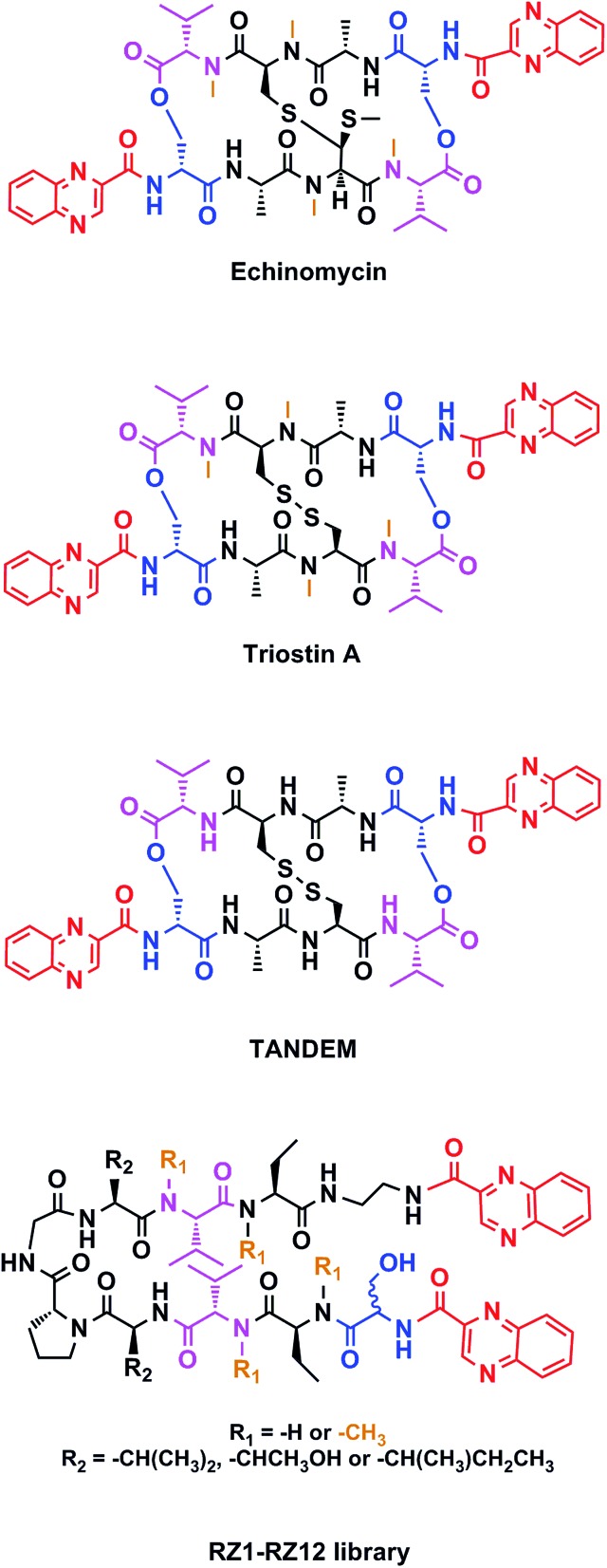
Structures of echinomycin, triostin A, TANDEM and general structure for the novel RZ1–RZ12 library. Common features between all chemical structures are shown in color. Quinoxaline moieties are highlighted in red. Serine residues are shown in blue and valines in pink. Orange depicts *N*-methylations.

Chemotherapy is the most common treatment for cancer to date, and many of the compounds that have been clinically approved or are in clinical trials are natural products or synthetic analogs that are able to interact with several specific targets within the cancer cell. However, nature creates extremely complex molecules that can sometimes be obtained only in small amounts from natural sources. Therefore, although a challenge, the chemical synthesis of such molecules is mandatory. Our research group has focused on the solid-phase synthesis of natural products such as triostin A^14^ and thiocoraline,^15^ since both display high antitumor activity and a fascinating synthetic architecture. Triostin A is a member of the quinoxaline family of bisintercalator antibiotics,^16^ which also encompasses its synthetic demethylated analog TANDEM^17^ and the natural compound echinomycin.^18^ These three molecules are cyclic depsipeptides comprising eight amino acids that have two quinoxaline rings attached to them (Fig. 1).

Compounds containing the quinoxaline core found in many natural and biologically active molecules are validated hits from high-throughput screenings, clinical candidates, and commercial drugs.^19^ Quinoxalines are moieties of great interest in medicinal chemistry due to their capacity to interact with several biological targets. They show various activities including antiviral,^20^ antimicrobial,^21^ antiparasitic,^22^ and antineoplastic action.^23^

Continuing with our research efforts into the design and synthesis of novel anticancer agents and inspired by the aforementioned bisintercalator peptidic scaffolds, we envisaged a small library of peptides with two quinoxaline units covalently attached to both ends. Our analogs have a β-hairpin motif in which two antiparallel strands are connected by a two-residue loop (d-Pro–Gly).^24^ In addition, interstrand side chain-side chain interactions stabilize their β-sheet secondary structure.^25^ We evaluated these newly synthesized compounds *in vitro* for antitumor activity against four human cancer cell lines and explored the cell death mechanism.

## Results and discussion

### Design and synthesis of the RZ1–RZ12 library

**Fig. 2:**
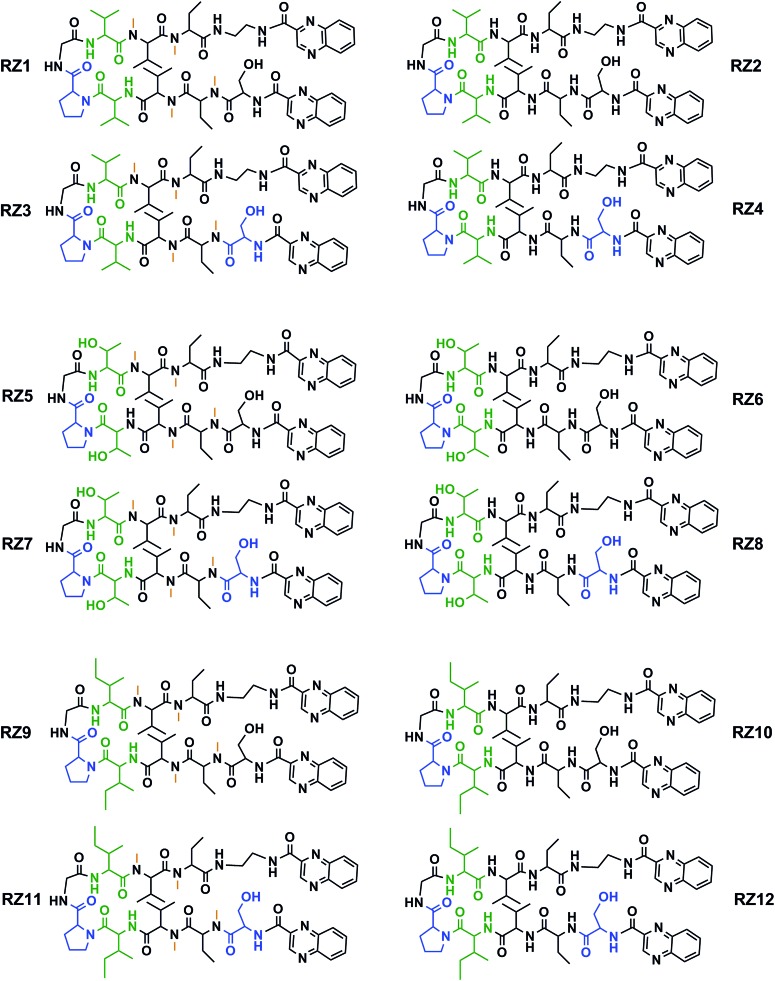
Chemical structures of the RZ1–RZ12 library. For identification purposes d-amino acids are shown in blue and *N*-methylations in orange. The β-branched amino acids (valines, threonines or isoleucines) next to the β-loop that define the four-residue β-turn are shown in green. See Fig. S1 and Table S1† for HPLC chromatograms and characterization of these compounds.

This new library of compounds shows the following characteristics: (1) a two-residue short loop at d-Pro–Gly; (2) the presence of β-branched amino acids next to the β-loop to define a four residue β-turn; (3) two valines, which are also present in echinomycin, triostin A and TANDEM; (4) two α-aminobutyric acid (Abu) residues mimicking the cysteine residues present in the aforementioned natural compounds; (5) a serine residue that holds a d configuration in half of the new compounds and that is changed to l-serine in the other half; (6) an ethylenediamine (EDA) at the *C*-terminus; (7) two quinoxaline moieties linked to the *N*-terminus and the modified *C*-terminus of the formed β-hairpin, and (8) four *N*-methylated amino acids, as in the chemical structure of echinomycin and triostin. The corresponding demethylated analogs were also synthesized, as TANDEM is for triostin A. The total set of twelve compounds is illustrated in Fig. 2.

**Fig. 3:**
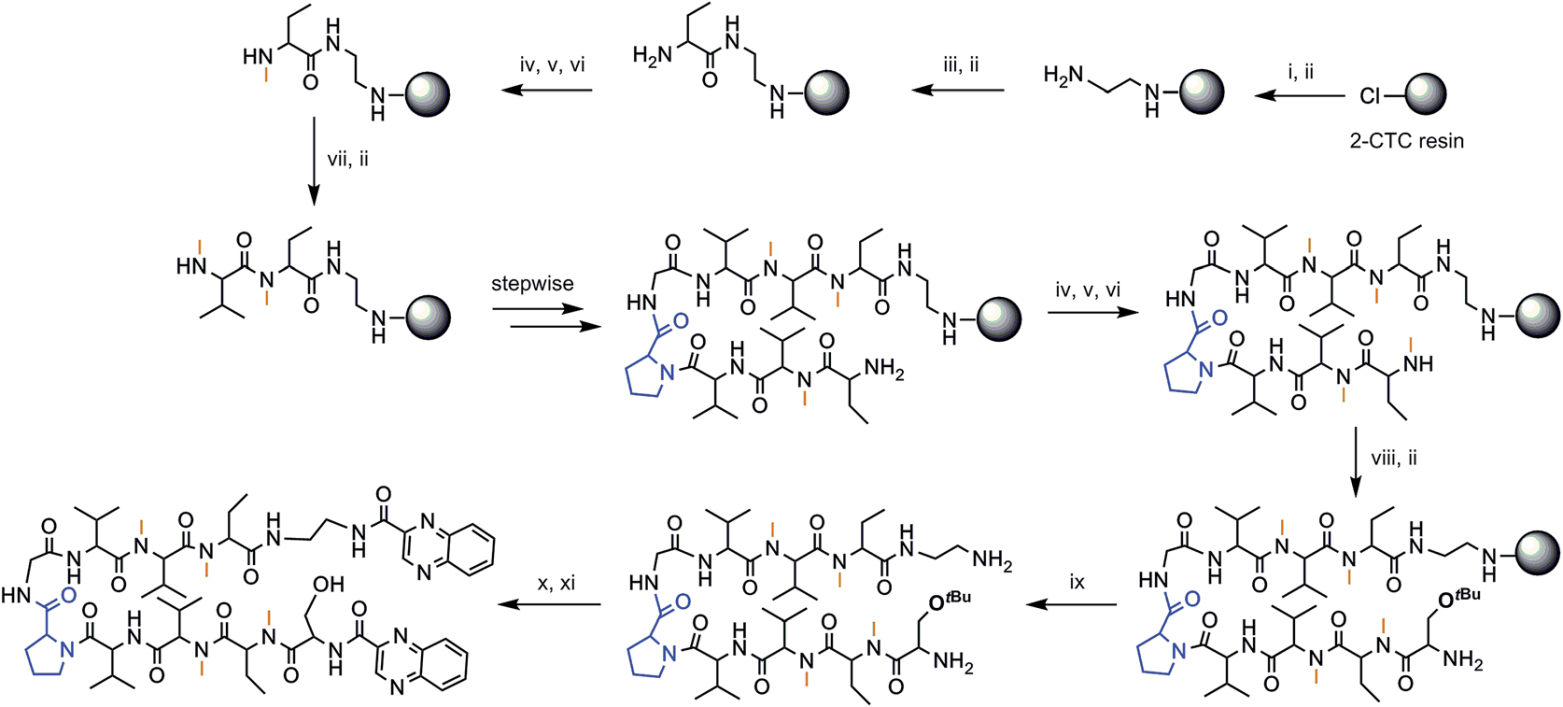
Solid-phase synthesis of compound RZ1. (i) Fmoc–EDA·HCl, DIEA, CH_2_Cl_2_, 45 min; (ii) piperidine–DMF (1 : 4) (2 × 1 min, 2 × 5 min); (iii) Fmoc–Abu-OH, COMU, OxymaPure, DIEA and DMF, 1.5 h; (iv) *o*-NBS-Cl, 2,4,6-collidine and CH_2_Cl_2_, 1.5 h; (v) PPh_3_, MeOH, DIAD and THF, 20 min; (vi) OH–CH_2_–CH_2_–SH, DBU and DMF (3 × 15 min); (vii) Fmoc–*N*Me–Val-OH, COMU, OxymaPure, DIEA and DMF, 1.5 h; (viii) Fmoc–Ser(*t*Bu)-OH, COMU, OxymaPure, DIEA and DMF, 1.5 h; (ix) TFA–CH_2_Cl_2_ (1 : 4) (10 × 30 s); (x) PyBOP, HOAt, 2-quinoxalinecarboxylic acid, DMF and CH_2_Cl_2_, pH 8; (xi) TFA–H_2_O (95 : 5), 2 h.

The chosen approach to synthesize the peptidic scaffolds was a stepwise solid-phase peptide synthesis (SPPS) with the incorporation of the 2-quinoxalinecarboxylic acids (Qxn) and side-chain deprotection as final stages carried out in solution. All SPPSs were done following a 9-fluorenylmethoxycarbonyl/*tert*-butyl (Fmoc/*t*Bu) strategy and *in situ N*-methylations were carried out on the solid support under Mitsunobu conditions when necessary (see Fig. 3 for a representative scheme of the synthetic pathway followed).

The synthesis of peptides with consecutive *N*-methyl amino acids is a challenge because couplings between them are difficult to achieve and they can undergo internal diketopiperazine (DKP) formation.^26^ We therefore opted to use 2-chlorotrityl chloride (2-CTC) resin as solid support since it tolerates *in situ N*-methylations under Mitsunobu conditions when performed after the first residue, it minimizes DKP formation,^27^ and it allows the release of the peptide under mild acidic conditions, thus permitting us to keep the *tert*-butyl side-chain protection of the threonine and serine residues for further reactions performed in solution.

Not only does our peptide library have consecutive *N*-methyl amino acids, but also consecutive β-branched amino acids, the couplings of which are complex as well. In this regard, coupling reagents were chosen on the basis of an efficient combination of the third generation uronium salt COMU^28^ and OxymaPure® in the presence of diisopropylethylamine (DIEA), in order to minimize the risk of racemization. For full completion of the reaction, two or even three couplings at 50 °C were sometimes required.

After cleavage from the resin, the Qxn moieties were introduced using PyBOP/HOAt/DIEA at pH 8, since this phosphonium salt offers the possibility to run long coupling reactions without the formation of undesired byproducts. Finally, global deprotection was carried out with trifluoroacetic acid (TFA)–H_2_O (95 : 5) in 2 h. The crude products were purified by semi-preparative reversed-phase high-performance liquid chromatography (RP-HPLC) to furnish purities over 90%, as shown by analytical HPLC (see Fig. S1 and Table S1†).

### *In vitro* cytotoxic activity of the RZ1–RZ12 library

The twelve synthesized compounds were evaluated *in vitro* using the following four human cancer cell lines: cervical adenocarcinoma (HeLa), lung carcinoma (A-549), breast adenocarcinoma (SK-BR-3) and colon adenocarcinoma (HT-29). Cells were treated for 24 h with various concentrations of the compounds, and their cytotoxic activity was then assessed using the [3-(4,5-dimethylthiazol-2-yl)-2,5-diphenyltetrazolium] bromide (MTT) assay.^29^ Analysis of the dose-response data obtained yielded IC_50_ (50% inhibitory concentration) values, which are detailed in Table 1.

**Table 1:** Cytotoxic activity of the RZ1–RZ12 library against several human cancer cell lines^*a*^

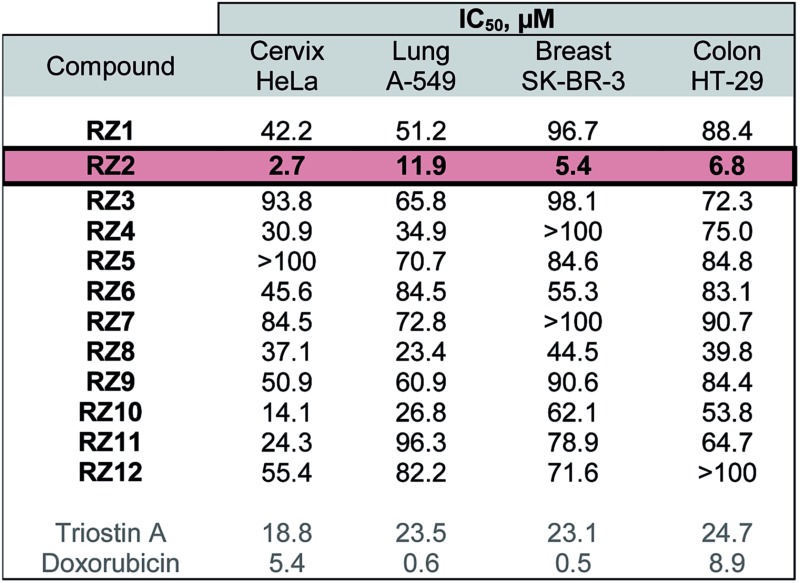

^*a*^Triostin A and doxorubicin were used as positive controls. The most active compound, RZ2, is highlighted.

The most active compound was RZ2, whose peptidic scaffold bears four valines, an l-serine attached to one of the quinoxalines, and no *N*-methylations. It is worth highlighting this last feature since the synthesis of this compound is much easier and more scalable than, for example, its *N*-methylated analog RZ1, thus making it possible to obtain significant amounts of this compound for further biological studies.

### RZ2 is not a DNA-binder

Footprinting is one of the most powerful techniques for the study of ligand–DNA interactions^30^ and it is currently being used for assessing the sequence selectivity of novel ligands. Given that our compounds are simplified analogs of natural bisintercalators, DNase I footprinting experiments were performed for identifying the sequence-specific interaction of our molecules with DNA. Clear footprints were obtained with the positive controls (echinomycin and triostin A), whereas none of the synthesized compounds produced any footprint (Fig. S2†). Additionally, negative results obtained in band shift experiments and circular dichroism spectroscopy (data not shown), corroborate that our analogs do not bisintercalate into the DNA.

### RZ2 adopts a β-hairpin fold that prevents DNA bisintercalation

The propensity of RZ2 to fold as a β-hairpin was examined using two strategies based on Molecular Dynamics (MD) simulations. First, five independent MD simulations, each 1.1 μs long and starting from a completely extended configuration of the peptide, were run and the most populated conformations were obtained by clustering analysis of the snapshots collected along the trajectories. Second, a Replica Exchange Molecular Dynamics Simulation (REMD) was run to better characterize the most stable conformers in terms of secondary structure, as this method allows a better conformational sampling in the study of peptide folding.^31^

The analysis of the independent MD simulations showed that in two cases the most populated conformers (with populations of 89% and 84%) resembled a type II β-hairpin, in which the β-turn involved the central residues d-Pro and Gly, while residues Val–Val–Abu–Ser–Qxn at one side of the turn and Val–Val–Abu–EDA–Qxn at the other side formed the β-sheet. Stable hydrogen bonds were found between the N–H and C=O amide groups of the Val residues and between the Abu subunits at both sides of the β-sheet. In the other three simulations, the β-turn was formed by the residues d-Pro and Val, which prevented the complete formation of the β-sheet within the simulated time.

**Fig. 4:**
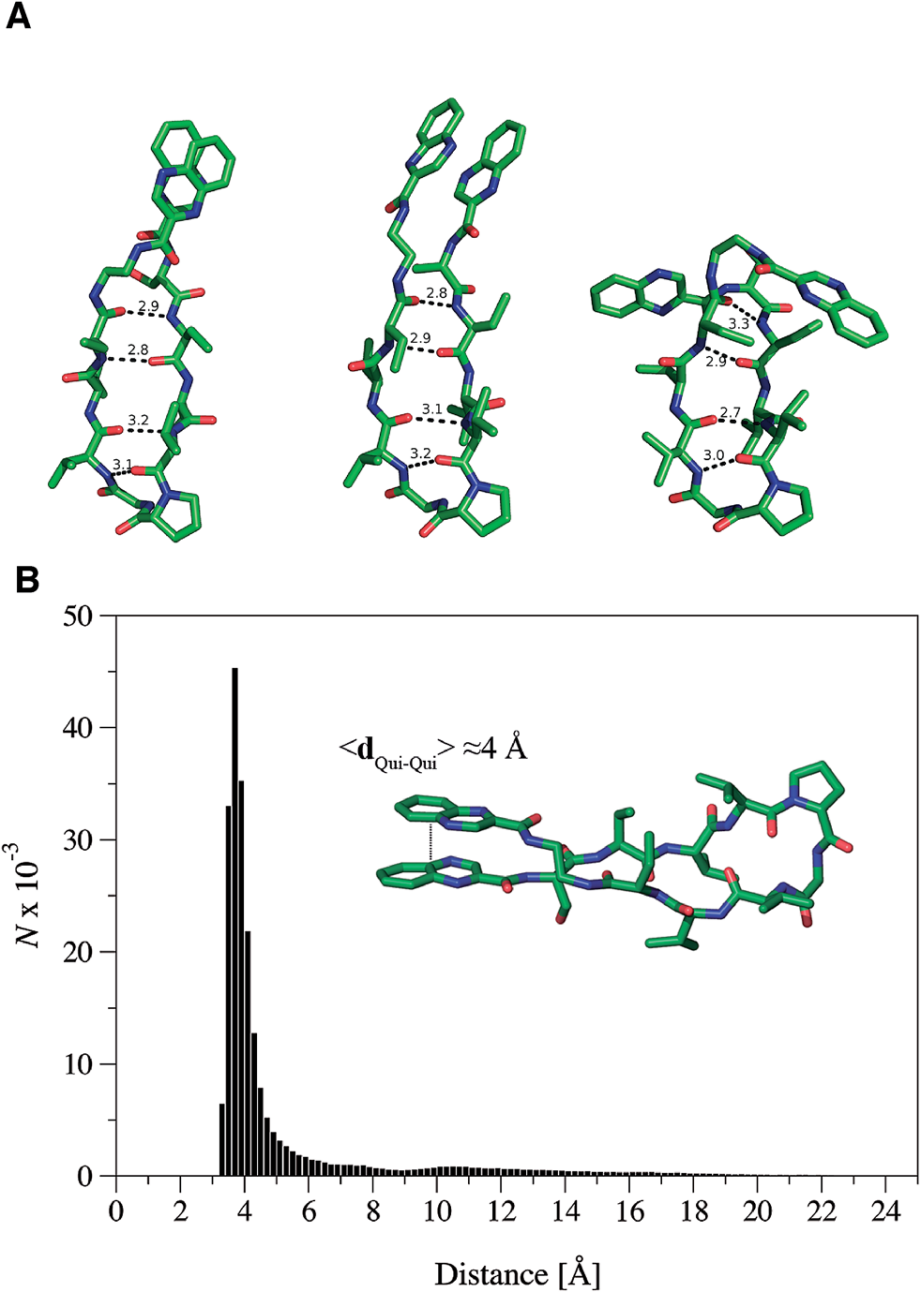
RZ2 adopts a β-hairpin fold. (A) Characteristic structures of the most populated β-hairpin conformers from the MD simulations showing the mean hydrogen bond distances between the N–H···O=C groups forming the β-sheet. The main difference between the three structures is the relative position of the quinoxaline rings as this is the most variable part once the β-hairpin is formed. For clarity, hydrogen atoms were omitted. (B) Histogram representation of the distance between the quinoxaline ring centers, d_Qui–Qui_, measured every 5 ps for a representative molecular dynamics simulation of the peptide starting from the unfolded state. The mean distance measured for the folded state is around 4 Å.

In order to further explore the conformational space of the peptides, REMD simulations were run for the RZ2 peptide. The clustering analysis of the six trajectories ranging from 298.93 K to 310.41 K showed that the most populated conformers had the correct d-Pro–Gly β-turn. Moreover, although the peptide sampled multiple conformations in the 298.93 K trajectory corresponding to the unfolded, partially folded and folded structures, the most populated state was the β-hairpin conformation (Fig. 4A). The largest structural diversity in the β-hairpin corresponded to the relative location of the quinoxaline rings, which adopted different geometrical arrangements, even though a preference toward stacked structures was observed in the six trajectories.

Formation of the β-sheet stabilizes the π–π stacking interaction between the quinoxaline rings and *vice versa*, thus resulting in an average distance between the ring center of the quinoxaline moieties of ∼4 Å (Fig. 4B). Inspection of the crystallographic structures of known bisintercalators bound to DNA, like echinomycin, triostin A or quinomycin (PDB ID ; 1PFE,^32^; 1VS2 ^33^ and ; 193D^34^ respectively), show that the distance between the quinoxaline moieties is ∼10.5 Å, more than 6 Å larger than that found for the RZ2 peptide structure in solution. Noteworthy, the geometrical arrangement of these bisintercalators in the cyclopeptide prevents the formation of an intramolecular π-stacking between the quinoxaline rings. In contrast, it would have been necessary to break the π–π stacking interaction in RZ2, which is over stabilised by the β-sheet structure of the peptide, in order to achieve a reliable distance between the rings for intercalation into the DNA. Thus, the precise nature of the peptide scaffold likely limits the ability of RZ2 to act as DNA bisintercalator.

To get a better understanding on the structural differences that modulate the activity of the compounds, four independent molecular dynamic simulations were run for the peptides RZ1, RZ6 and RZ10; one starting from a completely unfolded conformation following the same protocol used for peptide RZ2 and the other three taking as starting point the final folded conformation of three different replicas run for RZ2 in which the corresponding residues were mutated. Compound RZ1 was chosen to explore the impact of *N*-methylation in different parts of the sequence onto the secondary structure of the peptide, while RZ6 and RZ10 were selected to explore the influence of changing the residues adjacent to the hairpin onto its formation. The results obtained clearly show that *N*-methylation disrupts the β-sheet disregarding the position in which it is introduced. Thus peptides having methylated residues exhibit low population of the β-sheet folded conformations during the molecular dynamic simulations (Fig. S3A†). The same trend is observed when the valines adjacent to the hairpin are mutated to threonines. The presence of a hydroxyl group at the side chain of the residue has a negative impact onto the formation of the β-sheet as it interacts through hydrogen bonds with other amino acids of the peptide preventing the correct formation of the fold (Fig. S3B†). On the other hand, when the aforementioned valines are mutated to isoleucines, the trend is completely reversed and the peptide shows a remarkable increase in the population of the β-sheet conformer (Fig. S3C†) with a nearly inexistent percentage of unfolded or partially unfolded conformations. Overall, it seems that the presence of the β-sheet favours the activity of the peptide, but when the β-sheet conformer is stabilized in excess and its population increases significantly over that observed for compound RZ2, its activity diminishes. Peptide RZ2 presents this subtle equilibrium between the unfolded and β-sheet folded conformations and this might be the reason why it is the most active one.

### RZ2 is stable and hemocompatible under physiological conditions

*N*-methylations make peptides more stable towards proteolytic cleavage and enzymatic degradation.^35^ Thus, we were concerned by the fact that RZ2 has only one d-amino acid and no *N*-methylations. Since the *in vivo* stability of peptides in blood is well modeled by their *in vitro* stability in serum or plasma, where the predominant degradation mechanism is exopeptidase-catalyzed cleavage,^36^ we examined the stability of RZ2 in human serum over 48 h. Furthermore, we studied the stability of this compound in the presence of two overexpressed tumor proteases, metalloproteinase 2 (MMP-2) and cathepsin B, which could cleave the peptidic scaffold, thus inactivating the compound. MMP-2 is located on the surface of microvascular endothelial cells in several cancers and tumors,^37^ and cathepsin B is thought to be overexpressed at the extra and intra-cellular levels, being located mainly in the endo/lysosomal compartments.^38^ Overall results in Fig. S4† display the course of degradation up to 48 h. A little amount of degradation is observed at 24 h, with the maximum degradation (30%) in presence of serum at 48 h. It is worth highlighting that the half-life of the compound in presence of serum proteases, cathepsin B or MMP-2 is more than 48 h. These findings reveal a major edge of our antineoplastic molecule over other peptidic compounds in that they are easily degraded under physiological conditions in short times. These data from stability assays indicate that the peptide could offer advantages in terms of proteolytic stability *in vivo*. Furthermore, to predict the biocompatibility of our hit compound with biological environments, we characterized its capacity to perturb the bilayer of liposomes, which mimics cell surfaces, and to cause red blood cell hemolysis. In both assays, the effects of RZ2 were compared with those of melittin, the toxicity of which is well known. Dye leakage results (Fig. S4D†) show the incapacity of our compound to permeabilize the bilayer, in contrast to the high effect of melittin. Regarding the hemolysis assay (Fig. S4E†), the results show that, at concentrations up to 100 μM, RZ2 was not hemolytic *ex vivo*. Good correlation was observed between both experiments, thus indicating potential biocompatibility *in vivo*.

### RZ2 induces apoptosis

**Fig. 5:**
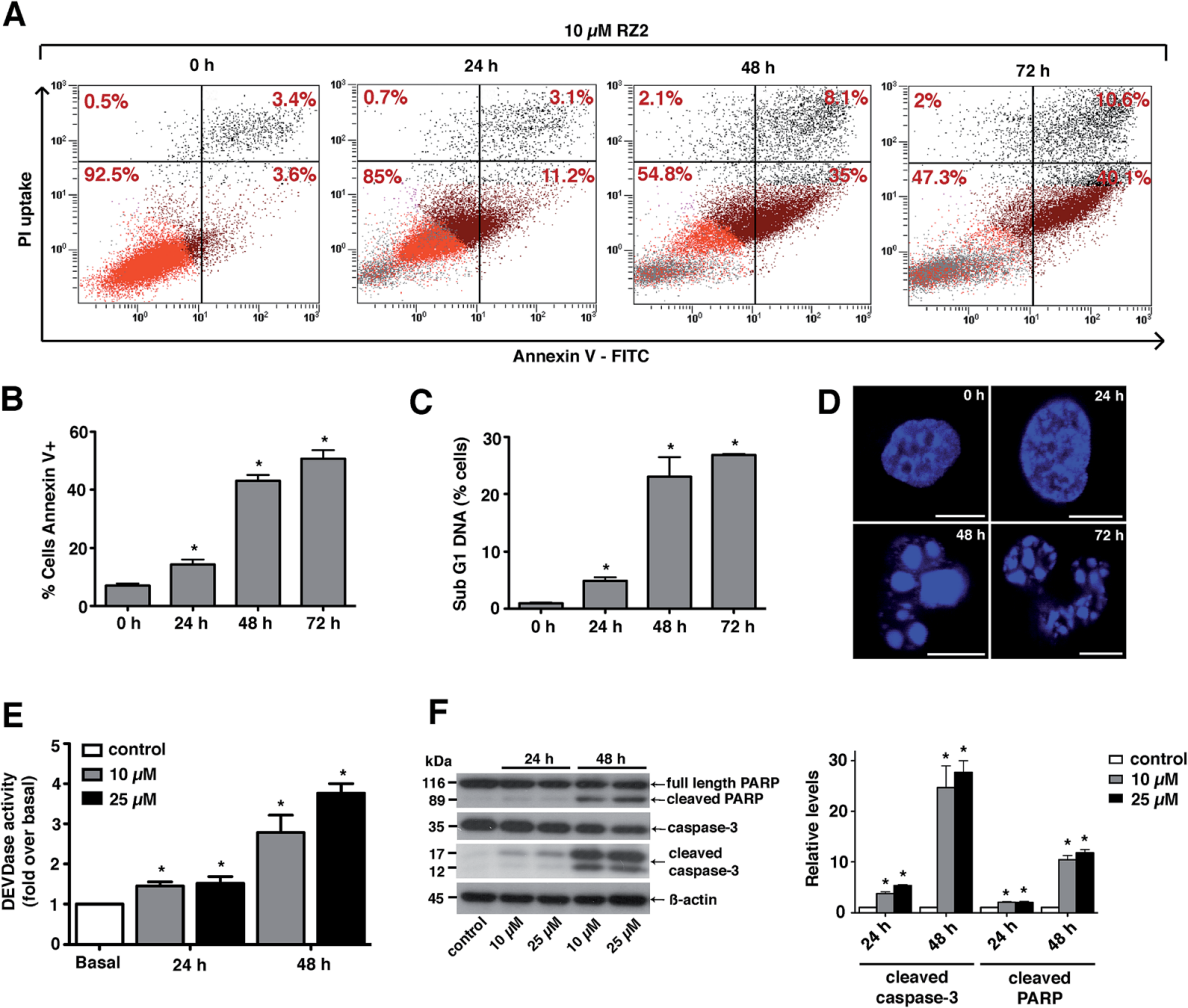
RZ2 induces apoptosis in HeLa cells. (A) Flow cytometry results of the effect of RZ2 (10 μM) on apoptosis and necrosis in HeLa cells. Total events are 10 000. The percentage of cells that are single, double positive or double negative for annexin V and PI are indicated in each grid. The upper left grid represents the number of single cells positive for PI. The lower right grid represents the number of cells positive for annexin V. The upper right grid represents the number of cells positive for both PI and annexin V. The lower left grid denotes the viable population. (B) HeLa cells were incubated for 0 h (control), 24 h, 48 h and 72 h with 10 μM RZ2 and stained for annexin V/PI. Data are mean ± s.e.m. (*n* = 3). *Significantly different from control (*P* < 0.05). (C) Flow cytometry analysis of the subG1 DNA fragmentation in ethanol-fixed HeLa cells after incubation with 10 μM RZ2 for 0 h (control), 24 h, 48 h and 72 h. Data are given as mean ± s.e.m. (*n* = 3). *Significantly different from control (*P* < 0.05). (D) Morphological analysis of nuclear fragmentation by Hoechst 33342 after incubation with 10 μM RZ2 for 0 h (control), 24 h, 48 h and 72 h. Pictures were taken using a Leica SP2 confocal microscope. Scale bars = 10 μm. (E and F) HeLa cells were treated with 10 μM and 25 μM RZ2 for 24 h and 48 h. Caspase activity was detected (E) by measurement of DEVD–AFC substrate processing and (F, left panel) total and cleaved caspase 3 and PARP levels were detected by western blot. Non-treated cells were used as control. (F, right panel) Densitometric quantification of cleaved caspase 3 and cleaved PARP levels (relative to control, non-treated cells). β-actin was used for loading normalization. Data are mean ± s.e.m. (*n* = 3). *Significantly different from control (*P* < 0.05).

Since RZ2 showed the highest cytotoxicity of all the compounds tested (Table 1), we studied whether it induces apoptosis in cancer cells. Because no single parameter defines this death pathway, we used a combination of measurements for the reliable detection of this process. In apoptotic cells, phosphatidylserine (PS) is translocated from the inner to the outer leaflet of the plasma membrane. Annexin V, a Ca^2+^-dependent phospholipid-binding protein, has a high affinity for PS and it is widely used to identify apoptotic cells.^39^ On the other hand, cells with plasma membrane permeabilization (dead cells) are permeant to propidium iodide (PI), which is internalized and binds to the nucleic acids in the cell.^40^ PI permeability assays couple to annexin V labeling in HeLa cells showed increased PI/annexin V labeling in response to RZ2 in a time-dependent manner (Fig. 5A). RZ2-specific induction of apoptosis was confirmed by an increase in annexin V-positive cells in response to the compound over time (Fig. 5B) and by an increase in the sub-G1 DNA fragmentation from 1% of the total cell population to 27% after 72 h of exposure (Fig. 5C). Using fluorescence staining, we further examined the morphological changes induced by RZ2 treatment. HeLa cells were stained with the fluorescent dye Hoechst 33342 and visualized. The nuclear fragmentation of apoptotic cells after 48 h of treatment with RZ2 was evident under the microscope (Fig. 5D), where the condensed chromatin areas were clearly distinguishable from the evenly distributed fluorescence occurring in control nuclei. Pyknosis and karyorrhexis were evident after 48 h and 72 h, but the nuclei subjected to 24 h treatment with RZ2 were intact, round and homogeneous. The latter observation, together with the low increase in annexin V-positive cells and only 5% sub-G1 DNA fragmentation after 24 h exposure to RZ2, suggests that apoptosis is activated around 24 h of treatment, reaching its full activation at 48 h.

The active forms of caspases 3 and 7 show specificity for cleavage at the *C*-terminus of the aspartate residue of the sequence DEVD (Asp–Glu–Val–Asp).^41^ The detection of DEVD hydrolysis is a reliable method for monitoring apoptosis induction and caspase 3/7 activity over time in living cells.^42^ Thus, we used the fluorogenic substrate Ac–DEVD–AFC as an indicator of the activation of effector caspases, which are the executioners of apoptosis.

For this purpose, we treated HeLa cells with two concentrations of RZ2 (10 and 25 μM) (Fig. 5E). Low, but significant increase in DEVDase activity was observed at 24 h with either of the concentrations tested, however, at 48 h of treatment there was a nearly 3-fold increase with 10 μM and a 4-fold increase with 25 μM RZ2. Furthermore, HeLa cells showed an increase in active (cleaved) caspase 3 levels as well as cleaved poly-(ADP-ribose) polymerase (PARP), another marker of apoptosis (Fig. 5F). All these observations confirm that HeLa cells undergo apoptosis upon 24 h of exposure to RZ2.

### RZ2 is internalized into acidic cell compartments

The fluorescent properties of RZ2 (*λ*_ex_ 323 nm; *λ*_em_ 418), due to the presence of two quinoxaline moieties in its structure, were not bright enough for visualization under the microscope without overlapping with cell autofluorescence. We therefore used 5-carboxyfluorescein (CF) (*λ*_ex_ 492 nm; *λ*_em_ 517 nm) for labeling purposes.

**Fig. 6:**
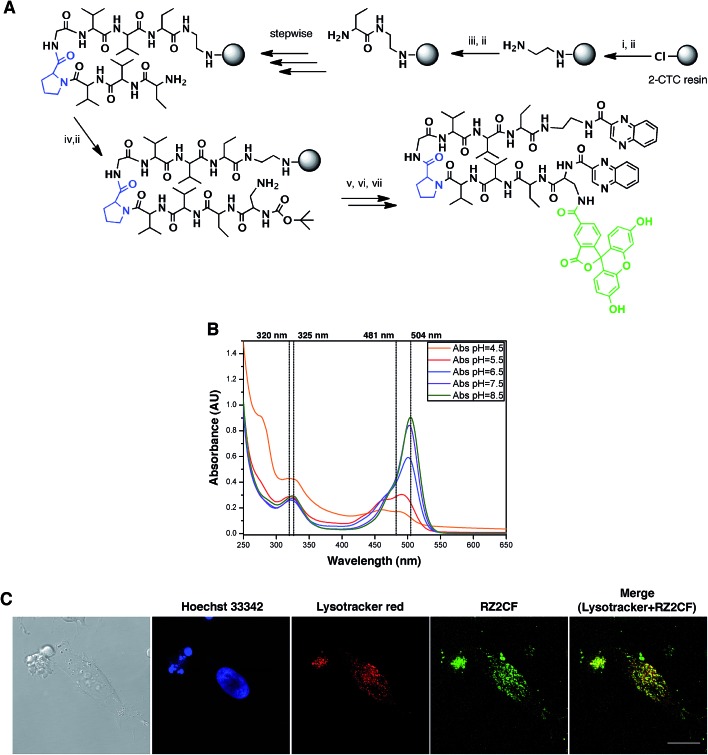
RZ2 is internalized into the cells in acidic compartments. (A) Solid phase synthesis of RZ2CF. (i) Fmoc–EDA·HCl, DIEA, CH_2_Cl_2_, 45 min; (ii) piperidine–DMF (1 : 4) (2 × 1 min, 2 × 5 min); (iii) Fmoc–Abu-OH, COMU, OxymaPure, DIEA and DMF, 1.5 h; (iv) Boc–Dap(Fmoc)-OH, COMU, OxymaPure, DIEA and DMF, 1.5 h; (v) 5-carboxyfluorescein, COMU, OxymaPure, DIEA and DMF, 1.5 h; (vi) TFA–H_2_O (3 : 2), 1 h; (vii) PyBOP, HOAt, 2-quinoxalinecarboxylic acid, DMF and CH_2_Cl_2_, pH 8. (B) Absorption spectra of RZ2CF (20 μM) in 2 mM sodium citrate buffer (pH 4.5) or 10 mM phosphate buffer (pH 5.5, 6.5, 7.5, and 8.5). (C) Confocal laser scanning microscopy of HeLa cells after 24 h of incubation with 25 μM of RZ2CF. Green fluorescence is due to the 5-carboxyfluorescein-labeled RZ2 compound. Scale bar = 20 μm. See ESI Movie S1.†

After several unsuccessful attempts to attach the CF through an ester bond between its carboxylic acid and the unprotected secondary alcohol of the serine's side chain, we substituted this amino acid residue for a diaminopropionic acid (Dap) in the RZ2 structure. The subsequent synthesis, labeling with CF and quinoxaline attachment proceeded without problems (Fig. 6A). After the final RP-HPLC purification step, product RZ2CF was obtained in excellent purity and evaluated *in vitro* for cytotoxic activity in HeLa cells using the MTT assay. Analysis of the dose-response data indicated that RZ2CF was still cytotoxic (IC_50_ = 16.7 μM) besides CF attachment.

In order to visualize whether RZ2CF is internalized into the cytosol, HeLa cells were incubated with a high concentration (50 μM) of RZ2CF for 24 h, and images were acquired with an inverted spinning disk microscope every 15 min (ESI Movie S1†). No accumulation of the compound was evident on the membrane or in the cytoplasm in the first 8 h of treatment.

Afterwards, some intense fluorescence was observed in small spherical granules within the cells. However, the presence of RZ2CF inside the cells was not unarguably evident until a significant increase in fluorescent vesicles was observed when the cytoskeleton of the apoptotic cells collapsed.

Since absorption and fluorescence properties of CF are strongly pH-dependent, we evaluated the absorption spectra of RZ2CF at a range of pH values. The absorbance of compound RZ2CF at pH values similar to those present in lysosomes (pH 4.5–5), late endosomes (pH 5–6), and early endosomes (6–7) was lower than at extracellular and cytosolic pH (7.5) (Fig. 6B). Hence, we argue that RZ2CF cannot be observed in cells while it is in acidic compartments until a dramatic change in their pH, as a result of the apoptotic process, enhances the fluorescence intensity of CF.

To examine colocalization of our compound with acidic compartment, HeLa cells were treated with 25 μM RZ2CF for 24 h, and live-cell confocal microscopy was performed using LysoTracker Red. Cell nuclei were stained with Hoechst 33342 (blue). Fluorescence in the green channel caused by RZ2CF colocalized with most of the compartments stained with LysoTracker, thereby suggesting that RZ2 exerts its cytotoxic action in acidic compartment (lysosomes, late endosomes) (Fig. 6C). On the basis of this last result, as well as on DNase I footprinting experiments that demonstrated that RZ2 does not bind DNA (Fig. S2†), we hold that the mechanism of action of our quinoxaline-containing peptide differs from that of bisintercalators such as triostin A, TANDEM, and echinomycin.

### RZ2 blocks autophagy

Autophagy is a degradation pathway, where cytoplasmic material is sequestered into the autophagosomes and subsequently degraded through fusion with lysosomes. Given our previous results in which RZ2 colocalizes with LysoTracker staining in HeLa cells (Fig. 6C), we examined the involvement of autophagy in RZ2-induced cell death.

Adaptor protein p62, also known as sequestosome-1, directly interacts with LC3 ^43^ and is a selective substrate for autophagy.

**Fig. 7:**
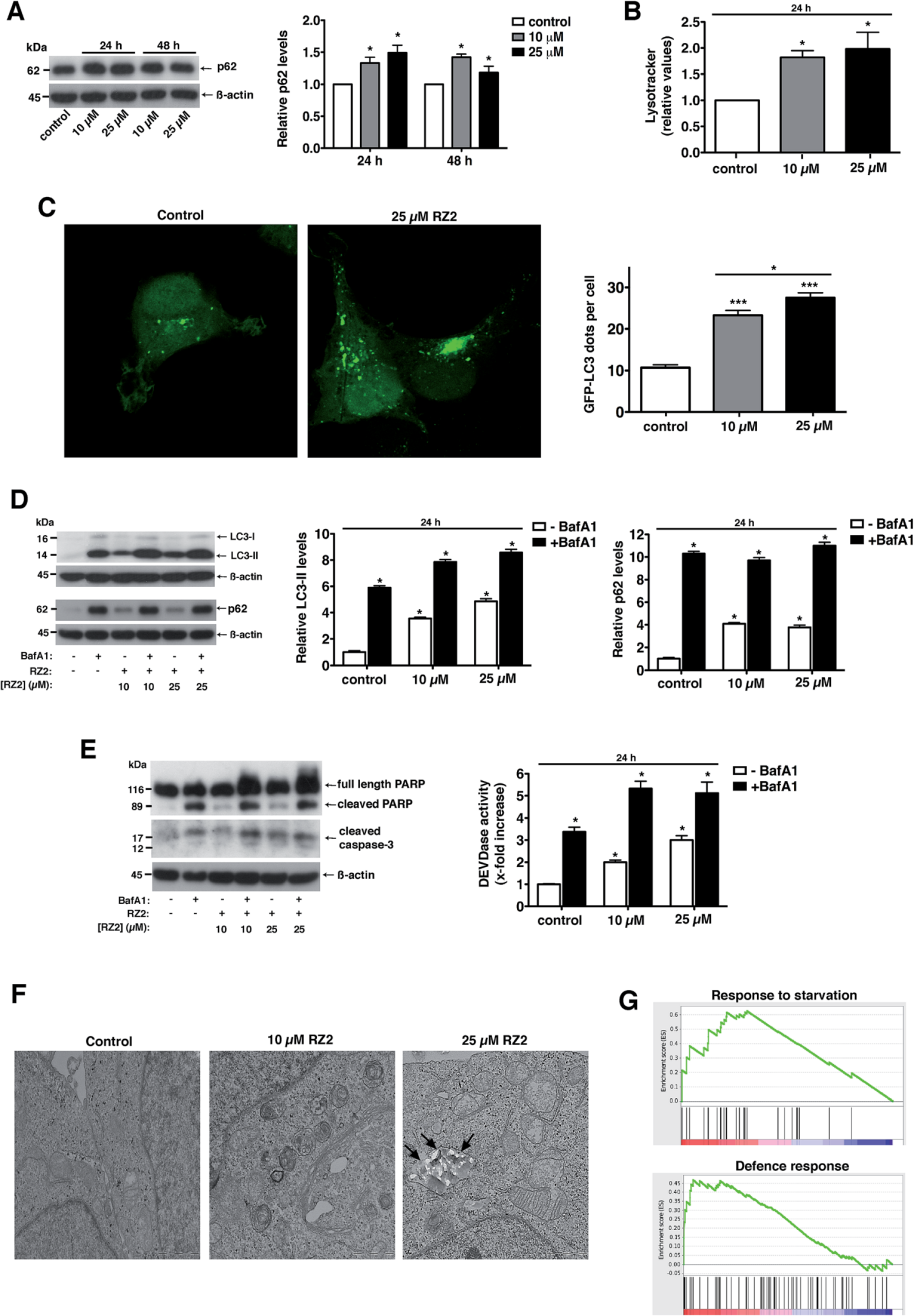
RZ2 modulates autophagy in HeLa cells. (A, left panel) HeLa cells were treated with 10 μM and 25 μM RZ2 for 24 h and 48 h. The expression of p62 was measured by western blot. (A, right panel) Densitometric quantification of p62 levels (relative to control, non-treated cells). β-actin was used for loading normalization. Data are mean ± s.e.m. (*n* = 3). *Significantly different from control (*P* < 0.05). (B) HeLa cells treated with 10 μM and 25 μM RZ2 for 24 h were stained with LysoTracker Green and analyzed by flow cytometry. Data are given as mean ± s.e.m. (*n* = 3). *Significantly different from control (*P* < 0.05). (C) HeLa cells were transfected with GFP-LC3 and 24 h after transfection treated with 10 and 25 μM RZ2 for 24 h. Pictures were taken with confocal microscopy (left panel) and number of GFP-LC3 dots per cell was counted (right panel). *Significantly different from control (*P* < 0.05). (D) HeLa cells were treated with 10 μM and 25 μM RZ2 for 24 h, in the presence or absence of 100 nM bafilomycin A1 (BafA1) for 16 h. LC3-II and p62 expression was measured by western blot (left panel). (D, right panels) Densitometric quantification of LC3-II and p62 protein levels (relative to control, non-treated cells). β-actin was used for loading normalization. Data are mean ± s.e.m. (*n* = 3). *Significantly different from compound RZ2 treated without BafA1 (*P* < 0.05). (E) HeLa cells were treated as in (D) and apoptosis was assessed by DEVDase activity (right panel) and western blot of PARP1 and caspase-3 (left panel). β-actin was used for loading normalization. Data are mean ± s.e.m. (*n* = 3). *Significantly different from compound RZ2 treated without BafA1 (*P* < 0.05). (F) Representative TEM pictures of HeLa cells left untreated or treated with 10 and 25 μM RZ2 for 24 h. (G) GSEA plots for the enrichment effects in response to 5 μM RZ2 treatment in HeLa cells for 24 h. See ESI Tables S2 and S3.†

Treatment with RZ2 increased p62 protein levels, thus suggesting impaired autophagy (Fig. 7A). This increase in p62 protein levels was not due to increased p62 gene expression (Fig. S5A†). Furthermore, RZ2 promotes formation of acidic compartment (Fig. 7B), which prompted us to check the formation of autophagosomes. HeLa cells were transfected with GFP-LC3 and LC3 positive dots were counted 24 h after RZ2 addition. As seen in Fig. 7C, RZ2 increased the number of LC3 dots per cell and also the protein levels of LC3-II and p62 (Fig. 7D). Addition of bafilomycin A1, that inhibits turnover of autophagosomes, further increased LC3-II and p62 protein levels compared to cells treated with RZ2 alone (Fig. 7D), indicating that RZ2 induces the abundance of autophagosomes without blocking their fusion with lysosomes. Of note, 16 h of bafilomycin A1 treatment *per se* induces apoptosis in HeLa cells (Fig. 7E), clearly indicating that autophagy plays a cytoprotective role in this model and its inhibition causes cell death. Combination of bafilomicin A1 and RZ2 did not have a synergistic effect, but rather an additive effect on caspase activation and DEVDase activity (Fig. 7E), suggesting similar mode of action; *i.e.* at the level of lysosomes. Moreover, given the increase in p62, the observation that RZ2 is not degraded by cathepsin B, and the significant accumulation of RZ2CF in acidic compartment (ESI Movie S1†), we postulate that RZ2 blocks autophagy by altering lysosomal function. To confirm this, we conducted a transmission electron microscopy (TEM) analysis of HeLa cells. In cells treated with 25 μM RZ2 we could observe vacuoles with accumulation of crystalline material (black arrows), clearly showing accumulation of the peptide inside them (Fig. 7F and S5B†). Furthermore, Atg5 knock-down (Atg5 KD) in HeLa cells did not rescue apoptosis (Fig. S5D†), suggesting that compound RZ2 does not target canonical autophagic pathway *per se*, but rather enters the cells through endocytic/endosomal pathway and accumulates in late endosomes/lysosomes. In parallel, we evaluated changes in gene expression before activation of the apoptotic pathway. For this purpose, global perturbations in genome-wide RNA expression in HeLa cells treated with a low concentration (5 μM) of RZ2 for 24 h were measured by gene expression microarray. The whole list of genes in the array (ranked by mean fold change against vehicle-treated cells, from most upregulated to most downregulated) (see ESI Table S2†) was analyzed against Human GO Biological Process and KEGG databases in order to detect overrepresented gene sets. A Gene Set Enrichment Analysis (GSEA) was used due to assess the group behaviour of a set of genes.^44^ Two of the biological processes were found to be enriched; the response to starvation (metabolic stress) and the defence response, (Fig. 7G; for a complete list of the enriched or depleted biological processes found in this experiment see ESI Table S3†). In cancer cells, which have a high metabolic rate, autophagy also provides metabolites to meet energy demands for rapidly proliferating cells. When cells are under stress, first, the autophagic response is activated as a strategy to adapt to and cope with the stress, blocking the induction of apoptosis. Then, when stress exceeds a critical duration or intensity, apoptosis is activated and caspases cleave several key autophagic proteins, shutting off the autophagic process.^10–13^ Altogether, our results show that RZ2 blocks autophagy at the level of lysosomal function, thus inducing metabolic stress (Fig. 7G), which leads to apoptosis activation.

### RZ2 induces apoptosis through mitochondrial pathway

Metabolic stress or any other cellular stress converges on mitochondria.

Damaged mitochondria are either degraded by autophagy (*i.e.* mitophagy) or induce intrinsic apoptotic signalling.^45^ Since we showed that RZ2 blocks autophagy, we hypothesized that RZ2 induces apoptosis through a mitochondrial pathway. To test this, we analysed the effect of RZ2 on mitochondrial membrane potential and superoxide mitochondrial levels using tetramethylrhodamine ethyl ester (TMRE) and MitoSOX Red dye, respectively.

**Fig. 8:**
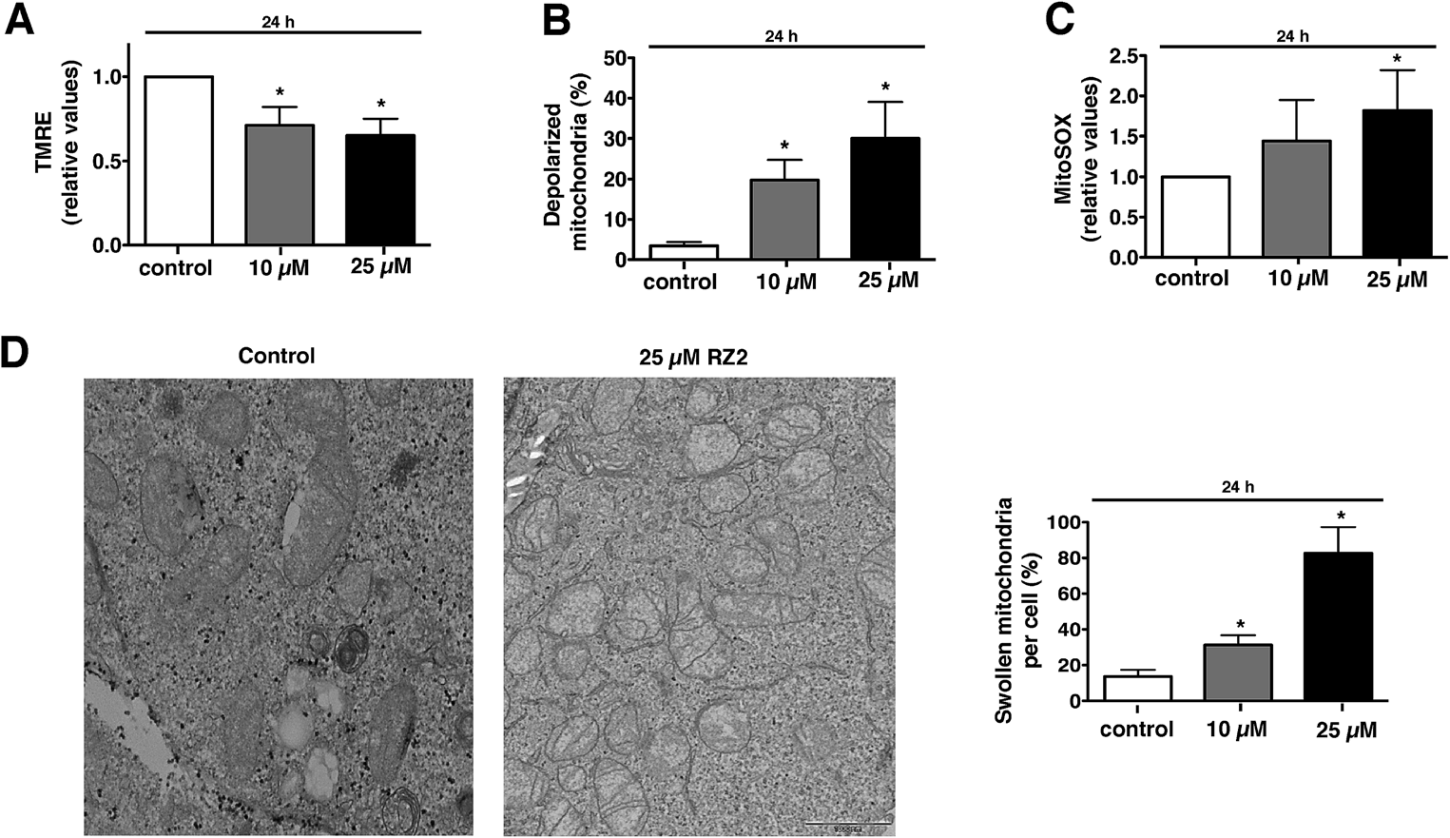
RZ2 induces apoptosis through mitochondria. (A and B) HeLa cells were treated with 10 μM and 25 μM RZ2 for 24 h, stained with TMRE and analyzed by flow cytometry to assess the (A) active mitochondria and (B) percentage of cells with completely depolarized mitochondria. Data are given as mean ± s.e.m. (*n* = 3). *Significantly different from control (*P* < 0.05). (C) HeLa cells were treated with 10 μM and 25 μM RZ2 for 24 h and analyzed by flow cytometry after addition of MitoSOX to assess superoxide mitochondrial production. Data are given as mean ± s.e.m. (*n* = 3). *Significantly different from control (*P* < 0.05). (D) Representative pictures of mitochondria in HeLa cells treated with 25 μM RZ2 for 24 h (left panel). Percentage of swollen mitochondria per cell was counted (right panel). *Significantly different from control (*P* < 0.05).

Mitochondria possess function-related membrane potentials. The dissipation of the inner mitochondrial transmembrane potential (Δ*ψ*_m_) marks the point-of-no-return during the apoptotic program.^46^ Mitochondrial depolarization is associated with outer mitochondrial membrane permeability.^47^ TMRE, a cell-permeable, positively-charged dye accumulates in active mitochondria as a result of its relative negative charge. Depolarized or inactive mitochondria have decreased membrane potential and fail to sequester TMRE. HeLa cells treated with 10 μM RZ2 had lower mitochondrial membrane potential than untreated cells (∼30% less), which was even more pronounced with 25 μM treatment (∼35% less) (Fig. 8A). Moreover, we also assessed percentage of cells with completely depolarized mitochondria; 19.8% of cells had depolarized mitochondria after 24 h when treated with 10 μM RZ2 and 30% with 25 μM (Fig. 8B), and the levels of mitochondrial ROS production were assessed using MitoSOX staining (Fig. 8C). TEM pictures further confirmed malfunctioning of mitochondria as increased swollen mitochondria were detected (Fig. 8D), and an increased mitochondrial abundance, not due to activated mitochondrial biogenesis, was found (Fig. S6†).

Accumulation of non-degradable material within lysosomes, as for example in lysosomal storage diseases, has been shown to cause oxidative stress and cell death predominantly due to impairment in quality control pathways and inability to recycle damaged organelles.^48^ We therefore propose that after 24 h of exposure to RZ2, the cytoprotective efforts of autophagy in HeLa cells are disrupted by the malfunction of the lysosomal machinery caused by the accumulation of RZ2 in acidic compartments. This disruption leads to a decrease in Δ*ψ*_m_ and also an increase in mitochondrial superoxide production and cell death is triggered *via* caspase activation.

## Experimental

### Compound syntheses and characterization

All peptides were synthesized by standard 9-fluorenylmethoxycarbony/*tert*-butyl (Fmoc/*t*Bu) SPPS. 2-chlorotrityl chloride resin was loaded with Fmoc-1,2-ethylenediamine·HCl (0.7 mmol g^–1^ resin) using DIEA in CH_2_Cl_2_. The peptide elongation was performed using COMU/OxymaPure/DIEA in DMF. Fmoc group removal was performed each time by treating the resin with piperidine (20%) in DMF (2 × 1 min, 2 × 5 min). Peptides were cleaved from the resin by treatment with TFA (20%) in CH_2_Cl_2_ at 25 °C and poured over H_2_O–ACN (1 : 1) to prevent cleavage of *t*Bu groups. The resulting solution was evaporated until reducing half the volume and then lyophilized. 2-quinoxalinecarboxylic acids were coupled at the *N*-terminus using PyBOP/HOAt/DIEA in DMF–CH_2_Cl_2_ (1 : 1). The solvent was evaporated, and the crude product was redissolved in CH_2_Cl_2_ and washed with saturated solutions of NH_4_Cl, NaHCO_3_ and NaCl, dried (MgSO_4_), and evaporated. Side-chain deprotection was accomplished by treatment with TFA–H_2_O (95 : 5) at 25 °C for 2 h. After global deprotection, the resulting solution was evaporated and lyophilized.

Compounds were purified to >90% purity by RP-HPLC (Waters 2545 binary gradient module, Waters 2998 photodiode detector equipped with a Waters 2767 sample manager) using a XBridge® BEH130 C18 column. UV detection was at 220 and 242 nm, and linear gradients of ACN (+0.036% TFA) into H_2_O (+0.045% TFA) were run at 3.0 mL min^–1^ flow rate. Different gradients were used depending on the profile of the crude product.

All final compounds were identified by MALDI-TOF and HR-ESMS (see ESI† for product characterization).

### Cell growth inhibition assays

Normally growing cells were plated at 5 × 10^3^ cells per well into 96-well plates and incubated for 24 h at 37 °C to allow attachment to the surface. Samples were then added dissolved in a DMSO–PBS vehicle (less than 1% in culture medium) at a range of concentrations. Drugs were run in triplicate or greater and control wells contained appropriate percentages of vehicle. After 24 h of exposure, the antitumor effect was measured using a solution of MTT, which is bioreduced by viable cells into formazan. The formed crystals were solubilized using DMSO and the amount of formazan was measured by reading the absorbance at 570 nm. The absorbance of wells containing only the MTT reagent (the plate blank) was subtracted from all wells.

The IC_50_ values were determined by dose response curve analysis and statistical analysis using GraphPad Prism software version 5.0a.

### Microarray data analysis

To examine global gene expression profiles, HeLa cells were treated with 5 μM RZ2 or with the vehicle for 24 h, followed by RNA preparation.

RNA expression profiling was performed following the Pico Profiling method.^49^ Each sample was hybridized to a GeneChip PrimeView Human Gene Expression Array (Affymetrix). Arrays were processed in bioconductor,^50^ using RMA background correction and summarization. Fold changes between samples were computed after MA mean and variance normalization using the GAM method. An empirical Bayes partial density model was then used to identify significant differentially expressed genes with a False Discovery Rate (FDR) of 5% and a log2 fold change threshold of 3 (8 times up- or down-regulated). The whole list of genes in the array (ranked by mean fold change from most up-regulated to most down-regulated) was analyzed against Human GO Biological Process and KEGG databases in order to detect overrepresented gene sets with a GSEA pre-ranked analysis.

Data were deposited in the NCBI GEO repository (accession number: GSE51948).

### Expression of results and statistical methods

Data are presented as mean ± s.e.m. of a number of 3 independent experiments. Data were subjected to analysis of variance, and comparisons between groups were performed using a protected Tukey's *t*-test. A value of *P* < 0.05 was chosen as the limit of statistical significance.

### Molecular dynamics simulations

Molecular dynamics simulations were run using the GPU-based PMEMD module of the Amber12 package. The parm99SBildn^51^ force field parameters were used to model the bond, angle and torsion energy terms and to assign charges and van der Waals radius to atoms. Point atomic charges of the quinoxaline ring were derived by fitting the molecular electrostatic potential determined at the Hartree–Fock 6-31G(d) level using the RESP procedure,^52^ and bonding parameters were obtained by analogy to the already parameterized atoms. The peptide was immersed in an octahedral box of *ca.* 5900 TIP3P waters^53^ with a minimum distance of 12 Å between an edge of the box and any atom of the peptide. A grid of 1 Å was used to compute the long-range electrostatic contribution and short-range interactions were truncated at 9 Å.

Each system was minimized following a three-step protocol, which involved the energy minimization of the hydrogen atoms, then peptide atoms, and finally the whole system including waters. Next, the systems were thermalized using the steps needed to achieve the desired temperature with a ramp of 50 K per every step of 100 ps. Different initial velocities were used for each independent simulation by choosing distinct random see numbers in the first thermalization step. The equilibrated systems were used as starting points for the unrestrained folding and Replica Exchange Molecular Dynamics (REMD) simulations. In all cases a 2 fs time step was used for integration.

The PMEMD.MPI module of Amber12 was used to run the REMD simulation with a temperature range exponentially spanning from 290.0 K to 398 K in 44 replicas to get a global exchange-acceptance rate around 30%. Each replica was independently thermalized to the desired temperature from the completely extended configuration, and then exchanges were attempted every 1 ps. Trajectories were run for 220 ns summing up a total simulation time of 9.68 μs.

## Conclusions

Here we designed and synthesized a small library of quinoxaline-containing peptides that resulted cytotoxic against the four human cancer cell lines tested. The most active compound, RZ2, was affordable in large quantities and displays low micromolar activity, especially against HeLa cells, where this hit molecule is endocyted into the cells and accumulates in acidic compartments blocking autophagy, which finally leads to cell apoptosis when the metabolic and oxidative stress cannot be repaired. Furthermore, RZ2 is stable under physiological conditions and in the presence of some proteases. This feature allows the peptide to remain intact in the presence of cathepsin B, which is located mainly in lysosomes. Therefore, the peptide cannot be hydrolyzed even when engulfed as autophagosomal content *via* autophagy. Taken together, our findings report a well-scalable peptidic compound decorated with two quinoxaline units that may be useful for clinical applications in cancer treatment and may contribute to the design of other molecules to investigate and develop antitumor chemotherapeutics.
