# Supplementary material for: A new quinoxaline-containing peptide induces apoptosis in cancer cells by autophagy modulation
Source: Chem Sci. 2015 May 20;6(8):4537–49. doi: 10.1039/c5sc00125k (PMC5666514; doi:10.1039/c5sc00125k)
Supplement: Supplementary file 2 [file SC-006-C5SC00125K-s002.pdf]

Electronic Supplementary Material (ESI) for Chemical Science.
This journal is © The Royal Society of Chemistry 2015

Supplementary Information

A New Quinoxaline-Containing Peptide Induces Apoptosis in Cancer Cells Through Autophagy Modulation

Rubí Zamudio-Vázquez, Saška Ivanova, Miguel Moreno, Maria Isabel Hernandez-Alvarez, Ernest Giralt, Axel Bidon-Chanal, Antonio Zorzano, Fernando Albericio, and Judit Tulla-Puche.

Inventory of supplementary data

Page

Figure S1. Chromatographic profiles of compounds RZ1-RZ12 .... S2

Figure S2. Compound RZ2 is not a DNA bisintercalator S3

Figure S3. Conformational preferences of compounds RZ1, RZ6 and RZ10 .... S5

Figure S4. Stability and biocompatibility assays of compound RZ2 .... S6

Figure S5. RZ2 affects autophagy S7

Figure S6. RZ2 does not increase mitochondrial biogenesis S8

Movie S1. Internalization of compound RZ2CF into HeLa cells S8
(File: Movie_RZ2CF_24h.avi)

Table S1. HR-ESMS characterization of compounds RZ1-RZ12 .... S9

Table S2. Genes significantly up or downregulated in the microarray data analysis S9
(File: Table_S2.xls)

Table S3. List of the biological processes found to be enriched or depleted in the microarray data analysis S10

Supplementary experimental procedures S13

Supplementary references S28

SUPPLEMENTARY DATA

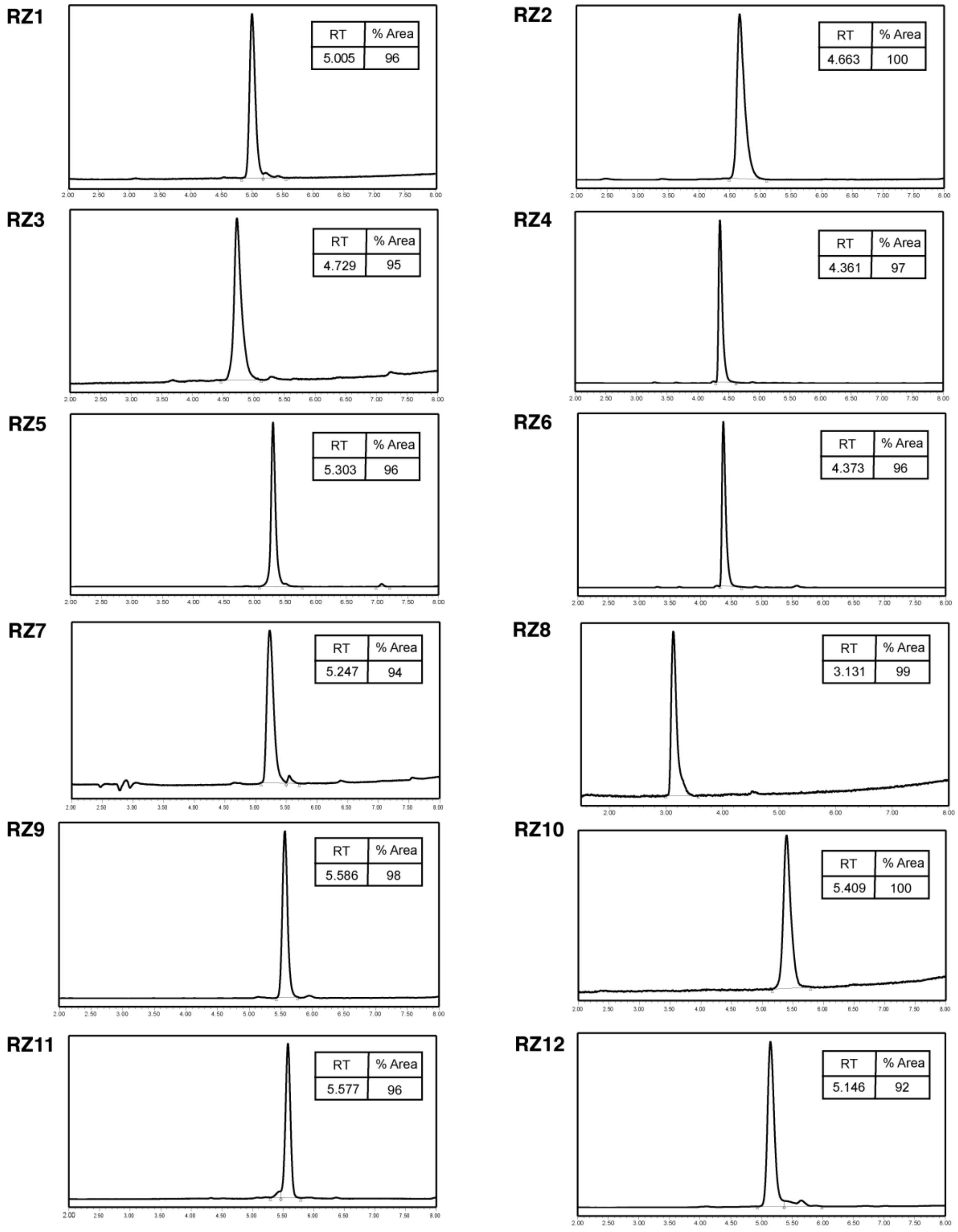

Figure S1. Chromatographic profiles of compounds RZ1-RZ12.

RP-HPLC analysis of the purified compounds used in biological assays.

A

MS1 5' - CCTAGGTATACGCCGTTATGTGTACCGGCTAAAGGTTGACGTGATCAGCATCGCGCTAGTTCCAATTCGAGGGCAAGATAGG
ACCATATCGTTAATCCCGCACTTCTCAATACATTTCATGCAGGCCACCCCAGACAAAACAGTAGAGTCGGAGCTTACGCCTAGG - 3'

HexA 5' - GGATCCCGGGATATCGATATATGGCGCCAAATTTAGCTATAGATCTAGAATTCCGGACCGCGGTTTAAACGTTAACCGGTACC
TAGGCCTGCAGCTGCGCATGCTAGCGCTTAAGTACTAGTGCACGTGGCCATGGATCC - 3'

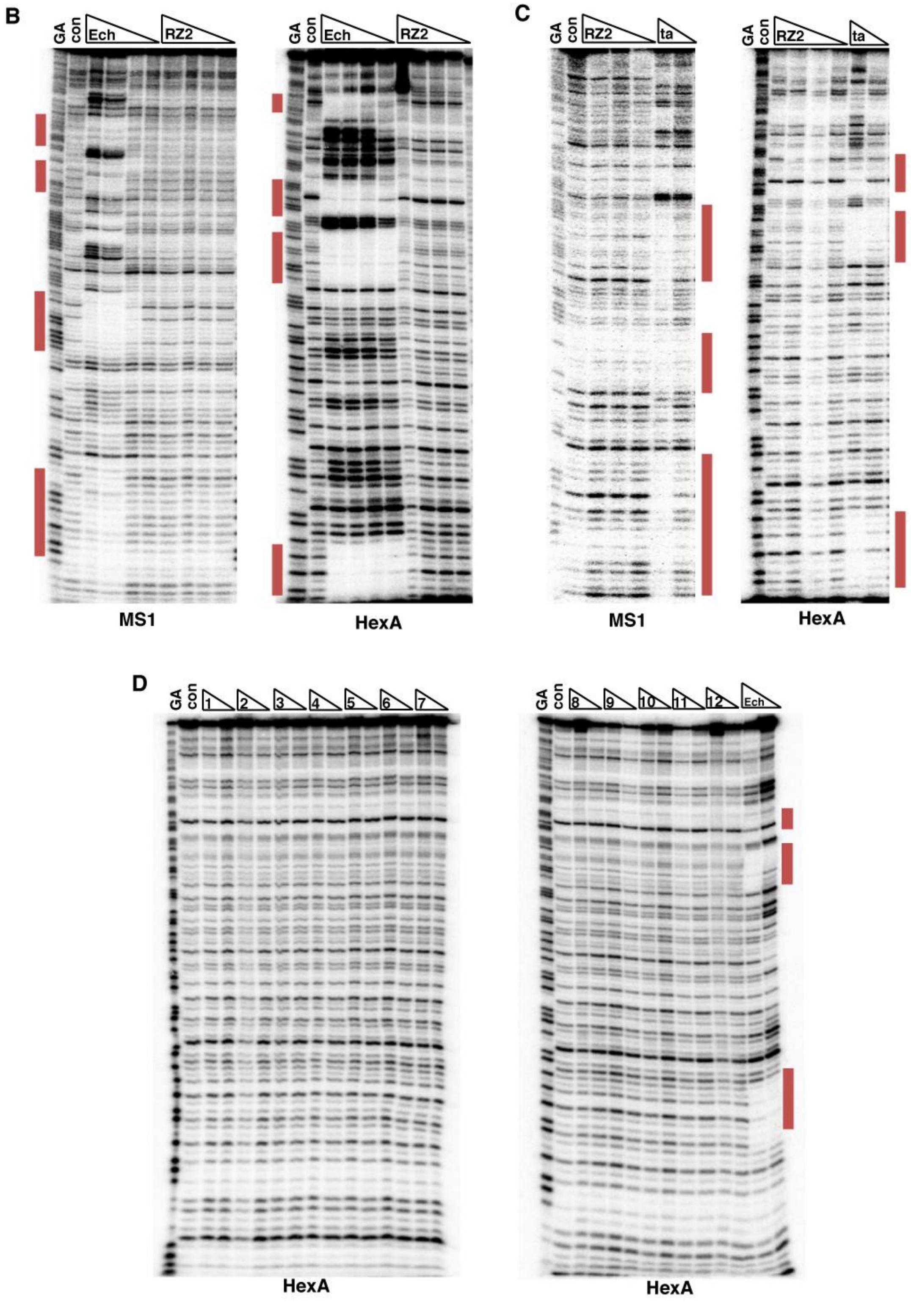

Figure S2. Compound RZ2 is not a DNA bisintercalator.

(A) Sequences of the universal footprinting substrates MS1 and HexA. The fragments were each labeled at the 3'-end and only the labeled strand is shown.

(B, C) DNase I cleavage pattern of MS1 and HexA in the presence of echinomycin (Ech), triostin A (ta) and compound RZ2. Tracks labeled "GA" are markers specific for purines. Tracks labeled "con" are the DNA incubated with the solvent conditions used for the drug. The bars shown alongside the gels indicate the regions of attenuated cleavage in the presence of the natural bisintercalators. (B) The concentrations of echinomycin and RZ2 are 1 mM, 100 μM, 10 μM and 1 μM. (C) The concentrations of RZ2 are 100 μM, 10 μM and 1 μM; the concentrations for triostin A are 100 μM and 10 μM.

(D) DNase I cleavage pattern of HexA in the presence of the RZ1-RZ12 library and echinomycin (Ech). Tracks labeled "GA" are markers specific for purines. Tracks labeled "con" are the DNA incubated with the solvent conditions used for the drug. Tracks labeled with numbers are the corresponding RZ compounds. The bars shown alongside the gels indicate the regions of attenuated cleavage in the presence of the natural bisintercalator. The concentrations of RZ compounds are 500 μM and 100 μM; the concentrations for echinomycin are 10 μM and 5 μM.

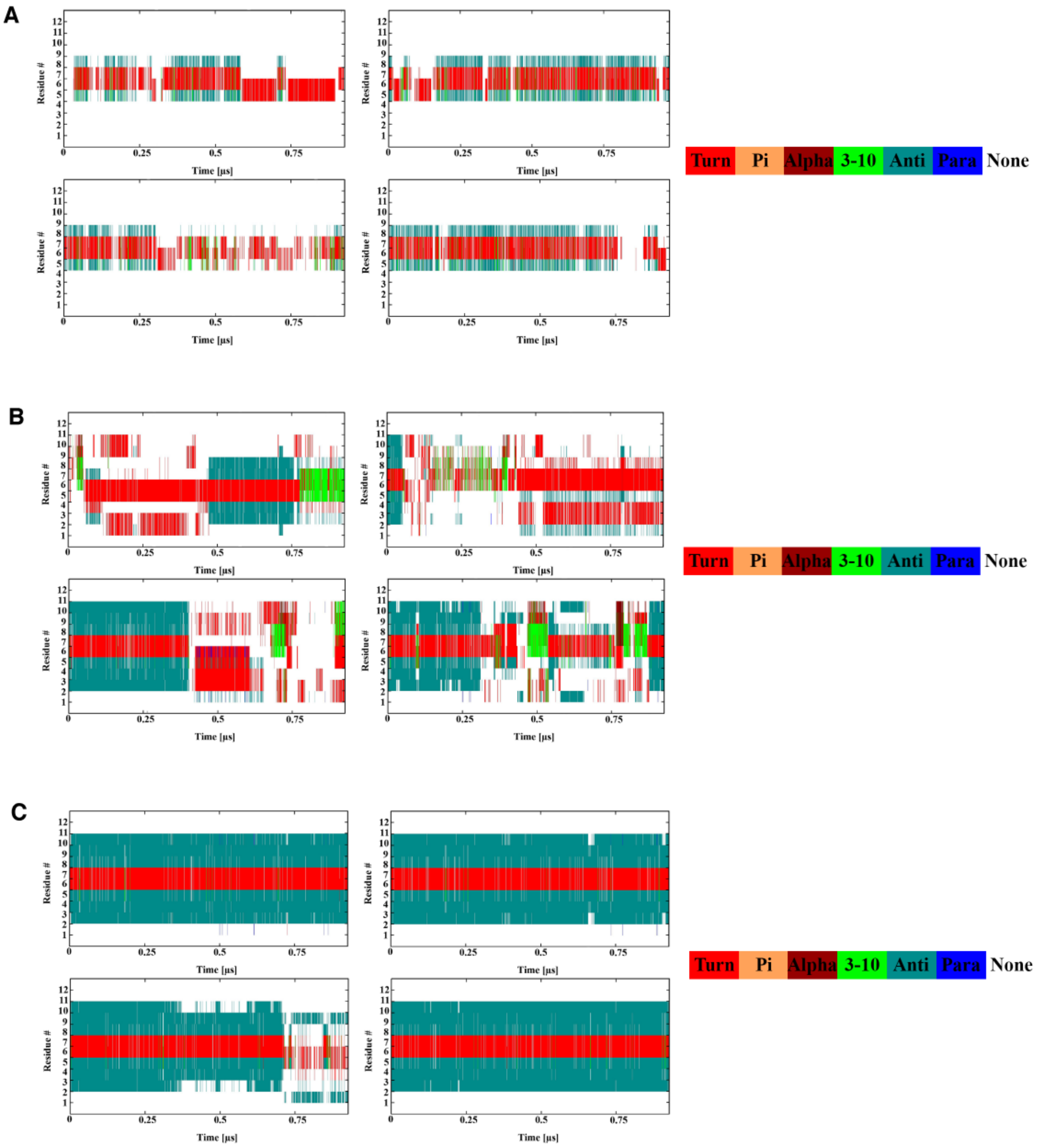

Figure S3. Conformational preferences of compounds RZ1, RZ6 and RZ10.

RZ1 (A) and RZ6 (B) show a marked tendency to loose the anti-parallel β-sheet conformation in all the simulations while in RZ10 (C) the tendency is completely reversed. Four simulations were run for each compound, one starting from a completely unfolded conformation and the other three from a folded conformation of compound RZ2.

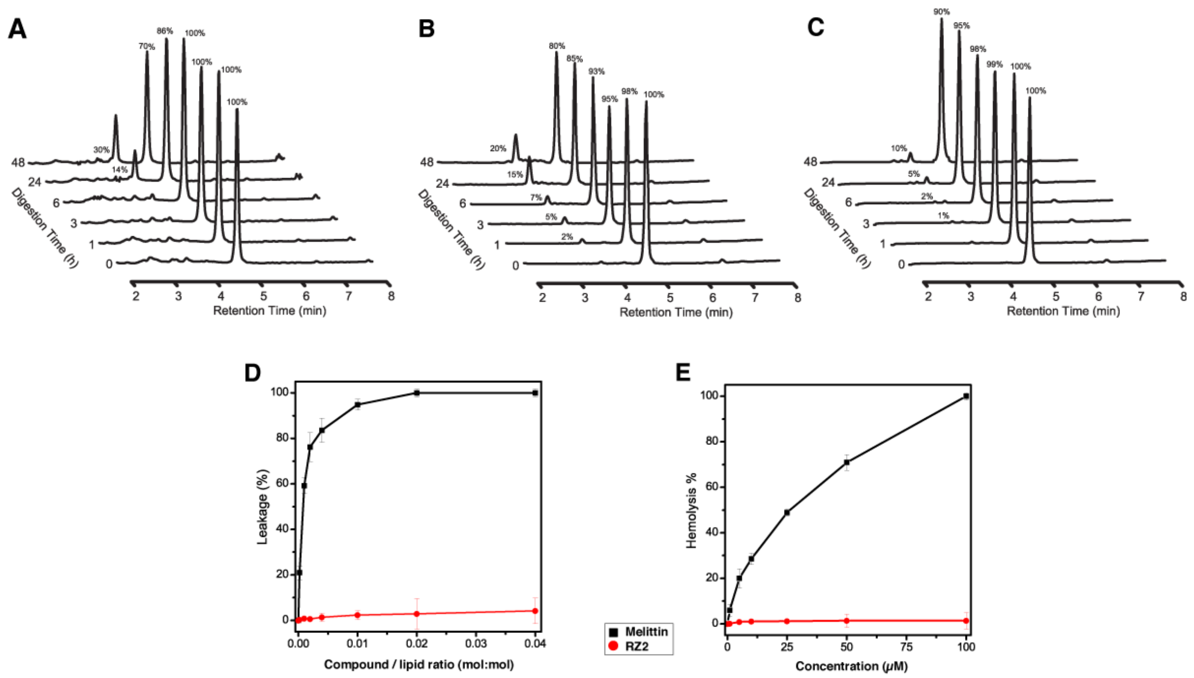

Figure S4. Stability and biocompatibility assays of compound RZ2.

(A, B, C) HLPC time study of digestion mediated by (A) human serum, (B) cathepsin B and (C) MMP-2 for RZ2.

(D) Effect on membrane rupture, i. e. leakage, of Large Unilamellar Vesicles (LUVs) containing EPC/Chol at molar ratio of 5:1 for 1 h at 37 ºC. Melittin was used as positive control. Data are expressed as mean±s.e.m. (n=3).

(E) Effect of RZ2 on hemoglobin release from red blood cells for 1 h at 37 ºC. Melittin was used as positive control. Data are expressed as mean±s.e.m. (n=3).

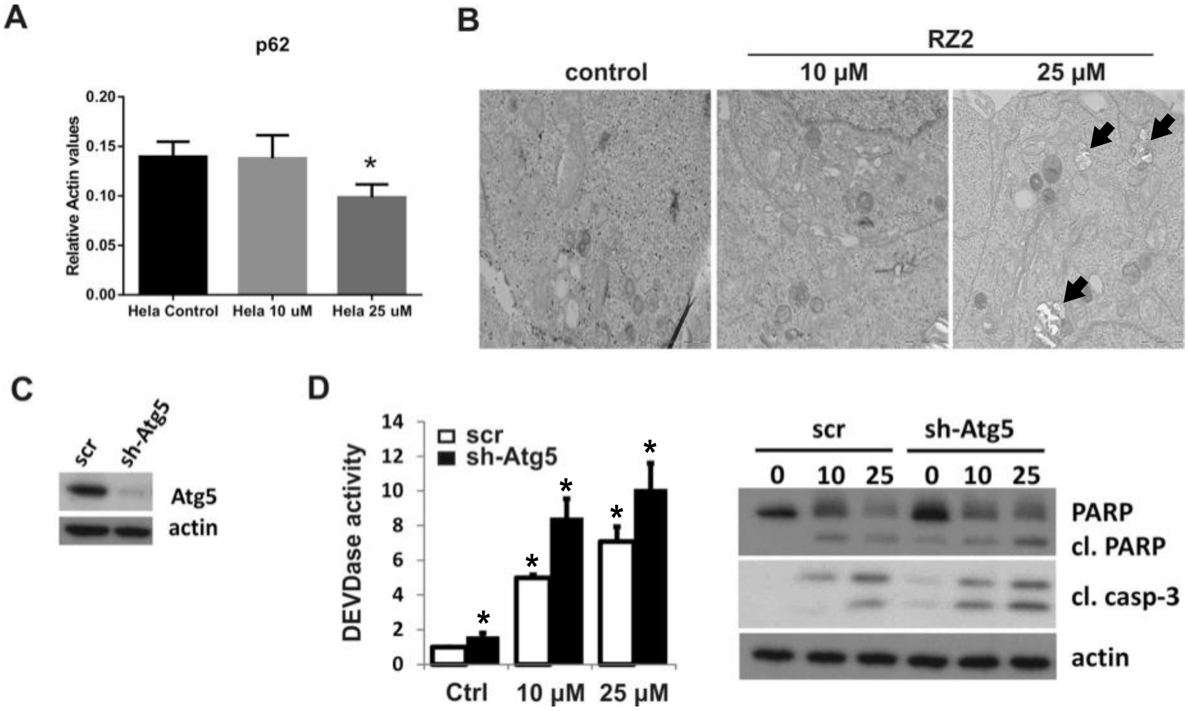

Figure S5. RZ2 affects autophagy.

(A) p62 mRNA levels were determined by RT-PCR. Data are expressed as mean±s.e.m. (n=3). *Significantly different from control (P<0.05).

(B) TEM pictures of vacuoles with accumulation of RZ2 (black arrows) 24 h after treatment with 10 and 25 μM compound.

(C, D) Atg5 was knock-downed in HeLa cells (C) and DEVDase activity and cleavage of PARP and caspase-3 were assessed (D). Data of DEVDase activity are expressed as mean±s.e.m. (n=3). *Significantly different from scr control HeLa cells (P<0.05).

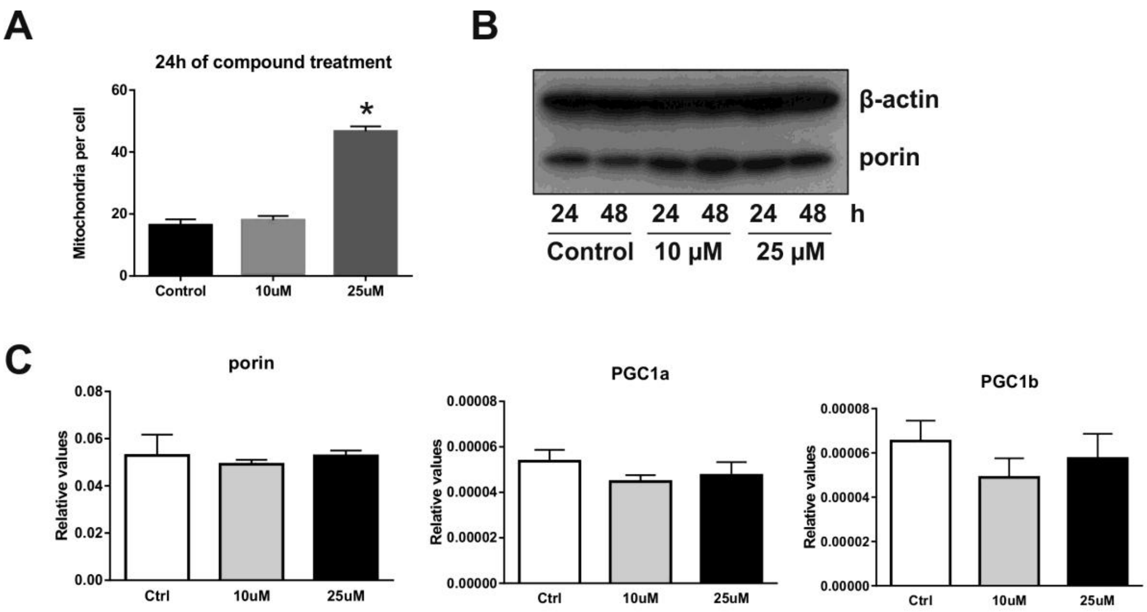

Figure S6. RZ2 does not increase mitochondrial biogenesis.

(A) Percentage of total number of mitochondria per cell (from TEM pictures). *Significantly different from control ($P<0.05$).

(B) HeLa cells were treated with 10 μM and 25 μM RZ2 for 24 h and analysed by Western blot for porin and β-actin.

(C) HeLa cells were treated with 10 μM and 25 μM RZ2 for 24 h and mRNA levels of porin, PGC1a and PGC1b were determined by RT-PCR.

File: Movie_RZ2CF_24h.avi

Movie S1. Internalization of compound RZ2CF into HeLa cells.

HeLa cells were incubated with 50 μM RZ2CF for 24 h, and images were acquired with an Andor "Revolution" inverted spinning disk microscope every 15 min.

Compound	Formula	*m/z* calculated	Mass found
RZ1	$C_{62}H_{89}N_{15}O_{12}$	1235.68097	1235.68006
RZ2	$C_{58}H_{81}N_{15}O_{12}$	1179.61836	1179.61954
RZ3	$C_{62}H_{89}N_{15}O_{12}$	1235.68097	1235.67801
RZ4	$C_{58}H_{81}N_{15}O_{12}$	1179.61836	1179.61917
RZ5	$C_{60}H_{85}N_{15}O_{14}$	1239.63949	1239.63906
RZ6	$C_{56}H_{77}N_{15}O_{14}$	1183.57689	1183.57278
RZ7	$C_{60}H_{85}N_{15}O_{14}$	1239.63949	1239.63764
RZ8	$C_{56}H_{77}N_{15}O_{14}$	1183.57689	1183.57781
RZ9	$C_{64}H_{93}N_{15}O_{12}$	1263.71227	1263.71240
RZ10	$C_{60}H_{85}N_{15}O_{12}$	1207.64967	1207.65062
RZ11	$C_{64}H_{93}N_{15}O_{12}$	1263.71227	1263.71376
RZ12	$C_{60}H_{85}N_{15}O_{12}$	1207.64967	1207.65076

Table S1. HR-ESMS characterization of compounds RZ1-RZ12.

HR-ESMS analyses of peptide samples were performed on a LTQ-FT Ultra (Thermo Scientific) mass spectrometer. Elemental compositions from experimental exact mass monoisotropic values were obtained with Xcalibur software (vs.2.0SR2).

File: Table_S2.xls

Table S2. Genes significantly up- or down-regulated in the microarray data analysis.

Column 5 μM RZ2 contains log2 RMA expression of genes in HeLa cells treated with RZ2, whereas column control contains the log2 RMA expression of genes in HeLa cells treated with the solvent control. The log2FC and FC columns indicate respectively log2 and decimal foldchange ratio between 5 μM RZ2 and control samples found after MA mean and variance normalization. ProbDE indicates empirical Bayes posterior probability of differential expression for each gene. Significant genes are those within a False Discovery Rate of 5% and a │log2FC│>3, that is, 8 or more times up- or down-regulated.

Enriched biological processes

Analyzed against Human GO Biological Process database

NAME	SIZE	ES	NES	NOM p-val	FDR q-val	FWER p-val
Negative regulation of viral genome replication	31	0.78148	2.3377488	0.0	0.0	0.0
Type I interferon-mediated signaling pathway	63	0.6223165	2.1376133	0.0	0.0013449127	0.003
Gluconeogenesis	43	0.6545361	2.0812266	0.0	0.0017658928	0.006
Glucose metabolic process	100	0.5201211	1.9662635	0.0	0.015336841	0.069
Tricarboxylic acid cycle	28	0.6652144	1.9528044	0.0	0.015737018	0.089
Glycogen catabolic process	17	0.7423281	1.9227481	0.0	0.020900121	0.138
Heart morphogenesis	33	0.6222864	1.8866948	0.0021141649	0.03054183	0.23
Cholesterol biosynthetic process	31	0.6195491	1.8463035	0.0	0.050183658	0.403
Detection of chemical stimulus involved in sensory	35	0.59830683	1.845602	0.004338395	0.045365784	0.407
Response to starvation	26	0.62742585	1.8224932	0.0	0.05745298	0.499
Cholesterol metabolic process	64	0.5222403	1.8058045	0.0021929825	0.06435228	0.566
Nucleosome disassembly	15	0.70671594	1.7638073	0.0022522523	0.09996373	0.753
2-Oxoglutarate metabolic process	15	0.6950013	1.7544839	0.006342495	0.103279024	0.789
Defense response to Gram-positive bacterium	35	0.57379705	1.7334262	0.0	0.120651476	0.855
Defense response to virus	134	0.44864517	1.7256721	0.0	0.12224841	0.882
Regulation of long-term neuronal synaptic plasticity	17	0.6614782	1.6878102	0.008264462	0.17050302	0.954
Glycolysis	44	0.52585274	1.6848214	0.004524887	0.16583481	0.958
Response to interferon-gamma	18	0.6420967	1.6800141	0.00814664	0.16316774	0.96
Response to virus	111	0.44196787	1.6724387	0.0	0.1669617	0.971
Trna aminoacylation for protein translation	41	0.5206481	1.6553028	0.008583691	0.18781683	0.989
Mesoderm development	29	0.5585808	1.6388875	0.014861995	0.20903493	0.997
Defense response	62	0.46768925	1.6212648	0.0065075923	0.23426242	1.0
Hexose transport	40	0.5199293	1.6208147	0.00856531	0.22479299	1.0
Glucose transport	57	0.48236126	1.6195284	0.0022123894	0.21754394	1.0
Cellular response to camp	24	0.5690456	1.6113114	0.010706638	0.22602929	1.0

Analyzed against KEGG database

NAME	SIZE	ES	NES	NOM p-val	FDR q-val	FWER p-val
Antigen processing and presentation	67	0.56711996	1.9694042	0.0	0.008319669	0.007
Citrate cycle (TCA cycle)	30	0.6613612	1.887204	0.0	0.016198507	0.027
Arginine and proline metabolism	53	0.5263432	1.7498868	0.0021978023	0.06352819	0.15
Alanine, aspartate and glutamate metabolism	32	0.5861432	1.7467111	0.002118644	0.04911635	0.155
Pentose phosphate pathway	27	0.6031213	1.7422475	0.0	0.04110694	0.162
Butanoate metabolism	30	0.58920115	1.7397012	0.008403362	0.035617013	0.168
Cysteine and methionine metabolism	36	0.552814	1.6946765	0.0061601643	0.05180359	0.261
Glycine, serine and threonine metabolism	32	0.56515574	1.6854066	0.004385965	0.049957566	0.284
Pyruvate metabolism	40	0.5274333	1.6726722	0.006666667	0.050190784	0.317
RNA transport	147	0.4243381	1.667155	0.0	0.048890114	0.34
Staphylococcus aureus infection	50	0.5078036	1.6595896	0.0043668123	0.04789632	0.362
DNA replication	36	0.52958566	1.6502188	0.0	0.049027342	0.389
Fc epsilon RI signaling pathway	76	0.46568626	1.6477689	0.0	0.046987664	0.404
Maturity onset diabetes of the young	24	0.5807541	1.6430821	0.0121951215	0.045871757	0.422
PPAR signaling pathway	69	0.47274315	1.6372145	0.0022371365	0.044800457	0.433
Steroid biosynthesis	19	0.6150128	1.6327245	0.018518519	0.044979498	0.452
Starch and sucrose metabolism	41	0.5027506	1.6081402	0.012631579	0.05354015	0.533
Mismatch repair	23	0.56066436	1.5932007	0.0186722	0.0580496	0.577
Graft-versus-host disease	36	0.50296897	1.5616245	0.018306635	0.07396781	0.673
Aminoacyl-tRNA biosynthesis	41	0.48372048	1.5290956	0.023255814	0.092925616	0.77
Carbohydrate digestion and absorption	40	0.4856828	1.5251759	0.023157895	0.09149643	0.777
Allograft rejection	35	0.48605764	1.5161515	0.036585364	0.09465729	0.804
Glyoxylate and dicarboxylate metabolism	18	0.581714	1.5144846	0.03837953	0.09232531	0.81
Fat digestion and absorption	44	0.46393788	1.4797084	0.018292682	0.11710185	0.896
Type I diabetes mellitus	40	0.47028983	1.4718544	0.019693654	0.11942241	0.919

Depleted biological processes

Analyzed against Human GO Biological Process database

NAME	SIZE	ES	NES	NOM p-val	FDR q-val	FWER p-val
Positive regulation of smooth muscle cell proliferation	40	-0.68550247	-2.1012874	0.0	0.0032914614	0.003
Actin filament bundle assembly	23	-0.72596234	-1.975864	0.0	0.033468474	0.058
Osteoblast differentiation	55	-0.6077046	-1.9740663	0.0	0.022681424	0.059
Positive regulation of cell death	17	-0.7698962	-1.9417777	0.0	0.03348752	0.115
Negative regulation of canonical Wnt receptor signaling	75	-0.5531178	-1.9010564	0.0	0.050461974	0.202
Neuron fate commitment	18	-0.72952	-1.8940889	0.0	0.048924215	0.228
Positive regulation of fibroblast proliferation	39	-0.60197735	-1.8527478	0.0	0.077671245	0.385
Positive regulation of cell migration	107	-0.5081286	-1.8291582	0.0	0.09294567	0.489
Cellular response to lipopolysaccharide	64	-0.5428616	-1.8166375	0.001754386	0.09708997	0.546
Positive regulation of collagen biosynthetic process	16	-0.72859824	-1.8145195	0.0	0.08963202	0.554
Response to nicotine	20	-0.69304776	-1.810995	0.0018450185	0.08463673	0.571
Negative regulation of BMP signaling pathway	32	-0.6188053	-1.8027955	0.0018484289	0.08784394	0.609
Response to wounding	52	-0.5552653	-1.8022307	0.0	0.08151701	0.611
Activation of MAPKK activity	46	-0.5567821	-1.7886662	0.0037950664	0.08847992	0.673
Positive regulation of angiogenesis	82	-0.50954443	-1.7815975	0.0	0.090419225	0.704
Keratinocyte differentiation	52	-0.54526466	-1.7757224	0.0018796993	0.09097363	0.73
Wound healing	61	-0.5415706	-1.7729709	0.001776199	0.088597	0.744
Response to progesterone stimulus	24	-0.6415636	-1.7707795	0.003992016	0.08631312	0.752
Positive regulation of endothelial cell migration	29	-0.6099705	-1.7676376	0.005791506	0.08513202	0.771
Cellular response to tumor necrosis factor	39	-0.5662046	-1.7267431	0.0035906644	0.1311437	0.908
Angiogenesis	200	-0.43098354	-1.7253217	0.0	0.12686282	0.911
Cellular response to transforming growth factor beta	31	-0.58403707	-1.7245928	0.0018691589	0.1223194	0.911
Negative regulation of neuron projection development	24	-0.6207839	-1.7238826	0.003868472	0.11788281	0.913
Positive regulation of tyrosine phosphorylation of STAT3	25	-0.61646545	-1.723497	0.011811024	0.113749705	0.914
Positive regulation of caspase activity	30	-0.60460305	-1.7232732	0.0058139535	0.109419934	0.914

Analyzed against KEGG database

NAME	SIZE	ES	NES	NOM p-val	FDR q-val	FWER p-val
Hypertrophic cardiomyopathy (HCM)	83	-0.4805204	-1.7229849	0.0	0.22669855	0.237
Focal adhesion	198	-0.42973024	-1.6892631	0.0	0.16908604	0.328
ECM-receptor interaction	83	-0.46843156	-1.6544653	0.0	0.16659679	0.45
Dilated cardiomyopathy	90	-0.46640205	-1.6402359	0.0034904014	0.14379661	0.505
Regulation of actin cytoskeleton	209	-0.40250874	-1.6015072	0.0	0.16906388	0.634
p53 signaling pathway	68	-0.45710045	-1.5446469	0.005628518	0.23524497	0.829
Pathogenic escherichia coli infection	54	-0.47557902	-1.5445068	0.015789473	0.20189527	0.83
African trypanosomiasis	33	-0.5121842	-1.5140245	0.0295858	0.22667436	0.903
Nicotinate and nicotinamide metabolism	24	-0.5284027	-1.4682057	0.06417112	0.2944122	0.966
Arrhythmogenic right ventricular cardiomyopathy (ARVC)	74	-0.41809237	-1.4397104	0.032846715	0.33283636	0.983
MAPK signaling pathway	266	-0.35635197	-1.4314214	0.0032733225	0.32307726	0.984
ErbB signaling pathway	86	-0.40528017	-1.4128402	0.035460994	0.33730334	0.993
Vascular smooth muscle contraction	113	-0.3815718	-1.4037989	0.018771332	0.33122876	0.996
Cytokine-cytokine receptor interaction	251	-0.34622884	-1.3995738	0.0049586776	0.31694412	0.996
Metabolism of xenobiotics by cytochrome P450	60	-0.41922906	-1.3972542	0.03314917	0.30057806	0.996
Circadian rhythm - mammal	21	-0.52086455	-1.391138	0.07453416	0.29437596	0.997
Shigellosis	59	-0.41932362	-1.3802059	0.06010929	0.29975033	0.999
Malaria	49	-0.43517372	-1.3716549	0.05950096	0.30014303	0.999
Ubiquitin mediated proteolysis	135	-0.35590848	-1.3628969	0.03130435	0.30144557	0.999
TGF-beta signaling pathway	84	-0.38812914	-1.3611718	0.06137184	0.2906321	1.0
Neurotrophin signaling pathway	126	-0.36456087	-1.3510429	0.035149384	0.2982771	1.0
Rheumatoid arthritis	86	-0.38484907	-1.3427604	0.07102804	0.30108902	1.0
Drug metabolism - cytochrome P450	60	-0.39869827	-1.3383447	0.069518715	0.29627332	1.0
Dorso-ventral axis formation	24	-0.48580304	-1.3218433	0.120792076	0.31575605	1.0
Gap junction	90	-0.36182046	-1.2875705	0.074600354	0.3795134	1.0

Table S3. List of the biological processes found to be enriched or depleted in the microarray data analysis.

Size describes the number of genes in the gene set after filtering out those genes not in the expression dataset. ES is the Enrichment Score for the gene set; that is, the degree to which this gene set is overrepresented at the top or bottom of the ranked list of genes in the expression dataset. NES is the Normalized Enrichment Score; that is, the enrichment score for the gene set after it has been normalized across analyzed gene sets. NOM p-val is the Nominal p value; that is, the statistical significance of the enrichment score. The nominal p value is not adjusted for gene set size or multiple hypothesis testing; therefore, it is of limited use in comparing gene sets. FDR q-val is the False Discovery Rate; that is, the estimated probability that the normalized enrichment score represents a false positive finding. FWER p-val is the FamilyWise-Error Rate; that is, a more conservatively estimated probability that the normalized enrichment score represents a false positive finding.

SUPPLEMENTARY EXPERIMENTAL PROCEDURES

Synthesis and characterization of the RZ1-RZ12 library

General

Protected amino acid derivatives and 2-CTC resin were obtained from Iris Biotech (Marktredwitz, Germany). DIEA, DIPCDI, piperidine, collidine, TFA, 2-quinoxalinecarboxylic acid and 5-carboxyfluorescein were obtained from Sigma-Aldrich (St. Louis, MO), PyBOP was from Shanghai Medpep (Shanghai, China) and HOAt, COMU and OxymaPure were from Luxembourg Industries (Tel Aviv, Israel). DMF, CH_2Cl_2, acetonitrile (HPLC grade), and methanol (HPLC grade) were obtained from SDS (Peypin, France). THF was obtained from Scharlau and purified using a Pure-Solv MD-2 solvent system (Innovative Technology, Inc.) All commercial reagents and solvents were used as received.

Solid-phase syntheses were performed in polypropylene syringes fitted with a polyethylene porous disc. Solvents and soluble reagents were removed by suction. Washings between deprotection and coupling steps were carried out with DMF (5 x 1 min) and CH_2Cl_2 (5 x 1 min) using 5 mL solvent$\cdot g^{-1}$ resin for each wash. Peptide synthesis transformations and washes were performed at 25 ºC unless indicated otherwise. Syntheses carried out on solid phase were controlled by HPLC of the intermediates obtained after cleaving an aliquot (~2 mg) of the peptidyl resin with TFA-TIS-H_2O (95:2.5:2.5) for 1 h.

Solution reactions were performed in round-bottomed flasks. Organic solvent extracts were dried over anhydrous $MgSO_4$, followed by solvent removal under reduced pressure.

SunFire® C18 reversed-phase HPLC analytical column (4.6 mm x 100 mm, 3.5 μm), Symmetry® C18 column (100mm x 19 mm, 5 μm), and XBridge® BEH130 C18 column, (10 mm x 100 mm, 5 μm) were obtained from Waters (Ireland).

Analytical RP-HPLC was carried out on a Waters instrument comprising a separation module

(Waters 2695), automatic injector, photodiode array detector (Waters 2998), and system controller (Millenium32 login). UV detection was at 220 and 242 nm, and linear gradients of ACN (+0.036% TFA) into H_2O (+0.045% TFA) were run at 1.0 mL·min^{-1} flow rate over 8 min.

RP-HPLC-ESMS was performed on a Waters ESI-MS Micromass ZQ spectrometer. Linear gradients of ACN (+0.07% formic acid) into H_2O (+0.1% formic acid) were run at 0.3 mL·min^{-1} flow rate over 8 min.

Semi-preparative HPLC was carried out on a Waters instrument comprising a binary gradient module (Waters 2545), photodiode detector (Waters 2998) equipped with a sample manager (Waters 2767) using an XBridge® BEH130 C18 column. UV detection was at 220 and 242 nm, and linear gradients of ACN (+0.036% TFA) into H_2O (+0.045% TFA) were run at 3.0 mL·min^{-1} flow rate. Different gradients were used depending on the profile of the crude.

MALDI-TOF and ES(+)-MS analyses of peptide samples were performed on an Applied Biosystems VoyagerDE RP, using ACH matrix, and in a Waters Micromass ZQ spectrometer and in an Agilent Ion Trap 1100 Series LC/MSDTrap.

HR-ESMS analyses of peptide samples were performed on a LTQ-FT Ultra (Thermo Scientific) mass spectrometer. Samples for exact mass determination were prepared dissolving in H_2O/ACN (1:4) and diluting 1/100 with H_2O/ACN with 0.1% formic acid.

Incorporation of ethylenediamine on 2-CTC resin

Fmoc-1,2-ethylenediamine·HCl (0.7 mmol/g resin) was dissolved in CH_2Cl_2 (5 mL/g resin) and DIEA (9 mmol/g resin) and was added to the resin. The reaction was shaken at 25 ºC for 45 min. Next, the resin was capped by the addition of MeOH (0.8 μL/mg resin) for 10 min at 25 ºC, and the resin was washed with CH_2Cl_2 (5 x 1 min) and with DMF (5 x 1 min). Fmoc quantitation gave a loading of 0.7 mmol/g.

Removal of the Fmoc group

The Fmoc group was removed with piperidine-DMF (1:4, v/v) (10 mL/g resin) (2 x 1 min, 2 x 5 min).

Incorporation of Fmoc-AA-OH

(Fmoc-Abu-OH, Fmoc-*N*Me-Val-OH, Fmoc-Val-OH, Fmoc-Thr(*t*Bu)-OH, Fmoc-Ile-OH, Fmoc-Gly-OH, Fmoc-D-Pro-OH, Fmoc-Ser(*t*Bu)-OH, Fmoc-D-Ser(*t*Bu)-OH). Fmoc-AA-OH (4 equiv), COMU (4 equiv), OxymaPure (4 equiv), DIEA (8 equiv) in DMF (10 mL/g resin) were incorporated with preactivation for 30 s at 25 ºC and left to stand for 1.5 h.

When coupling Fmoc-*N*Me-Val-OH on *N*Me-Abu-OH, a β-branched amino acid on *N*Me-Val-OH and vice versa, the reaction mixture was transferred to a glass tube and reaction temperature was raised up to 50 ºC and left to stand for 1.5 h. One recoupling was always performed under the last same conditions.

Amino acid N-alkylation

This process was divided into 3 steps:[1]

a) Protection and activation with o-NBS

o-NBS-Cl (4 equiv) and 2,4,6-collidine (10 equiv) in CH_2Cl_2 were added to the resin. The reaction was gently stirred for 1.5 h. The reaction was monitored by the ninhydrin test.

b) Deprotonation and methylation

Triphenylphosphine (5 equiv) in MeOH (10 equiv) and anhydrous THF (5 mL/g resin) were added to the resin and left for 1 min under N_2 atmosphere. Then, DIAD (5 equiv) was carefully added and left for 20 min under N_2 atmosphere.

c) o-NBS removal

To remove o-NBS, β-mercaptoethanol (10 equiv) and DBU (5 equiv) in DMF were added to the resin and the mixture was left to react for 15 min. This operation was repeated twice.

Cleavage from the resin without peptide's side-chains deprotection

After CH_2Cl_2 washing, the resin was cleaved using 20% TFA in CH_2Cl_2 (10 mL/g resin, 10x30 s) at 25 ºC and poured over H_2O-ACN (1:1) to avoid cleavage of *t*Bu groups. The resulting solution was evaporated until reducing half of the volume, and lyophilized.

2-quinoxalinecarboxylic acid introduction in solution and final deprotection

2-quinoxalinecarboxylic acid (2.4 equiv), PyBOP (2.4 equiv), HOAt (2.4 equiv) and DIEA (until pH 8) were dissolved in DMF-CH_2Cl_2 (1:1) and stirred at 25 ºC for 35 hours. The solvent was evaporated, redissolved in CH_2Cl_2 and washed with saturated solutions of NH_4Cl, $NaHCO_3$ and NaCl, dried ($MgSO_4$), and evaporated. The side-chains' deprotection was accomplished by treatment with TFA-H_2O (95:5) at 25 ºC for 2 h. After global deprotection, the resulting solution was evaporated and lyophilized.

Example: Synthesis of RZ1

2-CTC resin (150 mg, 1.56 mmol/g) was placed in a 10-mL polypropylene syringe fitted with a polyethylene filter disc. It was then washed with DMF (5 x 1 min) and CH_2Cl_2 (5 x 1 min). Fmoc-1,2-ethylenediamine·HCl (33.5 mg, 0.105 mmol) and DIEA (230 μL) in CH_2Cl_2 (1.5 mL) was added to the resin. The mixture was shaken at 25 ºC for 45 min. The reaction was quenched by addition of MeOH (120 μL) and the mixture was stirred for 10 min at 25 ºC. After filtration, the resin was washed with CH_2Cl_2 (5 x 1 min) and with DMF (5 x 1 min) and cleavage of the Fmoc group was done by treatment with piperidine-DMF (1:4) (3.75 mL) (2 x 1 min, 2 x 5 min). The filtrates were collected and quantified by UV (290 nm) obtaining a loading of 0.70 mmol/g. Based on this loading, Fmoc-Abu-OH (136.7 mg, 0.42 mmol) was incorporated with COMU (179.9 mg, 0.42 mmol), OxymaPure (59.7 mg, 0.42 mmol) and DIEA (142.9 μL, 0.84 mmol) in DMF (10 mL/g resin) with preactivation for 30 s at 25 ºC and stirred for 1.5 h. After resin filtration and washings with DMF (5 x 1 min) and CH_2Cl_2 (5 x 1 min) the Fmoc group was

removed as described above and the resin was washed again. A solution of *o*-NBS-Cl (93 mg, 0.42 mmol) and 2,4,6-collidine (138.8 μL, 1.05 mmol) in CH_2Cl_2 was added to the resin and the mixture stirred for 90 min. After filtration and washings with CH_2Cl_2 (5 x 1 min), DMF (5 x 1 min), CH_2Cl_2 (5 x 1 min) and THF (5 x 1 min), a solution of PPh_3 (137.7 mg, 0.525 mmol) and MeOH (42.6 μL, 1.05 mmol) in THF, and a solution of DIAD (101.8 μL, 0.525 mmol) in THF were mixed and added to the resin under N_2 atmosphere. After stirring the resin for 20 min, it was filtered and washed with THF (5 x 1 min), CH_2Cl_2 (5 x 1 min) and DMF (5 x 1 min). After removal of the *o*-NBS group with DBU (78.7 μL, 0.525 mmol) and 2-mercaptoethanol (73.6 μL, 1.05 mmol) in DMF (3 x 15 min), the resin was washed with DMF (5 x 1 min), CH_2Cl_2 (5 x 1 min), and DMF (5 x 1 min). Fmoc-*N*Me-Val-OH (148.4 mg, 0.42 mmol) was incorporated with COMU (179.9 mg, 0.42 mmol), OxymaPure (59.7 mg, 0.42 mmol) and DIEA (142.9 μL, 0.84 mmol) in DMF (10 mL/g resin) at 50 ºC. After stirring for 90 min, the resin was filtered and washed with DMF (5 x 1 min) and CH_2Cl_2 (5 x 1 min) and one recoupling was performed under the same conditions. After resin filtration, and further removal of the Fmoc group, the resin was washed again. Next, Fmoc-Val-OH (142.6 mg, 0.42 mmol) was incorporated with COMU (179.9 mg, 0.42 mmol), OxymaPure (59.7 mg, 0.42 mmol) and DIEA (142.9 μL, 0.84 mmol) in DMF (10 mL/g resin) at 50 ºC. After stirring for 90 min, the resin was filtered and washed with DMF (5 x 1 min) and CH_2Cl_2 (5 x 1 min) and one recoupling was carried out under the same conditions. After resin filtration, the Fmoc group was removed and the resin was washed again. The following protected amino acids (Fmoc-Gly-OH, Fmoc-D-Pro-OH, Fmoc-Val-OH, Fmoc-*N*Me-Val-OH) were incorporated with COMU (4 equiv), OxymaPure (4 equiv) and DIEA (8 equiv) in DMF stirring for 90 min at room temperature. Fmoc-Abu-OH (136.7 mg, 0.42 mmol) was incorporated with COMU (179.9 mg, 0.42 mmol), OxymaPure (59.7 mg, 0.42 mmol) and DIEA (142.9 μL, 0.84 mmol) in DMF (10 mL/g resin) with preactivation for 30 s at 50 ºC and stirred for 1.5 h. One recoupling was done. The *N*-alkylation of this residue was accomplished under the same conditions described above and after removal of the *o*-NBS group, the resin was washed, and Fmoc-Ser(*t*Bu) (161.1 mg, 0.42 mmol) was incorporated with COMU (179.9 mg, 0.42 mmol), OxymaPure (59.7 mg, 0.42 mmol) and DIEA (142.9 μL, 0.84 mmol) in DMF (10 mL/g resin) at 50 ºC. No recoupling was necessary at this time. After finishing the elongation, the peptide was cleaved with 20% TFA in CH_2Cl_2 (4.5 mL, 10 x 30 s) at 25 ºC and poured over H_2O-ACN (1:1)

(50 mL) to avoid cleavage of the *t*Bu groups. The resulting solution was partially evaporated and lyophilized (102.4 mg; 99.5% yield).

An aliquot was subjected to analytical HPLC (linear gradient from 15:85 to 40:60 (0.036% TFA in ACN/0.045% TFA in H_2O) in 8 min at T = 25 °C; t_R = 5.56 min; 72% purity) and HPLC-ESMS (gradient from 15:85 to 40:60 (0.07% HCO_2H in ACN/0.1% HCO_2H in H_2O) in 8 min; t_R = 4.03 min; *m/z* calculated for $C_{48}H_{89}N_{11}O_{10}$, 979.68; found, 980.33 $[M + H]^+$; found, 490.79 [(M + 2)/2]

The peptide (0.1 mmol) was dissolved in DMF-CH_2Cl_2 (1:1) (10 mL), and PyBOP (124.9 mg, 0.24 mmol), HOAt (32.7 mg, 0.24 mmol) and 2-quinoxalinecarboxylic acid (41.8 mg, 0.24 mmol) were added to the solution. The pH was adjusted to 8 by adding DIEA and the mixture was stirred until HPLC analysis indicated the completion of the reaction. The solvent was evaporated under reduced pressure and the peptide was redissolved in CH_2Cl_2 (30 mL). The organic layer was washed with saturated solutions of NH_4Cl (1 x 20 mL), $NaHCO_3$ (1 x 20 mL) and brine (1 x 20 mL, dried with $MgSO_4$ and evaporated under vacuum.

Total deprotection was accomplished by treatment with TFA-H_2O (95:5; 40 mL) at 25 ºC for 2 h. After global deprotection, the resulting solution was evaporated to 5 mL and lyophilized.

The crude peptide was purified by semi-preparative reversed HPLC (linear gradient from 40:60 to 45:55 (0.036% TFA in ACN/0.045% TFA in H_2O) over 10 min; flow rate 3 mL/min; temperature 30 ºC) to afford 19.6 mg (15% overall yield) of the pure compound obtained as white powder.

Characterization by analytical HPLC (linear gradient from 30:70 to 100:0 (0.036% TFA in ACN/0.045% TFA in H_2O) in 8 min; t_R = 5.0 min); HPLC-ESMS (gradient from 30:70 to 100:0 (0.07% HCO_2H in ACN/0.1% HCO_2H in H_2O) in 8 min; t_R = 6.0 min; *m/z* calculated for $C_{62}H_{89}N_{15}O_{12}$, 1235.68; found, 1237.97 $[M + H]^+$; HR-ESMS: *m/z* calculated for $C_{62}H_{89}N_{15}O_{12}$, 1235.68097; found 1235.68006.

RZ2

The product was obtained as white powder in overall yield 12.1%.

Characterization by analytical HPLC (linear gradient from 30:70 to 100:0 (0.036% TFA in ACN/0.045% TFA in H_2O) in 8 min; t_R = 4.6 min); HPLC-ESMS (gradient from 30:70 to 100:0 (0.07% HCO_2H in ACN/0.1% HCO_2H in H_2O) in 8 min; t_R = 5.8 min; *m/z* calculated for $C_{58}H_{81}N_{15}O_{12}$, 1179.62; found, 1180.89 $[M + H]^+$; HR-ESMS: *m/z* calculated for $C_{58}H_{81}N_{15}O_{12}$, 1179.61836; found 1179.61954.

RZ3

The product was obtained as white powder in overall yield 0.5%.

Characterization by analytical HPLC (linear gradient from 30:70 to 100:0 (0.036% TFA in ACN/0.045% TFA in H_2O) in 8 min; t_R = 4.7 min); HPLC-ESMS (gradient from 30:70 to 100:0 (0.07% HCO_2H in ACN/0.1% HCO_2H in H_2O) in 8 min; t_R = 6.1 min; *m/z* calculated for $C_{62}H_{89}N_{15}O_{12}$, 1235.68; found, 1237.13 $[M + H]^+$; HR-ESMS: *m/z* calculated for $C_{62}H_{89}N_{15}O_{12}$, 1235.68097; found 1235.67801.

RZ4

The product was obtained as white powder in overall yield 1.5%.

Characterization by analytical HPLC (linear gradient from 30:70 to 100:0 (0.036% TFA in ACN/0.045% TFA in H_2O) in 8 min; t_R = 4.3 min); HPLC-ESMS (gradient from 30:70 to 100:0 (0.07% HCO_2H in ACN/0.1% HCO_2H in H_2O) in 8 min; t_R = 5.4 min; *m/z* calculated for $C_{58}H_{81}N_{15}O_{12}$, 1179.62; found, 1182.08 $[M + H]^+$; HR-ESMS: *m/z* calculated for $C_{58}H_{81}N_{15}O_{12}$, 1179.61836; found 1179.61917.

RZ5

The product was obtained as white powder in overall yield 11.7%.

Characterization by analytical HPLC (linear gradient from 20:80 to 80:20 (0.036% TFA in ACN/0.045% TFA in H_2O) in 8 min; t_R = 5.3 min); HPLC-ESMS (gradient from 30:70 to 100:0 (0.07% HCO_2H in ACN/0.1% HCO_2H in H_2O) in 8 min; t_R = 4.3 min; *m/z* calculated for

$C_{60}H_{85}N_{15}O_{14}$, 1239.64; found, 1241.89 $[M + H]^+$; HR-ESMS: *m/z* calculated for $C_{60}H_{85}N_{15}O_{14}$, 1239.63949; found 1239.63906.

RZ6

The product was obtained as white powder in overall yield 0.2%.

Characterization by analytical HPLC (linear gradient from 20:80 to 80:20 (0.036% TFA in ACN/0.045% TFA in H_2O) in 8 min; t_R = 4.3 min); HPLC-ESMS (gradient from 30:70 to 100:0 (0.07% HCO_2H in ACN/0.1% HCO_2H in H_2O) in 8 min; t_R = 3.3 min; *m/z* calculated for $C_{56}H_{77}N_{15}O_{14}$, 1183.58; found, 1185.01 $[M + H]^+$; HR-ESMS: *m/z* calculated for $C_{56}H_{77}N_{15}O_{14}$, 1183.57689; found 1183.57278.

RZ7

The product was obtained as white powder in overall yield 3%.

Characterization by analytical HPLC (linear gradient from 20:80 to 80:20 (0.036% TFA in ACN/0.045% TFA in H_2O) in 8 min; t_R = 5.2 min); HPLC-ESMS (gradient from 30:70 to 100:0 (0.07% HCO_2H in ACN/0.1% HCO_2H in H_2O) in 8 min; t_R = 4.6 min; *m/z* calculated for $C_{60}H_{85}N_{15}O_{14}$, 1239.64; found, 1241.16 $[M + H]^+$; HR-ESMS: *m/z* calculated for $C_{60}H_{85}N_{15}O_{14}$, 1239.63949; found 1239.63764.

RZ8

The product was obtained as yellow powder in overall yield 5.6%.

Characterization by analytical HPLC (linear gradient from 30:70 to 100:0 (0.036% TFA in ACN/0.045% TFA in H_2O) in 8 min; t_R = 3.1 min); HPLC-ESMS (gradient from 30:70 to 100:0 (0.07% HCO_2H in ACN/0.1% HCO_2H in H_2O) in 8 min; t_R = 3.0 min; *m/z* calculated for $C_{56}H_{77}N_{15}O_{14}$, 1183.58; found, 1185.73 $[M + H]^+$; HR-ESMS: *m/z* calculated for $C_{56}H_{77}N_{15}O_{14}$, 1183.57689; found 1183.57781.

RZ9

The product was obtained as white powder in overall yield 4%.

Characterization by analytical HPLC (linear gradient from 30:70 to 100:0 (0.036% TFA in

ACN/0.045% TFA in H_2O) in 8 min; t_R = 5.6 min); HPLC-ESMS (gradient from 30:70 to 100:0 (0.07% HCO_2H in ACN/0.1% HCO_2H in H_2O) in 8 min; t_R = 7.8 min; *m/z* calculated for $C_{64}H_{93}N_{15}O_{12}$, 1263.71; found, 1265.94 $[M + H]^+$; HR-ESMS: *m/z* calculated for $C_{64}H_{93}N_{15}O_{12}$, 1263.71227; found 1263.71240.

RZ10

The product was obtained as white powder in overall yield 7%.

Characterization by analytical HPLC (linear gradient from 30:70 to 100:0 (0.036% TFA in ACN/0.045% TFA in H_2O) in 8 min; t_R = 5.4 min); HPLC-ESMS (gradient from 30:70 to 100:0 (0.07% HCO_2H in ACN/0.1% HCO_2H in H_2O) in 8 min; t_R = 5.9 min; *m/z* calculated for $C_{60}H_{85}N_{15}O_{12}$, 1207.65; found, 1209.85 $[M + H]^+$; HR-ESMS: *m/z* calculated for $C_{60}H_{85}N_{15}O_{12}$, 1207.64967; found 1207.65062.

RZ11

The product was obtained as white powder in overall yield 0.7%.

Characterization by analytical HPLC (linear gradient from 30:70 to 100:0 (0.036% TFA in ACN/0.045% TFA in H_2O) in 8 min; t_R = 5.6 min); HPLC-ESMS (gradient from 30:70 to 100:0 (0.07% HCO_2H in ACN/0.1% HCO_2H in H_2O) in 8 min; t_R = 6.7 min; *m/z* calculated for $C_{64}H_{93}N_{15}O_{12}$, 1263.71; found, 1266.02 $[M + H]^+$; HR-ESMS: *m/z* calculated for $C_{64}H_{93}N_{15}O_{12}$, 1263.71227; found 1263.71376.

RZ12

The product was obtained as white powder in overall yield 0.2%.

Characterization by analytical HPLC (linear gradient from 30:70 to 100:0 (0.036% TFA in ACN/0.045% TFA in H_2O) in 8 min; t_R = 5.1 min); HPLC-ESMS (gradient from 30:70 to 100:0 (0.07% HCO_2H in ACN/0.1% HCO_2H in H_2O) in 8 min; t_R = 6.1 min; *m/z* calculated for $C_{60}H_{85}N_{15}O_{12}$, 1207.65; found, 1209.70 $[M + H]^+$; HR-ESMS: *m/z* calculated for $C_{60}H_{85}N_{15}O_{12}$, 1207.64967; found 1207.65076.

RZ2CF

The product was obtained as yellow powder in overall yield 5.3%.

Characterization by analytical HPLC (linear gradient from 30:70 to 100:0 (0.036% TFA in ACN/0.045% TFA in H_2O) in 8 min; t_R = 4.8 min); HPLC-ESMS (gradient from 30:70 to 100:0 (0.07% HCO_2H in ACN/0.1% HCO_2H in H_2O) in 8 min; t_R = 5.1 min; *m/z* calculated for $C_{79}H_{92}N_{16}O_{17}$, 1536,68264; found, 1538,67 $[M + H]^+$; HR-ESMS: *m/z* calculated for $C_{79}H_{92}N_{16}O_{17}$, 1536,68264; found 1537.6899.

Biological assays

General

Human serum, cathepsin B from human liver, Sephadex® G-25, propidium iodide, bafilomycin (Baf) were obtained from Sigma-Aldrich (St. Louis, USA). Recombinant Human Matrix Metalloproteinase 2 (MMP-2) was obtained from Sina Biological Inc. (Beijing, China). Egg L-α-phosphatidylcholine (EPC) and cholesterol (Chol) were obtained from Avanti Polar Lipids (Alabaster, AL).

Antibodies

Antibodies for caspase-3 (1:700), PARP (1:1000), Atg5 (1:1000), and porin (1:2000) are from Cell Signaling Technology (Danvers, MA USA), p62 (1:2000) from BD Biosciences (Franklin Lakes, NJ USA), LC3 (1:2000) from MBL International Corporation (Woburn, MA USA), and actin (1:15000) from Sigma (St. Louis, USA).

Cells and cell culture

Cell culture media and FBS were obtained from Life Technologies Corporation (California, USA) and Thiazolyl Blue Tetrazolium Bromide (MTT) from Sigma-Aldrich (St. Louis, USA). The four human cell lines were obtained from the American Type Culture Collection (ATCC). The HeLa cervical adenocarcinoma cells were grown in DMEM, A-549 lung carcinoma cells in F-12K Medium and SK-BR-3 breast adenocarcinoma and HT-29 colon adenocarcinoma cells in McCoy's 5a Medium Modified, all of them supplemented with 10% fetal bovine serum (FBS), 2 mM L-glutamine and antibiotics. Cells were sub-cultured twice a week and maintained at 37 ºC in a humidified atmosphere containing 5% CO_2. For sub-culturing, cells were detached from culture flasks by incubation with 0.25% trypsin-EDTA 3 min (at 37 ºC, 5% CO_2). Trypsinated cells were centrifugated at 1000 rpm for 10 min at room temperature and gently resuspended in 5 mL of fresh medium preheated at 37 ºC. Cellular density was determined in a Neubauer counting plate and the appropriate volume of cells was placed into a fresh culture flask or a 96-well plate. After 24 h incubation, cells were completely attached to the surface.

Western blotting assays

Cells were homogenized in RIPA (150 mM NaCl, 10 mM Tris, pH 7.2, 0.1% SDS, 1% Triton X-100, 1% deoxycholate, 5 mM EDTA, 1 mM $NaVO_4$, 5 mM NaF, 1 mM PMSF, and protease inhibitor mixture (Roche)) and centrifuged at 10000 g for 15 min at 4 ºC. Proteins from total homogenates were resolved in 12% acrylamide gels for SDS-PAGE and transferred to Immobilon membranes (Millipore). β-actin was used as a loading control.

Confocal microscopy

For live imaging studies, HeLa cells were plated on 8-well Lab-Teck chambered 1.0 borosilicate coverglass system (Nalge Nunc International, Rochester, NY). Cells were placed in a chamber under culture conditions (DMEM at 37 ºC and 5% CO_2), and live cells were visualized using a Leica SP2 Confocal Microscope. To prevent crosstalk, emission signals were recorded sequentially. Images were then processed with ImageJ software (NIH).

Stability assays

For human serum degradation, RZ2 was incubated directly with the serum. For cathepsin B cleavages, 3 μL of a dilution (1/10) of enzyme stock solution (≥ 2000 units/mg) were added to the activation buffer (30 mM DTT, 15 mM EDTA). After 15 min at RT, reaction buffer (1 mM EDTA, 25 mM acetate buffer pH 5.0) and RZ2 solution were added to previously activated cathepsin B solution. For MMP-2 cleavages, 2 μL of enzyme solution (25 mg/ml) were added directly to the reaction buffer (10 mM $CaCl_2$, 100mM NaCl, 50 mM Tris pH 7.5), and RZ2 was then added. For the three digestions, the final peptide concentration was 40 μM, except for the human serum that was 100 μM, and the final solution was incubated at 37 ºC. Aliquots from cathepsin B and MMP-2 samples were removed at several time points and were immediately frozen in liquid nitrogen and stored. Each sample was defrosted individually, and the compound was rapidly analyzed quantitatively by RP-HPLC. For human serum samples, an aliquot of 50 μL was mixed with 250 μL of cold ethanol, after 30 min of centrifugation (13,000 rpm) the supernatant was evaporated and, finally, each dry sample was re-dissolved with 5 μL of DMSO and, afterwards, 95 μL of water were added. In the end, the samples were analyzed by RP-HPLC.

Leakage measurement

Aliquots containing the appropriate amount of lipid in chloroform/methanol (1:1, v/v) were placed in a test tube, the solvents were removed by evaporation under a stream of O_2-free nitrogen, and finally traces of solvents were eliminated under vacuum in the dark for more than 3 h. After that, 1 mL of buffer containing 10 mM HEPES, 100 mM NaCl, 0.1 mM EDTA, pH 7.4 buffer and CF at a concentration of 40mM was added, and multilamellar vesicles were obtained. Large unilamellar vesicles (LUVs) with a mean diameter of 200 nm were prepared from multilamellar vesicles by the LiposoFast device from Avestin, Inc., using polycarbonate filters with a pore size of 0.2 μm (Nuclepore Corp., Cambridge, CA, USA). Breakdown of the vesicle membrane leads to content leakage, i. e., CF fluorescence. Non-encapsulated CF was separated from the vesicle suspension through a Sephadex G-25 filtration column eluted with buffer containing 10 mM HEPES, 150 mM NaCl, and 0.1 mM EDTA, pH 7.4. Leakage of intraliposomal CF was assayed by treating the probe-loaded liposomes (final lipid concentration, 0.125 mM) with the appropriate amount of RZ2 or melittin in Costar 3797 round-bottom 96-well plates, each well containing a final volume of 100 μL. The micro titer plate was incubated at 37 ºC for 1 h to induce dye leakage. Leakage was measured at various compound-to-lipid ratios. Changes in fluorescence intensity were recorded using the FL600 fluorescence microplate reader with excitation and emission wavelengths set at 492 and 517 nm, respectively. One hundred percent release was achieved by adding Triton X-100 to a final concentration of 1% v/v to the microtiter plates. Fluorescence measurements were made initially with probe-loaded liposomes, afterwards by adding RZ2 solution and, eventually, adding Triton X-100 to obtain 100% leakage. The results were expressed as percentage of CF released relative to the positive control (Triton X-100).

Red blood cells lysis assay

Human blood was collected in 10 mL EDTA Vacutainer tubes. A small aliquot was assessed for evidence of hemolysis by centrifugation at 800 g for 10 min, and non-hemolyzed samples were carried forward into the assay. Red blood cells (RBCs) were washed three times in PBS pH 7.4 by centrifuging at 800 g for 10 min and resuspending in the same buffer to yield a 10x dilution. RBCs were then diluted in appropriate pH buffer to yield approximately $\pm 15 \times 10^7$ cells/100 μL

PBS in Costar 3797 round-bottom 96-well plates for the lysis assay. The micro titer plate was covered with a low evaporation lid and incubated in a 37 ºC warm room for 1 h to induce hemolysis. Negative controls were PBS, while positive controls were 1% v/v solution of Triton X-100 (100% lysis). The plate was then centrifuged at 800 g for 10 min and 80 μL of supernatants were transferred to a Costar 3632 clear bottom 96-well plate. Hemoglobin's absorbance was read at 560 nm using the ELx800 absorbance microplate reader. The results were expressed as percentage of hemoglobin released relative to the positive control (Triton X-100).

Flow cytometric analysis of apoptosis and necrosis

Extend of apoptosis was measured through Alexa Fluor® 488 annexin V/Dead Cell Apoptosis Kit (Invitrogen, USA) as described by the manufacturer's instructions. Briefly, HeLa cells were harvested at the indicated times after treatment. Culture medium supernatant and PBS washes were retained to ensure that both floating and adherent cells were analyzed. After incubation for 15 min with Alexa Fluor® 488 annexin V and PI working solution, cells were subjected to FACS analysis.

Cell apoptosis assays

DNA fragmentation was determined in HeLa cells previously permeabilized with ethanol and labeled with propidium iodide (PI). The sub-G_1 population was quantified by flow cytometry. DEVDase activity was determined in 30 μg of RIPA total protein cell lysate (without protease inhibitors). Cleavage of the fluorogenic substrated Ac-DEVD-AFC. Caspase 3 and PARP cleavage were determined in 50 μg RIPA total protein cell lysate of control and treated cells.

Flow cytometric analysis of cellular DNA content

Flow cytometric analysis of cellular DNA content was performed as described in Current Protocols in Cytometry.[2] Both floating and attached cells were collected and poured together in the centrifuge tubes. Cells were washed with phosphate-buffered saline (PBS), re-suspended and fixed in 70% ice-cold ethanol for 4 h at 4°C. Subsequently, they were treated with a IP/Triton X-100/RNase A for 30 min. Finally, cells were analyzed in a Coulter XL flow cytometer. At least 10,000 events per sample were analyzed three times. The percentage of cells in G_0/G_1

phase, S phase and G_2/M phase was analyzed using the Multicycle software.

Autophagy flux analysis

HeLa cells were incubated in the presence or absence of 100 nM bafilomycin for 4 h. After that time, they were processed for immunodetection of LC3. LC3-II abundance was normalized by β-actin levels.

Labeling of acidic compartment

HeLa cells were loaded with 100 nM Lysotracker Green (Molecular Probes, Invitrogene) for the last 20 min of incubation. Cells were then analyzed by flow cytometry.

Mitochondrial membrane potential assay (TMRE assay)

TMRE membrane potential kit from Abcam (Cambridge, MA) was used according to manufacturer's instructions. TMRE was added to the media at 50 nM final concentration and cells were incubated for 20 min at 37 °C, 5% CO_2. After incubation, cells were trypsinized, centrifuged, and cell pellets were resuspended in 0.4 mL of medium and analyzed by flow cytometry. The excitation/emission fluorescence for TMRE is 549/575 nm.

Determination of mitochondrial superoxide

MitoSOX red mitochondrial superoxide indicator (Invitrogen, San Diego, CA) was used to detect superoxide, as a general measure of cellular oxidative stress in the mitochondria of live cells.
The red fluorescence was analyzed by flow cytometry (excitation/emission fluorescence is 510/580 nm).

Microarray data analysis

HeLa cells treated with 5 μM RZ2 or just the vehicle for 24 h are washed with PBS and then lysed in 0.35 mL lysis buffer containing β-mercaptoethanol. Isolation steps are done following PureLink® RNA Mini Kit technical manual. The optional step of DNAse treatment is performed.

RNA quantification is done using a ND-1000 spectrophotometer using the elution buffer as blank. RNA integrity control was done using RNA Nanochips 6000 on Agilent's Bioanalyzer 2100 according to the technical manual. RNA expression profiling was performed following the Pico Profiling method. Briefly, cDNA library preparation and amplification were performed from 25 ng total RNA using WTA2 (Sigma-Aldrich) with 17 cycles of amplification. 8 μg cDNA were subsequently fragmented by DNase I and biotinylated by terminal transferase obtained from GeneChip Mapping 250K Nsp Assay Kit (Affymetrix). Hybridization mixture was prepared according to Affymetrix protocol. Each sample was hybridized to a GeneChip PrimeView Human Gene Expression Array (Affymetrix). Arrays were washed and stained in a Fluidics Station 450 and scanned in a GeneChip Scanner 3000 (both Affymetrix) according to manufacturer's recommendations. CEL files were generated from DAT files using GCOS software (Affymetrix). Arrays were processed in Bioconductor, using RMA background correction and summarization. Foldchanges between samples were computed after MA mean and variance normalization using the GAM method. An empirical Bayes partial density model was then used to identify significant differentially expressed genes with a False Discovery Rate (FDR) of 5% and a log2 foldchange threshold of 3 (8 times up or down regulated). The whole list of genes in the array (ranked by mean foldchange from most upregulated to most downregulated) was analyzed against Human GO Biological Process and KEGG databases in order to detect overrepresented genesets with a GSEA pre-ranked analysis.

SUPPLEMENTARY REFERENCES

(1) Biron, E.; Chatterjee, J.; Kessler, H., *J. Pept. Sci.* 2006, **12**, 213–219.

(2) Darzynkiewicz, Z.; Juan, G., *Curr. Protoc. Cytom.* 2001, *Chapter 7*, Unit 7.5.
