# Supplementary material for: A new quinoxaline-containing peptide induces apoptosis in cancer cells by autophagy modulation
Source: Chem Sci. 2015 May 20;6(8):4537–49. doi: 10.1039/c5sc00125k (PMC5666514; doi:10.1039/c5sc00125k)
Supplement: Supplementary file 3 [file SC-006-C5SC00125K-s003.pdf]

Electronic Supplementary Material (ESI) for Chemical Science.
This journal is © The Royal Society of Chemistry 2015

Upregulated						
symbol	gene name	5 µM RZ2	control	log2FC	FC	probDE
ESRRG	estrogen-related receptor gamma	4,46209702	2,57077924	10,1361026	1125,30671	1
DMKN	dermokine	12,5322674	6,05968172	7,65595976	201,684969	1
CRABP2	cellular retinoic acid binding protein 2	12,9003181	8,45937398	7,46754037	176,992004	1
LDB3	LIM domain binding 3	4,02678175	2,7495672	7,32876962	160,760552	0,99999672
IFITM1	interferon induced transmembrane protein 1 (9-27)	12,1007125	5,56000433	7,1273187	139,809512	1
IFITM1	interferon induced transmembrane protein 1 (9-27)	12,1266679	6,02077056	7,09356357	136,576321	1
ASS1	argininosuccinate synthase 1	12,3843485	6,77851338	6,90107713	119,517422	1
FZD3	frizzled homolog 3 (Drosophila)	4,37047668	2,8701083	6,88523536	118,212219	0,99999999
BMP5	bone morphogenetic protein 5	11,9629178	4,22424277	6,84844102	115,235468	1
ASS1	argininosuccinate synthase 1	12,5750825	7,24611949	6,83490304	114,159178	1
RIMS1	regulating synaptic membrane exocytosis 1	10,5653003	3,42535792	6,6650756	101,481687	1
APOBEC3C	apolipoprotein B mRNA editing enzyme, catalytic polypeptide-like 3C	11,8749958	6,20801573	6,63937033	99,689547	1
RIMS1	regulating synaptic membrane exocytosis 1	9,70947755	3,24299347	6,55456769	93,9986211	1
CRABP2	cellular retinoic acid binding protein 2	12,5477972	7,99862219	6,54828176	93,5899531	1
APOBEC3C	apolipoprotein B mRNA editing enzyme, catalytic polypeptide-like 3C	11,9851385	6,70329215	6,4287659	86,1492241	1
CKMT1B	creatine kinase, mitochondrial 1B	8,43768912	3,21942034	6,41548895	85,3600404	1
LGALS3BP	lectin, galactoside-binding, soluble, 3 binding protein	12,4932661	8,27548192	6,39542704	84,1812506	1
KRT81	keratin 81	12,0693542	6,86904419	6,39299679	84,0395652	1
RIMS1	regulating synaptic membrane exocytosis 1	10,3046225	3,65757522	6,20256385	73,6474594	1
IL32	interleukin 32	11,4795967	5,18874756	6,11363612	69,2449098	1
IL32	interleukin 32	11,4815953	5,07633641	6,10303122	68,7377735	1
C4BPB	complement component 4 binding protein, beta	11,4518335	6,03048428	6,08480523	67,8748516	1
MAGEA12	melanoma antigen family A, 12	7,51961365	3,24746802	6,07657128	67,4885702	1
RIMS1	regulating synaptic membrane exocytosis 1	10,4463831	3,89633797	6,07220615	67,2846798	1
IL32	interleukin 32	11,3619191	4,63508682	6,041123	65,8505234	1
NUP210	nucleoporin 210kDa	11,3947484	5,33180055	6,01151327	64,5127884	1
IL32	interleukin 32	11,377455	4,88111054	5,97577961	62,934518	1
DMKN	dermokine	11,6220651	6,73252395	5,91313657	60,260326	1

DMKN	dermokine	11,2184946	5,3067708	5,75793525	54,1141975	1
CRABP2	cellular retinoic acid binding protein 2	11,8638492	7,2803291	5,73521429	53,2686307	1
PLAC8	placenta-specific 8	12,2542615	8,79008347	5,70394188	52,1263842	1
SCARA5	scavenger receptor class A, member 5 (putative)	11,1606642	5,63331113	5,68662431	51,5044189	1
SRGN	serglycin	9,7014722	3,69881503	5,68426272	51,4201786	1
ELF3	E74-like factor 3 (ets domain transcription factor, epithelial-specific)	10,6296322	4,37930991	5,66329482	50,6782505	1
PSMB9	proteasome (prosome, macropain) subunit, beta type, 9 (large multifunctio	11,3895704	6,71268767	5,61166274	48,8966167	1
VAV3	vav 3 guanine nucleotide exchange factor	10,9083345	4,71121362	5,5649166	47,3376637	1
ELF3	E74-like factor 3 (ets domain transcription factor, epithelial-specific)	10,6403073	4,48194447	5,55682507	47,0729077	1
DMKN	dermokine	9,8898777	4,05907235	5,44960926	43,7014505	1
MB	myoglobin	10,2640704	4,33736633	5,43902874	43,3821227	1
ZNF493	zinc finger protein 493	3,7090674	2,83935271	5,43179858	43,1652542	0,99351427
MB	myoglobin	10,1959413	4,30190604	5,4280962	43,0546215	1
BCL6B	B-cell CLL/lymphoma 6, member B	7,91710728	3,4790562	5,4053816	42,382054	1
EPGN	epithelial mitogen homolog (mouse)	8,95983528	3,59493234	5,39899413	42,1948239	1
RIMS1	regulating synaptic membrane exocytosis 1	10,255485	4,37470974	5,38937899	41,914543	1
DMKN	dermokine	11,637212	7,31964208	5,38270577	41,7211139	1
DMKN	dermokine	10,7168306	4,75162346	5,36204872	41,1279916	1
RASGEF1A	RasGEF domain family, member 1A	8,72546924	3,57888404	5,33815475	40,452438	1
PLAC8	placenta-specific 8	9,52140202	3,94768085	5,25687438	38,2363892	1
DMKN	dermokine	10,6706591	4,83573813	5,25621133	38,2188199	1
AGXT2L1	alanine-glyoxylate aminotransferase 2-like 1	4,30816112	3,05656461	5,24447917	37,9092805	0,99999401
VAV3	vav 3 guanine nucleotide exchange factor	10,8677829	5,90457333	5,22725575	37,4593965	1
METTL7A	methyltransferase like 7A	8,18002113	3,55752548	5,22715809	37,4568607	1
RIMS1	regulating synaptic membrane exocytosis 1	9,80878191	4,21438744	5,21949825	37,2585145	1
INSL4	insulin-like 4 (placenta)	8,49574566	3,59392989	5,21836139	37,229166	1
ANK3	ankyrin 3, node of Ranvier (ankyrin G)	6,27401352	3,29925856	5,16498698	35,8769906	1
RIMS1	regulating synaptic membrane exocytosis 1	7,98508967	3,56719792	5,14680376	35,4276474	1
SLC12A3	solute carrier family 12 (sodium/chloride transporters), member 3	10,0246706	4,44729516	5,13042507	35,0277174	1
PLAC8	placenta-specific 8	11,9221321	8,30578116	5,12770693	34,9617849	1
DNAH2	dynein, axonemal, heavy chain 2	4,69033221	3,13601771	5,12518565	34,9007383	1

DMKN	dermokine	11,6135907	7,58416511	5,10269477	34,3608727	1
MUC13	mucin 13, cell surface associated	10,7106039	5,29883912	5,08674111	33,9829953	1
C3	complement component 3	10,5500098	4,96402218	5,05325789	33,2033728	1
METTL7A	methyltransferase like 7A	10,7286753	5,89815129	5,02202184	32,492207	1
SPRY4	sprouty homolog 4 (Drosophila)	10,9409644	6,68047531	5,0083047	32,1847354	1
NUP210	nucleoporin 210kDa	10,7878158	6,34210084	4,97952547	31,5490677	1
TMPRSS3	transmembrane protease, serine 3	3,96072381	2,99476979	4,95789735	31,0796285	0,99849391
SERPINF1	serpin peptidase inhibitor, clade F (alpha-2 antiplasmin, pigment epithelium	4,62856968	3,15941964	4,9140823	30,1499207	0,99999997
KLRC3	killer cell lectin-like receptor subfamily C, member 3	5,89815129	3,30325683	4,88904113	29,6311174	1
LEF1	lymphoid enhancer-binding factor 1	6,8532053	3,51494407	4,8766764	29,3782469	1
MCF2L	MCF.2 cell line derived transforming sequence-like	10,280984	4,91789145	4,83953945	28,6316608	1
ZNF883	zinc finger protein 883	7,516045	3,62690502	4,82912379	28,4256964	1
VAV3	vav 3 guanine nucleotide exchange factor	9,55304627	4,41369444	4,79576017	27,7758694	1
KIF21B	kinesin family member 21B	5,95111172	3,34008615	4,78817759	27,6302667	1
DMKN	dermokine	10,0252854	4,76551552	4,77512566	27,3814257	1
MAGEA1	melanoma antigen family A, 1 (directs expression of antigen MZ2-E)	8,73189505	3,86981717	4,75881538	27,0736103	1
FBXO2	F-box protein 2	10,6622538	6,43407871	4,75177556	26,9418228	1
ANK3	ankyrin 3, node of Ranvier (ankyrin G)	6,41593915	3,4593718	4,75104599	26,9282019	1
EPGN	epithelial mitogen homolog (mouse)	5,22108033	3,25381038	4,72401758	26,4284074	1
HNF4A	hepatocyte nuclear factor 4, alpha	9,46329279	4,42737332	4,70741228	26,1259624	1
CSAG1	chondrosarcoma associated gene 1	10,639246	6,51705554	4,67525688	25,5500973	1
DCP2	DCP2 decapping enzyme homolog (S. cerevisiae)	3,28362786	2,69595763	4,67352985	25,51953	0,82436376
APOBEC3C	apolipoprotein B mRNA editing enzyme, catalytic polypeptide-like 3C	10,5611797	6,28513674	4,66774792	25,4174593	1
SCARA5	scavenger receptor class A, member 5 (putative)	10,0937943	4,94726728	4,65096243	25,1234455	1
AIF1L	allograft inflammatory factor 1-like	11,7387889	8,58661394	4,60649546	24,3608988	1
JAG2	jagged 2	12,4617061	10,6103203	4,60310971	24,3037952	1
EPCAM	epithelial cell adhesion molecule	10,5475424	6,39679474	4,60082128	24,2652746	1
PLAC8	placenta-specific 8	11,3124715	7,67179829	4,59007569	24,0852116	1
COL5A2	collagen, type V, alpha 2	9,11886996	4,2667382	4,58359797	23,977311	1
CDH26	cadherin 26	3,68039066	2,92487132	4,57184199	23,7827228	0,96945357
LMF1	lipase maturation factor 1	6,5514951	3,56130006	4,5468191	23,3737791	1

ODZ3	odz, odd Oz/ten-m homolog 3 (Drosophila)	7,34524425	3,7090674	4,5285373	23,0794558	1
NLRP1	NLR family, pyrin domain containing 1	8,98550149	4,21757086	4,51977106	22,9396434	1
CITED4	Cbp/p300-interacting transactivator, with Glu/Asp-rich carboxy-terminal do	10,434967	6,18977042	4,51638337	22,8858405	1
GNG4	guanine nucleotide binding protein (G protein), gamma 4	10,533873	6,54117351	4,50857771	22,7623517	1
VAV3	vav 3 guanine nucleotide exchange factor	8,94265484	4,19407087	4,50801857	22,7535314	1
COL5A2	collagen, type V, alpha 2	10,3462432	5,78559123	4,50005037	22,6282069	1
NLRP1	NLR family, pyrin domain containing 1	8,61894848	3,99585941	4,49126667	22,4908561	1
DMKN	dermokine	10,2619617	5,48661999	4,48059238	22,3250636	1
PZP	pregnancy-zone protein	7,09168044	3,69286165	4,47184295	22,1900798	1
METTL7A	methyltransferase like 7A	10,1943154	5,34330739	4,46613718	22,1024926	1
IGFBP2	insulin-like growth factor binding protein 2, 36kDa	10,1513255	5,27591658	4,45736009	21,9684334	1
MYSM1	Myb-like, SWIRM and MPN domains 1	5,29415187	3,31592942	4,45604887	21,948476	1
PCSK6	proprotein convertase subtilisin/kexin type 6	6,06706141	3,47069254	4,44102785	21,721139	1
SHISA2	shisa homolog 2 (Xenopus laevis)	5,22285753	3,31236712	4,43007205	21,5568138	1
ANK3	ankyrin 3, node of Ranvier (ankyrin G)	6,60181409	3,63703787	4,36353052	20,585128	1
MMP7	matrix metallopeptidase 7 (matrilysin, uterine)	5,48921601	3,36560189	4,36332014	20,5821265	1
COL6A1	collagen, type VI, alpha 1	8,95309664	4,35299337	4,35140642	20,41286	1
AQP3	aquaporin 3 (Gill blood group)	11,7634995	9,10732838	4,34960643	20,3874076	1
NSMCE4A	non-SMC element 4 homolog A (S. cerevisiae)	3,41974981	2,81125665	4,34749751	20,3576271	0,85452785
SLC4A4	solute carrier family 4, sodium bicarbonate cotransporter, member 4	5,47057691	3,36687211	4,3452078	20,3253431	1
PZP	pregnancy-zone protein	6,84104314	3,69834327	4,33309273	20,1553752	1
MUC13	mucin 13, cell surface associated	9,39913102	4,73266803	4,3192729	19,963225	1
PSMB9	proteasome (prosome, macropain) subunit, beta type, 9 (large multifunctio	9,07182884	4,48386558	4,31876373	19,9561807	1
USH1C	Usher syndrome 1C (autosomal recessive, severe)	8,85311587	4,32260837	4,29590612	19,6424927	1
STRA6	stimulated by retinoic acid gene 6 homolog (mouse)	10,2255781	5,94234501	4,29185705	19,5874413	1
NID2	nidogen 2 (osteonidogen)	10,2968647	6,26622575	4,288006	19,5352255	1
CCND2	cyclin D2	4,61989473	3,26321696	4,25347204	19,0731608	0,99999951
COL9A3	collagen, type IX, alpha 3	10,0729853	5,51085848	4,24770789	18,9971079	1
AQP3	aquaporin 3 (Gill blood group)	11,7339206	9,15582509	4,24762907	18,9960699	1
BIRC7	baculoviral IAP repeat containing 7	10,4575211	6,82828478	4,21680093	18,5944598	1
TNNC1	troponin C type 1 (slow)	11,3368617	8,11413405	4,21652118	18,5908545	1

CD36	CD36 molecule (thrombospondin receptor)	4,94726728	3,32410795	4,19153679	18,2716724	1
ALDOC	aldolase C, fructose-bisphosphate	9,42716945	4,87383125	4,18497623	18,1887718	1
NRCAM	neuronal cell adhesion molecule	7,0495635	3,78915858	4,1825238	18,1578792	1
SCGB3A2	secretoglobin, family 3A, member 2	8,91870734	4,48879856	4,18167666	18,1472201	1
CARD10	caspase recruitment domain family, member 10	10,4674877	6,91407069	4,16702558	17,9638613	1
COL4A6	collagen, type IV, alpha 6	10,676276	7,251593	4,15928099	17,8676871	1
LOC644794	hypothetical LOC644794	4,79019217	3,31233651	4,13181172	17,5307003	0,99999998
C1orf106	chromosome 1 open reading frame 106	7,17390322	3,8297844	4,12665182	17,4681125	1
STC1	stanniocalcin 1	7,21807089	3,8387456	4,12125009	17,4028308	1
ABCG1	ATP-binding cassette, sub-family G (WHITE), member 1	5,91035836	3,53449653	4,11211789	17,2930195	1
KLRC3	killer cell lectin-like receptor subfamily C, member 3	4,58989291	3,28438123	4,09777583	17,1219585	0,99999832
FA2H	fatty acid 2-hydroxylase	8,39433061	4,16445253	4,0949303	17,088221	1
PLEKHA6	pleckstrin homology domain containing, family A member 6	9,33430335	4,94012221	4,03820303	16,4293446	1
ALDOC	aldolase C, fructose-bisphosphate	10,112551	6,25481097	4,02769795	16,3101477	1
SALL4	sal-like 4 (Drosophila)	10,2098167	6,60074946	4,00623087	16,069252	1
DMKN	dermokine	10,0743244	6,16987223	4,00599449	16,0666194	1
ROBO2	roundabout, axon guidance receptor, homolog 2 (Drosophila)	4,00880837	3,14061541	4,00426331	16,0473516	0,9933761
EPCAM	epithelial cell adhesion molecule	9,15532394	4,84188354	4,00226528	16,0251425	1
STRA6	stimulated by retinoic acid gene 6 homolog (mouse)	10,0447491	6,08433421	3,99596919	15,9553593	1
SORT1	sortilin 1	9,80837328	5,45707356	3,98742806	15,8611784	1
VWA5A	von Willebrand factor A domain containing 5A	6,32206327	3,70293937	3,98182286	15,7996738	1
PRG4	proteoglycan 4	4,41287418	3,26686801	3,98026064	15,7825744	0,99994095
HNF4A	hepatocyte nuclear factor 4, alpha	5,97803275	3,59966033	3,97995531	15,7792345	1
EPCAM	epithelial cell adhesion molecule	8,54459951	4,37084909	3,97750544	15,7524621	1
MB	myoglobin	10,105755	6,36933804	3,97061854	15,6774449	1
PLA2G3	phospholipase A2, group III	8,36879425	4,2667382	3,94682072	15,4209605	1
C8orf73	chromosome 8 open reading frame 73	10,1345612	6,52149718	3,94006358	15,3489024	1
COL16A1	collagen, type XVI, alpha 1	10,9745643	7,86235123	3,92750986	15,2159222	1
CPS1	carbamoyl-phosphate synthase 1, mitochondrial	12,5222576	11,0634743	3,91375702	15,0715618	0,99999996
HYAL1	hyaluronoglucosaminidase 1	9,65283544	5,37041322	3,89438542	14,870543	1
HYAL1	hyaluronoglucosaminidase 1	9,87746737	5,78154731	3,89338671	14,8602524	1

NAA11	N(alpha)-acetyltransferase 11, NatA catalytic subunit	8,35880346	4,30730444	3,89064677	14,8320568	1
ESRRG	estrogen-related receptor gamma	3,43997477	2,8746907	3,88844446	14,8094326	0,78537139
VAV3	vav 3 guanine nucleotide exchange factor	9,86742766	5,81926977	3,86457124	14,5663876	1
C12orf59	chromosome 12 open reading frame 59	7,910665	4,1009461	3,85862674	14,5064916	1
GDF6	growth differentiation factor 6	5,10997027	3,42665727	3,82836956	14,2054197	1
PYGM	phosphorylase, glycogen, muscle	6,66785995	3,84871203	3,82617211	14,1837992	1
ALDOC	aldolase C, fructose-bisphosphate	9,76051822	5,64650068	3,82258866	14,1486123	1
UCP2	uncoupling protein 2 (mitochondrial, proton carrier)	12,1468622	10,5194559	3,82204721	14,1433033	1
KRT18	keratin 18	12,1512162	10,5289423	3,81880743	14,1115782	1
CDK18	cyclin-dependent kinase 18	8,82367818	4,75941913	3,81621399	14,0862334	1
KLHL4	kelch-like 4 (Drosophila)	3,70940462	3,03399433	3,81477073	14,0721488	0,92279609
DISP1	dispatched homolog 1 (Drosophila)	3,67346255	3,01604514	3,81009682	14,0266328	0,90792338
NRCAM	neuronal cell adhesion molecule	7,43905106	4,00612526	3,80274924	13,9553774	1
PLEKHA6	pleckstrin homology domain containing, family A member 6	5,93986721	3,65142857	3,79977534	13,9266402	1
OASL	2'-5'-oligoadenylate synthetase-like	11,1711252	8,29697003	3,79329592	13,8642332	1
RPRM	reprimo, TP53 dependent G2 arrest mediator candidate	10,4834087	7,36533105	3,78924208	13,8253307	1
F11R	F11 receptor	9,47675457	5,3044329	3,78794526	13,8129088	1
VWA5A	von Willebrand factor A domain containing 5A	10,777984	7,74898019	3,78087572	13,7453879	1
KCNH2	potassium voltage-gated channel, subfamily H (eag-related), member 2	6,8023412	3,89633797	3,77646688	13,7034465	1
PGF	placental growth factor	9,13012667	5,04728132	3,75715067	13,5211942	1
PRKDC	protein kinase, DNA-activated, catalytic polypeptide	12,1630636	10,6037864	3,72586663	13,2311507	1
ARPP21	cAMP-regulated phosphoprotein, 21kDa	8,66234561	4,71594489	3,72292347	13,2041861	1
IL7	interleukin 7	3,95761926	3,1614709	3,72257466	13,200994	0,98231522
EEF1A2	eukaryotic translation elongation factor 1 alpha 2	11,6256112	9,51001667	3,70829777	13,0710014	1
ABCG1	ATP-binding cassette, sub-family G (WHITE), member 1	6,80866176	3,92463143	3,70717737	13,0608544	1
CLEC2B	C-type lectin domain family 2, member B	8,00832663	4,24831891	3,69230082	12,9268676	1
F11R	F11 receptor	9,50872893	5,45344296	3,69114349	12,9165018	1
KBTBD10	kelch repeat and BTB (POZ) domain containing 10	4,56126343	3,35438967	3,67437546	12,7672461	0,99998333
DDN	dendrin	10,6518169	7,6933128	3,67283869	12,7536535	1
FKBP5	FK506 binding protein 5	10,2553517	7,1709068	3,66068018	12,646622	1
STRA6	stimulated by retinoic acid gene 6 homolog (mouse)	9,61987812	5,69132094	3,64031431	12,4693496	1

T	T, brachyury homolog (mouse)	9,32042571	5,3243553	3,63192839	12,3970795	1
CDH19	cadherin 19, type 2	4,6275231	3,37760987	3,62793255	12,3627908	0,99999377
HYAL1	hyaluronoglucosaminidase 1	9,55057132	5,59674769	3,6269559	12,3544245	1
WISP2	WNT1 inducible signaling pathway protein 2	10,797367	7,92468986	3,61932723	12,2892693	1
EML6	echinoderm microtubule associated protein like 6	6,54084209	3,89915271	3,61864002	12,2834168	1
S100A14	S100 calcium binding protein A14	3,89279446	3,14931614	3,61538478	12,2557323	0,96423442
COL2A1	collagen, type II, alpha 1	9,21989337	5,25314134	3,61528952	12,254923	1
FCGR2A	Fc fragment of IgG, low affinity IIa, receptor (CD32)	7,67868239	4,16597621	3,60502992	12,1680824	1
SLC5A12	solute carrier family 5 (sodium/glucose cotransporter), member 12	6,67062776	3,93786334	3,59813008	12,1100262	1
COL7A1	collagen, type VII, alpha 1	11,7799806	9,98320844	3,58868409	12,0309952	1
F11R	F11 receptor	8,35011952	4,57831998	3,58863139	12,0305558	1
ALDOC	aldolase C, fructose-bisphosphate	9,50135522	5,60358848	3,57155151	11,8889674	1
VWA5A	von Willebrand factor A domain containing 5A	11,0672202	8,36083655	3,57128375	11,886761	1
ESRRG	estrogen-related receptor gamma	4,13612761	3,25321317	3,57067395	11,8817378	0,99461164
ELF3	E74-like factor 3 (ets domain transcription factor, epithelial-specific)	9,29574583	5,36881993	3,56875069	11,8659087	1
UNC5B	unc-5 homolog B (C. elegans)	6,40159674	3,88588508	3,56031809	11,7967545	1
CYP3A5	cytochrome P450, family 3, subfamily A, polypeptide 5	4,17728866	3,26924679	3,55944	11,7895766	0,9962444
KCTD8	potassium channel tetramerisation domain containing 8	3,84623122	3,13748566	3,54994871	11,7122692	0,94573713
PCSK6	proprotein convertase subtilisin/kexin type 6	7,93469453	4,31363218	3,54702512	11,6885585	1
SORBS1	sorbin and SH3 domain containing 1	5,79253037	3,69329627	3,5470093	11,6884304	1
DDN	dendrin	10,1098139	7,06636073	3,5469195	11,6877028	1
DTX4	deltex homolog 4 (Drosophila)	9,63837694	5,9151702	3,53943111	11,6271943	1
COL5A2	collagen, type V, alpha 2	9,37221144	5,48727038	3,53419926	11,5851054	1
COL9A1	collagen, type IX, alpha 1	7,76559612	4,24086139	3,53415736	11,5847689	1
SORT1	sortilin 1	9,4121031	5,5398285	3,52994421	11,5509868	1
PEG10	paternally expressed 10	11,9828397	10,4148151	3,52897068	11,5431949	1
SLC4A4	solute carrier family 4, sodium bicarbonate cotransporter, member 4	3,76759444	3,1026759	3,52571574	11,5171811	0,91440689
F11R	F11 receptor	8,31448201	4,60849893	3,52564326	11,5166024	1
EPGN	epithelial mitogen homolog (mouse)	5,91618007	3,74485966	3,52552131	11,515629	1
DDR2	discoidin domain receptor tyrosine kinase 2	8,34560895	4,65934284	3,50100082	11,3215597	1
APOL6	apolipoprotein L, 6	6,46725549	3,92830206	3,49759688	11,2948788	1

ARL14	ADP-ribosylation factor-like 14	4,582388	3,39523021	3,48564933	11,2017275	0,99997445
MGC39584	hypothetical LOC441058	7,16121224	4,09017956	3,48291905	11,1805484	1
DMKN	dermokine	10,0733204	7,08360357	3,48228281	11,1756188	1
EPPK1	epiplakin 1	9,87592434	6,7016128	3,47189384	11,0954313	1
SLC16A14	solute carrier family 16, member 14 (monocarboxylic acid transporter 14)	7,09239134	4,08050846	3,4708385	11,0873178	1
ADAMTSL4	ADAMTS-like 4	7,23705992	4,11319177	3,47026259	11,0828928	1
GRIN2D	glutamate receptor, ionotropic, N-methyl D-aspartate 2D	9,42147037	5,63475259	3,46785464	11,0644102	1
APOL6	apolipoprotein L, 6	9,26553066	5,45544439	3,46313929	11,028306	1
SPAG9	sperm associated antigen 9	3,70646709	3,08117398	3,45258061	10,9478875	0,8753223
BIRC7	baculoviral IAP repeat containing 7	10,4575211	7,66085332	3,45085691	10,934815	1
ZNF333	zinc finger protein 333	8,41300221	4,77879907	3,43836765	10,840562	1
UCP2	uncoupling protein 2 (mitochondrial, proton carrier)	12,5000716	11,250249	3,43288758	10,7994623	0,99999375
NRCAM	neuronal cell adhesion molecule	7,44200362	4,1852606	3,43280847	10,7988702	1
CEBPA	CCAAT/enhancer binding protein (C/EBP), alpha	8,218687	4,61735699	3,43108145	10,7859508	1
RNF125	ring finger protein 125	6,01094115	3,81589546	3,42804461	10,7632705	1
COL4A6	collagen, type IV, alpha 6	9,61524443	6,09564551	3,4223856	10,7211339	1
TGFA	transforming growth factor, alpha	8,96123273	5,23477618	3,4187649	10,6942611	1
ACBD7	acyl-CoA binding domain containing 7	7,98849863	4,45274151	3,41292061	10,6510268	1
ST6GALNAC1	ST6 (alpha-N-acetyl-neuraminyl-2,3-beta-galactosyl-1,3)-N-acetylgalactosam	4,73753483	3,44724524	3,40324506	10,5798338	0,9999976
KCNMB2	potassium large conductance calcium-activated channel, subfamily M, beta	3,58750638	3,02156552	3,40307507	10,5785872	0,78661551
CARD10	caspase recruitment domain family, member 10	9,72857168	6,45524457	3,40038785	10,5589015	1
GPR160	G protein-coupled receptor 160	5,13140778	3,53896249	3,39586631	10,5258607	1
TSC2	tuberous sclerosis 2	11,7009023	9,99302164	3,37490897	10,3740619	1
COBL	cordon-bleu homolog (mouse)	5,62931396	3,69984683	3,36922325	10,3332577	1
AKNA	AT-hook transcription factor	4,66286041	3,43871556	3,36320518	10,2902433	0,99998866
VWA5A	von Willebrand factor A domain containing 5A	10,1760161	7,38064502	3,36052745	10,2711616	1
ROBO2	roundabout, axon guidance receptor, homolog 2 (Drosophila)	4,12676449	3,28529208	3,35502064	10,232031	0,99045176
BMP5	bone morphogenetic protein 5	8,7458909	5,12939668	3,35313513	10,2186672	1
FBXO2	F-box protein 2	9,00245022	5,33328883	3,35127628	10,2055093	1
EMR2	egf-like module containing, mucin-like, hormone receptor-like 2	9,41612374	5,80429994	3,34704248	10,1756037	1
OLR1	oxidized low density lipoprotein (lectin-like) receptor 1	9,46290362	5,89202723	3,34571256	10,1662278	1

ABLIM1	actin binding LIM protein 1	12,3017032	11,0189187	3,3382513	10,1137863	0,99999713
CFB	complement factor B	9,53228179	6,06911048	3,33178712	10,0685716	1
C1orf198	chromosome 1 open reading frame 198	11,3889262	9,33541318	3,330887	10,0622916	1
CPS1	carbamoyl-phosphate synthase 1, mitochondrial	12,8462676	11,7393658	3,31853727	9,97652418	0,99987533
C4BPA	complement component 4 binding protein, alpha	3,82019877	3,16037344	3,30729042	9,89905233	0,9100503
RNF125	ring finger protein 125	7,3486384	4,22566497	3,30361985	9,87389873	1
CD36	CD36 molecule (thrombospondin receptor)	5,53759717	3,69060231	3,30295581	9,86935508	1
DCP2	DCP2 decapping enzyme homolog (S. cerevisiae)	9,84945544	6,9165498	3,29530404	9,81714849	1
COL4A6	collagen, type IV, alpha 6	9,48908732	6,03810825	3,2952179	9,81656238	1
DUSP9	dual specificity phosphatase 9	9,56486612	6,22635245	3,29410186	9,80897144	1
HIST3H2BB	histone cluster 3, H2bb	11,4745903	9,57680581	3,29312916	9,80236018	1
CPS1	carbamoyl-phosphate synthase 1, mitochondrial	12,7085677	11,5833837	3,28164966	9,72467248	0,99991156
RHPN1	rhophilin, Rho GTPase binding protein 1	9,13046682	5,52470879	3,27887007	9,7059543	1
EPPK1	epiplakin 1	9,80770584	6,85959458	3,27589368	9,68595081	1
RARRES3	retinoic acid receptor responder (tazarotene induced) 3	8,54868141	5,04723377	3,27524147	9,68157301	1
CKB	creatine kinase, brain	12,6017511	11,4510723	3,27420935	9,67464921	0,99994614
EFHD1	EF-hand domain family, member D1	7,95173811	4,55304945	3,26604271	9,6200388	1
INSR	insulin receptor	10,1752296	7,47817613	3,26452934	9,60995279	1
C11orf9	chromosome 11 open reading frame 9	8,87946997	5,32121168	3,26048429	9,58304596	1
HIST3H2BB	histone cluster 3, H2bb	11,4585176	9,58471098	3,24863096	9,50463323	1
VAV1	vav 1 guanine nucleotide exchange factor	8,6146402	5,12455893	3,24851882	9,50389452	1
GPR56	G protein-coupled receptor 56	11,8760305	10,4112167	3,24369371	9,47216171	0,99999997
MLXIP	MLX interacting protein	11,27885	9,18093572	3,23774252	9,43316904	1
PARP12	poly (ADP-ribose) polymerase family, member 12	10,5346848	7,9525576	3,23618845	9,42301313	1
ARPP21	cAMP-regulated phosphoprotein, 21kDa	5,79078993	3,81061338	3,2357219	9,41996628	1
HLA-B	major histocompatibility complex, class I, B	11,7566139	10,2064477	3,2341734	9,40986094	1
SPINT2	serine peptidase inhibitor, Kunitz type, 2	7,44614604	4,29831725	3,23282736	9,40108561	1
EYA1	eyes absent homolog 1 (Drosophila)	10,9039631	8,45215297	3,2313848	9,39169009	1
KLRC3	killer cell lectin-like receptor subfamily C, member 3	3,84655872	3,18513067	3,22868126	9,37410698	0,91144186
GPR158	G protein-coupled receptor 158	5,84144788	3,83514236	3,22269285	9,33527713	1
STRA6	stimulated by retinoic acid gene 6 homolog (mouse)	8,84468655	5,33056893	3,22167447	9,32868982	1

ADAM15	ADAM metallopeptidase domain 15	11,9108813	10,4838208	3,22127074	9,32607959	0,99999991
CDH19	cadherin 19, type 2	4,86525288	3,51848787	3,22040829	9,32050606	0,99999937
TBX1	T-box 1	8,63992835	5,17192451	3,21902984	9,31160488	1
STAG3L3	stromal antigen 3-like 3	11,3571769	9,39257613	3,21037728	9,25592568	1
DNAJC22	DnaJ (Hsp40) homolog, subfamily C, member 22	7,98661959	4,63536768	3,20602467	9,22804263	1
RPTOR	regulatory associated protein of MTOR, complex 1	3,99124889	3,25632876	3,2007925	9,19463625	0,96026884
GNG4	guanine nucleotide binding protein (G protein), gamma 4	8,84002971	5,35273442	3,19481503	9,1566192	1
RBMXL1	RNA binding motif protein, X-linked-like 1	4,02678175	3,27249811	3,19457885	9,15512035	0,96895102
CASP10	caspase 10, apoptosis-related cysteine peptidase	4,61589065	3,46316453	3,1932844	9,14690963	0,99994827
SNX10	sorting nexin 10	4,27636271	3,36751259	3,18381969	9,08709833	0,9962897
HOXB3	homeobox B3	3,94579812	3,2409149	3,17127958	9,00845428	0,94342014
ROBO2	roundabout, axon guidance receptor, homolog 2 (Drosophila)	3,93318368	3,23592239	3,16645899	8,97840386	0,93858681
CYP4X1	cytochrome P450, family 4, subfamily X, polypeptide 1	6,93985315	4,18630516	3,15966082	8,93619593	1
RNF43	ring finger protein 43	6,61646855	4,10765875	3,15618776	8,91470935	1
C4BPB	complement component 4 binding protein, beta	8,69371006	5,27591658	3,15306112	8,89541012	1
AGXT2L1	alanine-glyoxylate aminotransferase 2-like 1	7,81318039	4,55497474	3,14603204	8,85217543	1
TBX1	T-box 1	8,23042544	4,90470189	3,14592035	8,85149014	1
PTPRR	protein tyrosine phosphatase, receptor type, R	7,56779987	4,41236088	3,1374707	8,7997998	1
HLA-B	major histocompatibility complex, class I, B	11,9372654	10,5776952	3,13584529	8,78989111	0,99999954
VASH2	vasohibin 2	4,85722388	3,53717913	3,13560637	8,78843553	0,99999881
ALDOC	aldolase C, fructose-bisphosphate	9,55654272	6,52178385	3,13085095	8,75951475	1
ARID5B	AT rich interactive domain 5B (MRF1-like)	9,93324164	7,27443705	3,12792355	8,74175866	1
USP18	ubiquitin specific peptidase 18	7,74784271	4,52681194	3,12543371	8,72668493	1
NRARP	NOTCH-regulated ankyrin repeat protein	7,46470863	4,37379582	3,12259854	8,70955215	1
FKBP5	FK506 binding protein 5	10,3750109	7,86668131	3,11948352	8,69076707	1
KRT18	keratin 18	11,7750933	10,3187575	3,11853345	8,68504577	0,99999996
GLIS3	GLIS family zinc finger 3	6,85573232	4,18438564	3,11825283	8,68335658	1
RRAGD	Ras-related GTP binding D	3,84947432	3,20463854	3,11040882	8,63627281	0,89610044
HOOK1	hook homolog 1 (Drosophila)	4,42049059	3,42665727	3,11012592	8,63457949	0,9990632
FIBP	fibroblast growth factor (acidic) intracellular binding protein	5,64090405	3,80479355	3,10014781	8,57506622	1
C4BPB	complement component 4 binding protein, beta	8,63029973	5,27591658	3,1000234	8,57432678	1

ARPP21	cAMP-regulated phosphoprotein, 21kDa	4,08405656	3,31307617	3,09373575	8,5370389	0,97515228
C4orf19	chromosome 4 open reading frame 19	6,7110629	4,16227825	3,08657172	8,49475134	1
FABP3	fatty acid binding protein 3, muscle and heart (mammary-derived growth in	9,087829	5,70324361	3,08548648	8,48836376	1
SEMA6D	sema domain, transmembrane domain (TM), and cytoplasmic domain, (sem	6,55946426	4,1236137	3,08545937	8,48820426	1
OLR1	oxidized low density lipoprotein (lectin-like) receptor 1	8,96063525	5,56949525	3,08523745	8,48689868	1
RASD2	RASD family, member 2	6,76135229	4,17754959	3,08106993	8,46241788	1
PSME1	proteasome (prosome, macropain) activator subunit 1 (PA28 alpha)	11,9947199	10,6985033	3,08078486	8,4607459	0,99999791
INHBE	inhibin, beta E	7,1916354	4,2977551	3,0782174	8,4457023	1
FAM46C	family with sequence similarity 46, member C	4,82480019	3,54317097	3,0772179	8,43985317	0,99999705
ZNF445	zinc finger protein 445	4,54862061	3,47016727	3,07659751	8,43622463	0,99978987
TRIM10	tripartite motif containing 10	6,17765293	4,01741462	3,07273667	8,41367837	1
TMEM200C	transmembrane protein 200C	7,31518705	4,34448302	3,07160795	8,40709833	1
FLRT3	fibronectin leucine rich transmembrane protein 3	5,3982569	3,72191385	3,06969659	8,39596753	1
SORT1	sortilin 1	9,53251058	6,59822644	3,0556345	8,31452878	1
KLRK1	killer cell lectin-like receptor subfamily K, member 1	6,1586601	4,01933428	3,05332431	8,30122537	1
C9orf84	chromosome 9 open reading frame 84	4,6236987	3,49698169	3,04757312	8,26819903	0,99991409
JAKMIP2	janus kinase and microtubule interacting protein 2	4,36519182	3,42207498	3,04753979	8,26800803	0,99780956
ARID5B	AT rich interactive domain 5B (MRF1-like)	11,4459113	9,7485482	3,04656041	8,26239714	1
NAAA	N-acylethanolamine acid amidase	3,74616536	3,16051628	3,04613124	8,25993964	0,82118002
APOL6	apolipoprotein L, 6	9,30530568	6,09689127	3,04298546	8,24194859	1
RNF43	ring finger protein 43	8,03569501	4,8352405	3,04146515	8,23326781	1
OPN3	opsin 3	9,33651041	6,18618066	3,02879624	8,16128454	1
FGFBP3	fibroblast growth factor binding protein 3	4,30850516	3,40740225	3,02833286	8,15866363	0,99584201
IFI44	interferon-induced protein 44	4,90640295	3,5780764	3,02625164	8,14690247	0,99999903
EYA1	eyes absent homolog 1 (Drosophila)	10,1556844	7,68759086	3,02409059	8,13470816	1
CFB	complement factor B	8,69302942	5,40983603	3,01209592	8,06735598	1
MLXIP	MLX interacting protein	11,1714018	9,19839085	3,0081814	8,04549619	1
VWA5A	von Willebrand factor A domain containing 5A	7,14868676	4,32437795	3,00387108	8,02149463	1

Downregulated

symbol	gene name	5 µM RZ2	control	log2FC	FC	probDE

PKIB	protein kinase (cAMP-dependent, catalytic) inhibitor beta	4,37717979	7,1219954	-3,0035553	-8,0197393	1
PHLDA3	pleckstrin homology-like domain, family A, member 3	7,14596161	9,77544326	-3,0064071	-8,0356072	1
CLIC2	chloride intracellular channel 2	4,79863808	7,81093231	-3,0115228	-8,0641518	1
ZNF700	zinc finger protein 700	3,45879763	4,56939939	-3,015027	-8,0837631	0,99994196
KIAA0825	KIAA0825	3,46647842	4,59859438	-3,0163987	-8,0914527	0,9999623
SEMA5A	sema domain, seven thrombospondin repeats (type 1 and type 1-like), trans	4,2800536	6,86367096	-3,016704	-8,0931651	1
BACE2	beta-site APP-cleaving enzyme 2	7,23881767	9,84546473	-3,0172708	-8,0963453	1
PAPPA	pregnancy-associated plasma protein A, pappalysin 1	6,32678277	9,35347817	-3,0181313	-8,1011756	1
ANXA1	annexin A1	12,0995311	13,079358	-3,018861	-8,1052743	0,99926963
SDC2	syndecan 2	6,9878407	9,68860455	-3,0201179	-8,1123387	1
SEMA3D	sema domain, immunoglobulin domain (Ig), short basic domain, secreted, (s	4,04087507	6,1522485	-3,0217789	-8,1216839	1
DMBT1	deleted in malignant brain tumors 1	4,66011977	7,65570495	-3,0228397	-8,1276578	1
GPR63	G protein-coupled receptor 63	3,48665886	4,68043832	-3,0244517	-8,1367443	0,99998909
KRT7	keratin 7	12,5623704	13,484848	-3,0247381	-8,1383599	0,998029
ACVR1	activin A receptor, type I	7,44200362	9,99972474	-3,0247775	-8,1385821	1
FOXQ1	forkhead box Q1	5,2933139	8,42337106	-3,0269574	-8,150889	1
AK5	adenylate kinase 5	4,50357017	7,42792596	-3,0288807	-8,1617626	1
C11orf74	chromosome 11 open reading frame 74	7,59309096	10,1268108	-3,0319505	-8,1791478	1
KLF6	Kruppel-like factor 6	7,39180349	9,96701773	-3,0322133	-8,1806379	1
TBC1D19	TBC1 domain family, member 19	6,47748856	9,43757524	-3,0332347	-8,1864313	1
MALL	mal, T-cell differentiation protein-like	5,18207386	8,28784019	-3,0347655	-8,1951223	1
YWHAQ	tyrosine 3-monooxygenase/tryptophan 5-monooxygenase activation protei	10,070219	11,6566891	-3,0354391	-8,1989496	1
CHSY3	chondroitin sulfate synthase 3	3,71232891	5,41154271	-3,0354793	-8,1991778	1
ARHGDIB	Rho GDP dissociation inhibitor (GDI) beta	4,20292269	6,64587359	-3,0359173	-8,2016675	1
CAV1	caveolin 1, caveolae protein, 22kDa	10,1098459	11,6802227	-3,0422728	-8,2378781	1
CAV1	caveolin 1, caveolae protein, 22kDa	10,1098459	11,6802227	-3,0422728	-8,2378781	1
RASSF8	Ras association (RalGDS/AF-6) domain family (N-terminal) member 8	8,15316087	10,621097	-3,0432862	-8,2436666	1
COL1A1	collagen, type I, alpha 1	10,6022476	11,9638391	-3,0437552	-8,2463469	0,99999977
HHAT	hedgehog acyltransferase	5,20353741	8,32816633	-3,0456187	-8,257006	1
SERPINE2	serpin peptidase inhibitor, clade E (nexin, plasminogen activator inhibitor ty	6,64107775	9,52681982	-3,0482859	-8,2722849	1
PLXDC2	plexin domain containing 2	6,74245413	9,57783452	-3,0496347	-8,2800225	1

COL4A1	collagen, type IV, alpha 1	9,25932051	11,2819859	-3,050373	-8,2842608	1
PHLDA1	pleckstrin homology-like domain, family A, member 1	6,5817102	9,50107307	-3,0518077	-8,2925037	1
S1PR1	sphingosine-1-phosphate receptor 1	4,16235946	6,5410442	-3,0527295	-8,2978034	1
ARMCX2	armadillo repeat containing, X-linked 2	5,0575301	8,16135622	-3,0587439	-8,3324681	1
CLDN1	claudin 1	5,33535942	8,52181342	-3,0617788	-8,3500148	1
MSRB3	methionine sulfoxide reductase B3	5,67240663	8,9451383	-3,0666353	-8,3781708	1
SDCBP	syndecan binding protein (syntenin)	3,61425687	5,17324956	-3,0685697	-8,389412	1
DKK1	dickkopf homolog 1 (Xenopus laevis)	6,41660837	9,43690789	-3,0688498	-8,391041	1
C11orf74	chromosome 11 open reading frame 74	6,84884307	9,64834134	-3,0702237	-8,3990355	1
PROC	protein C (inactivator of coagulation factors Va and VIIIa)	7,90714084	10,4318965	-3,0733123	-8,4170362	1
ZBTB38	zinc finger and BTB domain containing 38	7,96782207	10,4862326	-3,0745189	-8,4240784	1
KLF6	Kruppel-like factor 6	8,23536231	10,7104133	-3,0745322	-8,4241564	1
POMP	proteasome maturation protein	3,7431229	5,52167986	-3,0758702	-8,4319724	1
PHF14	PHD finger protein 14	3,36480433	4,29207669	-3,0765917	-8,4361909	0,99817144
FHIT	fragile histidine triad gene	3,97349209	6,0473825	-3,0783836	-8,4466754	1
APBB1	amyloid beta (A4) precursor protein-binding, family B, member 1 (Fe65)	4,97991592	8,09208194	-3,0787436	-8,4487831	1
ARHGAP29	Rho GTPase activating protein 29	4,26063907	6,90476945	-3,0798772	-8,4554243	1
ZNF100	zinc finger protein 100	3,45910225	4,62364261	-3,0816576	-8,4658655	0,99998037
POLI	polymerase (DNA directed) iota	3,28881485	4,08235444	-3,0853952	-8,4878266	0,98681934
CD226	CD226 molecule	3,37571515	4,33193982	-3,0860655	-8,4917714	0,998878
TMEM154	transmembrane protein 154	3,40720607	4,43782798	-3,087201	-8,4984575	0,99972411
HAS2	hyaluronan synthase 2	4,11160811	6,43869067	-3,0917018	-8,5250119	1
RPS6KA2	ribosomal protein S6 kinase, 90kDa, polypeptide 2	6,00212966	9,24162676	-3,0932421	-8,5341182	1
MATN3	matrilin 3	3,67537907	5,37008199	-3,0938849	-8,5379215	1
STXBP6	syntaxin binding protein 6 (amisyn)	4,13311653	6,50995992	-3,0947621	-8,5431144	1
IL24	interleukin 24	3,62867343	5,24529705	-3,099314	-8,5701114	1
PHLDA1	pleckstrin homology-like domain, family A, member 1	4,6618421	7,74737685	-3,100963	-8,5799131	1
FHL2	four and a half LIM domains 2	8,10796042	10,6286306	-3,1037612	-8,5965704	1
FSTL4	follistatin-like 4	5,85805823	9,15272979	-3,104613	-8,6016477	1
SERPINB5	serpin peptidase inhibitor, clade B (ovalbumin), member 5	8,32074789	10,7998504	-3,1089708	-8,627669	1
ANTXR1	anthrax toxin receptor 1	8,13359119	10,6553358	-3,1111162	-8,6405082	1

ZNF699	zinc finger protein 699	3,23005166	3,95067991	-3,1131691	-8,652812	0,96562863
EMB	embigin	5,78501424	9,1071994	-3,1179476	-8,6815196	1
SDC2	syndecan 2	6,35791789	9,44943072	-3,1192494	-8,6893569	1
MYOCD	myocardin	3,79945418	5,69136978	-3,1208539	-8,6990261	1
NPR1	natriuretic peptide receptor A/guanylate cyclase A (atrionatriuretic peptide	7,55504208	10,1643488	-3,1212295	-8,7012915	1
C18orf1	chromosome 18 open reading frame 1	3,63687891	5,29060022	-3,1223854	-8,7082653	1
NID1	nidogen 1	5,03747665	8,21376856	-3,1258745	-8,7293517	1
BMP6	bone morphogenetic protein 6	8,30443533	10,8008432	-3,127572	-8,7396289	1
RND3	Rho family GTPase 3	8,50417721	10,9388523	-3,1289465	-8,7479592	1
GREM1	gremlin 1	5,35190002	8,63183464	-3,1333872	-8,7749276	1
CTAG2	cancer/testis antigen 2	5,32239232	8,59264943	-3,1338507	-8,7777468	1
NGF	nerve growth factor (beta polypeptide)	5,58223495	8,92481277	-3,1350351	-8,7849562	1
BANK1	B-cell scaffold protein with ankyrin repeats 1	3,36624729	4,33149494	-3,1363596	-8,793025	0,99904235
RHOB	ras homolog gene family, member B	8,58236024	10,9928184	-3,1382245	-8,8043986	1
NEDD4	neural precursor cell expressed, developmentally down-regulated 4	3,93595856	6,03139116	-3,1488728	-8,8696234	1
VCAN	versican	3,61836911	5,2615657	-3,1492996	-8,8722475	1
MYOCD	myocardin	4,60352771	7,7307002	-3,1519332	-8,8884581	1
FSTL1	follistatin-like 1	8,71356558	11,0769505	-3,1547221	-8,9056573	1
WNT5A	wingless-type MMTV integration site family, member 5A	5,33057697	8,63248594	-3,1572748	-8,921429	1
QKI	quaking homolog, KH domain RNA binding (mouse)	4,11290555	6,5410999	-3,1603094	-8,9402144	1
AOX1	aldehyde oxidase 1	4,78843637	7,96930517	-3,1656085	-8,9731127	1
MCTP1	multiple C2 domains, transmembrane 1	6,05634414	9,34304661	-3,1709115	-9,0061565	1
ARHGEF5	Rho guanine nucleotide exchange factor (GEF) 5	4,14374597	6,66495546	-3,1740931	-9,0260396	1
SPATS2L	spermatogenesis associated, serine-rich 2-like	3,55340036	5,07271885	-3,1743593	-9,0277051	1
LGALS8	lectin, galactoside-binding, soluble, 8	6,97543725	9,79707268	-3,1760095	-9,0380373	1
IRS1	insulin receptor substrate 1	3,47328563	4,76205159	-3,1761078	-9,0386531	0,99999864
PID1	phosphotyrosine interaction domain containing 1	5,06972699	8,31168982	-3,1763369	-9,0400883	1
GNG7	guanine nucleotide binding protein (G protein), gamma 7	4,05532787	6,38606392	-3,1782441	-9,0520469	1
SLC30A4	solute carrier family 30 (zinc transporter), member 4	3,89890542	5,98191329	-3,1867898	-9,1058255	1
SLC12A8	solute carrier family 12 (potassium/chloride transporters), member 8	7,90135722	10,5207299	-3,1947403	-9,1561451	1
AMIGO2	adhesion molecule with Ig-like domain 2	5,19664417	8,50059924	-3,1970543	-9,1708429	1

CYP39A1	cytochrome P450, family 39, subfamily A, polypeptide 1	4,25147811	7,07283939	-3,1991232	-9,1840036	1
SERPINB5	serpin peptidase inhibitor, clade B (ovalbumin), member 5	8,83076769	11,1624774	-3,19947	-9,1862113	1
ADAM19	ADAM metallopeptidase domain 19	5,64401553	9,06650068	-3,2018093	-9,2011187	1
EMP1	epithelial membrane protein 1	5,42734166	8,81753507	-3,2033297	-9,2108206	1
APBB1	amyloid beta (A4) precursor protein-binding, family B, member 1 (Fe65)	5,02488378	8,28853293	-3,2043457	-9,2173097	1
NEGR1	neuronal growth regulator 1	4,05148648	6,41036679	-3,2049672	-9,2212812	1
SEMA5A	sema domain, seven thrombospondin repeats (type 1 and type 1-like), trans	3,26313474	4,06771083	-3,2069443	-9,2339265	0,98872208
KLF2	Kruppel-like factor 2 (lung)	4,59009496	7,77733213	-3,2073521	-9,2365371	1
PROC	protein C (inactivator of coagulation factors Va and VIIIa)	7,83481442	10,4752962	-3,2113909	-9,2624311	1
SDC2	syndecan 2	7,4548647	10,1556286	-3,215173	-9,2867451	1
MAGED1	melanoma antigen family D, 1	3,87714016	5,95917584	-3,2152207	-9,2870522	1
ETV5	ets variant 5	5,1971612	8,52554639	-3,2170556	-9,2988709	1
RGNEF	190 kDa guanine nucleotide exchange factor	3,96947063	6,19381725	-3,2188858	-9,3106752	1
KRCC1	lysine-rich coiled-coil 1	5,02041757	8,29985047	-3,2189162	-9,3108711	1
TNFAIP8L3	tumor necrosis factor, alpha-induced protein 8-like 3	5,51210803	8,94240057	-3,2196142	-9,3153773	1
FHL2	four and a half LIM domains 2	8,54782424	11,0237819	-3,2206257	-9,3219108	1
RHOB	ras homolog gene family, member B	9,83656015	11,616204	-3,2209966	-9,3243076	1
EGR2	early growth response 2	3,42665727	4,61255844	-3,2223093	-9,3327959	0,99998721
C1orf21	chromosome 1 open reading frame 21	4,27433132	7,18264772	-3,2230137	-9,3373532	1
MPP4	membrane protein, palmitoylated 4 (MAGUK p55 subfamily member 4)	3,61407436	5,3203664	-3,2249207	-9,3497038	1
ODZ2	odz, odd Oz/ten-m homolog 2 (Drosophila)	4,7242211	7,97232895	-3,2349337	-9,4148214	1
ALPK2	alpha-kinase 2	4,21729738	7,02637391	-3,2381511	-9,4358412	1
SDC2	syndecan 2	6,32272002	9,52969736	-3,2412292	-9,4559944	1
GLIPR1	GLI pathogenesis-related 1	5,55172962	9,01606995	-3,2436399	-9,4718085	1
UBASH3B	ubiquitin associated and SH3 domain containing B	4,4056654	7,53515154	-3,2464789	-9,490466	1
IL8	interleukin 8	3,46510146	4,79407232	-3,2465739	-9,4910905	0,9999995
AKR1C2	aldo-keto reductase family 1, member C2 (dihydrodiol dehydrogenase 2; bil	3,39570817	4,5119972	-3,2496674	-9,5114637	0,99994822
GLS	glutaminase	7,42585626	10,1602887	-3,2511061	-9,5209537	1
CYP2B7P1	cytochrome P450, family 2, subfamily B, polypeptide 7 pseudogene 1	4,29208753	7,28435385	-3,2547524	-9,5450479	1
NPY1R	neuropeptide Y receptor Y1	3,54053126	5,1072204	-3,2562362	-9,5548695	1
HDGFRP3	hepatoma-derived growth factor, related protein 3	8,30250503	10,8898336	-3,2579015	-9,5659051	1

PAPPA	pregnancy-associated plasma protein A, pappalysin 1	5,93644962	9,35267651	-3,2589516	-9,5728707	1
IL15	interleukin 15	3,50633782	4,97991592	-3,2610037	-9,5864967	0,99999999
TNFRSF12A	tumor necrosis factor receptor superfamily, member 12A	10,6710948	12,0844793	-3,2622656	-9,5948856	0,99999994
C1R	complement component 1, r subcomponent	7,60877662	10,3207348	-3,2650696	-9,6135524	1
NNMT	nicotinamide N-methyltransferase	10,7282493	12,12155	-3,265441	-9,6160272	0,9999999
RND3	Rho family GTPase 3	7,2926382	10,0728129	-3,2684768	-9,6362832	1
CD59	CD59 molecule, complement regulatory protein	11,6884801	12,8158861	-3,2699366	-9,6460385	0,99995858
COMMD3	COMM domain containing 3	8,90070949	11,2340945	-3,2704035	-9,6491609	1
RAB3B	RAB3B, member RAS oncogene family	5,98547329	9,39158719	-3,2707101	-9,6512117	1
CELF2	CUGBP, Elav-like family member 2	3,20356702	3,94693458	-3,2741408	-9,6741894	0,97404777
ETV5	ets variant 5	3,99139642	6,32826355	-3,279251	-9,7085171	1
AKR1C2	aldo-keto reductase family 1, member C2 (dihydrodiol dehydrogenase 2; bil	3,20740782	3,96062174	-3,2869027	-9,7601457	0,97717872
ANXA10	annexin A10	4,87694921	8,2133094	-3,2939081	-9,8076541	1
CLIC2	chloride intracellular channel 2	3,7090674	5,64090405	-3,2961814	-9,8231205	1
MAGED1	melanoma antigen family D, 1	4,49501277	7,75132942	-3,297397	-9,8314009	1
FAIM3	Fas apoptotic inhibitory molecule 3	4,89408774	8,23815255	-3,2981209	-9,8363354	1
NTN4	netrin 4	4,96291051	8,32150376	-3,2989181	-9,8417723	1
GPR63	G protein-coupled receptor 63	3,53285555	5,12477299	-3,3018237	-9,8616137	1
PITPNC1	phosphatidylinositol transfer protein, cytoplasmic 1	9,1278928	11,3485178	-3,3019537	-9,8625022	1
TMEM159	transmembrane protein 159	3,12800238	3,79000563	-3,3021296	-9,8637045	0,9330491
THBS1	thrombospondin 1	4,03670653	6,49980716	-3,302811	-9,8683645	1
PRR16	proline rich 16	3,78855034	5,84129237	-3,3073665	-9,8995744	1
SGK1	serum/glucocorticoid regulated kinase 1	9,74844036	11,6121948	-3,308987	-9,9107004	1
ADAM19	ADAM metallopeptidase domain 19	4,38480626	7,58598916	-3,3138354	-9,9440626	1
STXBP6	syntaxin binding protein 6 (amisyn)	3,15057936	3,84143382	-3,3148202	-9,9508532	0,95137766
KIF5C	kinesin family member 5C	3,11266739	3,76410024	-3,3210892	-9,994187	0,92491439
BACE2	beta-site APP-cleaving enzyme 2	8,93764778	11,278006	-3,3223459	-10,002897	1
FBLN5	fibulin 5	5,85129543	9,37310278	-3,338292	-10,114071	1
OLFM1	olfactomedin 1	9,80207956	11,6484228	-3,3385407	-10,115816	1
PDE4B	phosphodiesterase 4B, cAMP-specific	4,56126343	7,89261373	-3,3393648	-10,121595	1
PNMA2	paraneoplastic antigen MA2	3,57799957	5,32186989	-3,3418436	-10,139001	1

IGFBP3	insulin-like growth factor binding protein 3	9,29644664	11,4395994	-3,3487248	-10,187476	1
SGK1	serum/glucocorticoid regulated kinase 1	9,61423761	11,5706012	-3,3491872	-10,190742	1
EDIL3	EGF-like repeats and discoidin I-like domains 3	8,4361351	11,0397597	-3,3518217	-10,209368	1
AMIGO2	adhesion molecule with Ig-like domain 2	4,86854916	8,27005212	-3,3530304	-10,217925	1
SNTB1	syntrophin, beta 1 (dystrophin-associated protein A1, 59kDa, basic compone	3,22584621	4,03153478	-3,3531894	-10,219051	0,98889823
FAM82A1	family with sequence similarity 82, member A1	4,23336292	7,28661229	-3,3613503	-10,277022	1
KIT	v-kit Hardy-Zuckerman 4 feline sarcoma viral oncogene homolog	3,31280305	4,28820148	-3,3615847	-10,278692	0,99920664
PLK2	polo-like kinase 2	9,06970802	11,3540492	-3,3620231	-10,281815	1
OLFM1	olfactomedin 1	9,14939066	11,3883212	-3,3657204	-10,308199	1
SLC9A2	solute carrier family 9 (sodium/hydrogen exchanger), member 2	4,49854865	7,83932476	-3,3661371	-10,311177	1
SERPINE2	serpin peptidase inhibitor, clade E (nexin, plasminogen activator inhibitor ty	4,14138818	6,98483304	-3,3699009	-10,338113	1
LETM2	leucine zipper-EF-hand containing transmembrane protein 2	6,22356814	9,58912914	-3,3712494	-10,34778	1
SERPINE2	serpin peptidase inhibitor, clade E (nexin, plasminogen activator inhibitor ty	8,22835739	10,9176605	-3,3744579	-10,370819	1
MCTP1	multiple C2 domains, transmembrane 1	6,14455055	9,5604884	-3,3781295	-10,397246	1
MFAP5	microfibrillar associated protein 5	3,93161701	6,28476576	-3,3830856	-10,433025	1
SEMA3D	sema domain, immunoglobulin domain (Ig), short basic domain, secreted, (s	3,8930522	6,18048846	-3,3850526	-10,447259	1
FHL2	four and a half LIM domains 2	9,35997	11,4820805	-3,385867	-10,453159	1
TM4SF1	transmembrane 4 L six family member 1	9,77657405	11,6573598	-3,3884914	-10,472191	1
PAPPAS	PAPPA antisense RNA (non-protein coding)	4,61988166	8,02148846	-3,388663	-10,473437	1
ANTXR1	anthrax toxin receptor 1	7,24783666	10,1322768	-3,3901296	-10,484089	1
LEPR	leptin receptor	3,9758263	6,4356755	-3,3972445	-10,535921	1
PAPPA	pregnancy-associated plasma protein A, pappalysin 1	5,61539239	9,25504933	-3,4005521	-10,560103	1
TIAM1	T-cell lymphoma invasion and metastasis 1	3,51494407	5,1591919	-3,4015074	-10,567098	1
RAET1E	retinoic acid early transcript 1E	4,0081675	6,5528486	-3,4050634	-10,593177	1
C18orf1	chromosome 18 open reading frame 1	3,69717029	5,71491894	-3,406386	-10,602893	1
MAP3K13	mitogen-activated protein kinase kinase kinase 13	3,18359563	3,94768465	-3,4085708	-10,618962	0,98023588
SYT17	synaptotagmin XVII	3,32663533	4,3680371	-3,41463	-10,663654	0,99977552
FSTL4	follistatin-like 4	6,11898301	9,57885976	-3,4147019	-10,664186	1
IL13RA2	interleukin 13 receptor, alpha 2	3,24299347	4,10682726	-3,4226545	-10,723132	0,99522482
AFF3	AF4/FMR2 family, member 3	3,24693427	4,1183648	-3,4236495	-10,730531	0,99573061
CLDN1	claudin 1	3,48977624	5,08072083	-3,4238148	-10,73176	1

SDC2	syndecan 2	7,5655352	10,4082767	-3,4251282	-10,741535	1
APBB1	amyloid beta (A4) precursor protein-binding, family B, member 1 (Fe65)	4,53334026	7,95787552	-3,4270297	-10,755702	1
CAV1	caveolin 1, caveolae protein, 22kDa	10,2449901	11,9004253	-3,4317412	-10,790884	1
PKIB	protein kinase (cAMP-dependent, catalytic) inhibitor beta	3,08734324	3,74400738	-3,4327198	-10,798207	0,92904238
WNT5A	wingless-type MMTV integration site family, member 5A	7,75520191	10,579031	-3,4349739	-10,815091	1
ZNF599	zinc finger protein 599	3,17650495	3,94123228	-3,4381059	-10,838596	0,98040319
AMOT	angiomotin	3,4936142	5,11355491	-3,4395207	-10,849229	1
RAB3B	RAB3B, member RAS oncogene family	4,11786412	7,02512148	-3,4425937	-10,872364	1
JUN	jun proto-oncogene	4,93827651	8,46186564	-3,4464275	-10,901294	1
GALNT1	UDP-N-acetyl-alpha-D-galactosamine:polypeptide N-acetylgalactosaminyltra	9,56596199	11,5922715	-3,4476998	-10,910912	1
DPY19L2	dpy-19-like 2 (C. elegans)	3,36183224	4,52962509	-3,4493291	-10,923241	0,9999816
SGK1	serum/glucocorticoid regulated kinase 1	9,94345256	11,7590151	-3,4531755	-10,952403	1
PTHLH	parathyroid hormone-like hormone	3,1959887	3,9962476	-3,4574002	-10,984522	0,98801155
FRS2	fibroblast growth factor receptor substrate 2	3,18693487	3,97439233	-3,4595072	-11,000577	0,9856476
TMEM74	transmembrane protein 74	3,58858972	5,472581	-3,4612397	-11,013794	1
IL7R	interleukin 7 receptor	7,46579525	10,3541932	-3,4621471	-11,020724	1
ZNF558	zinc finger protein 558	2,9254085	3,45489742	-3,4643429	-11,037511	0,74702904
IRS1	insulin receptor substrate 1	4,19963594	7,35946297	-3,4672604	-11,059854	1
PDGFC	platelet derived growth factor C	5,13219227	8,74461026	-3,4677955	-11,063957	1
SERPINE2	serpin peptidase inhibitor, clade E (nexin, plasminogen activator inhibitor ty	9,38399945	11,5329095	-3,4783897	-11,145502	1
KHDRBS3	KH domain containing, RNA binding, signal transduction associated 3	5,92231761	9,54070317	-3,4792884	-11,152447	1
MGLL	monoglyceride lipase	7,03340113	10,0570327	-3,4811741	-11,167033	1
DOCK2	dedicator of cytokinesis 2	4,47314217	7,94490729	-3,4846214	-11,193749	1
DNER	delta/notch-like EGF repeat containing	5,99256256	9,58245109	-3,4881106	-11,220854	1
P2RY1	purinergic receptor P2Y, G-protein coupled, 1	3,25816348	4,18719552	-3,49883	-11,304537	0,99822122
AGTR1	angiotensin II receptor, type 1	3,10294662	3,79526371	-3,4991126	-11,306752	0,95217237
BMP6	bone morphogenetic protein 6	7,63773964	10,528216	-3,500421	-11,31701	1
CCL2	chemokine (C-C motif) ligand 2	5,01084063	8,62394572	-3,5040154	-11,345242	1
AFP	alpha-fetoprotein	3,12816414	3,85335266	-3,5093996	-11,387661	0,96747139
PAPPA	pregnancy-associated plasma protein A, pappalysin 1	4,1087365	7,11793546	-3,5130909	-11,416835	1
FAM82A1	family with sequence similarity 82, member A1	3,99982342	6,69556234	-3,5175538	-11,452208	1

MGLL	monoglyceride lipase	7,15490384	10,1636654	-3,5184972	-11,459699	1
NRP1	neuropilin 1	9,0032676	11,4070422	-3,5232972	-11,49789	1
FN1	fibronectin 1	10,7306797	12,2126351	-3,5256162	-11,516386	0,99999999
BHLHE41	basic helix-loop-helix family, member e41	3,45606845	5,04329216	-3,5262551	-11,521488	1
IL15	interleukin 15	3,92662449	6,46629286	-3,5358556	-11,598414	1
CLEC4A	C-type lectin domain family 4, member A	3,22005105	4,09551394	-3,5430165	-11,656126	0,99597662
DDX26B	DEAD/H (Asp-Glu-Ala-Asp/His) box polypeptide 26B	3,35907578	4,59727566	-3,546169	-11,681624	0,99999566
ABCA8	ATP-binding cassette, sub-family A (ABC1), member 8	3,12764008	3,86410366	-3,5468508	-11,687146	0,97165059
SEMA5A	sema domain, seven thrombospondin repeats (type 1 and type 1-like), trans	4,35931267	7,85061564	-3,5490205	-11,704736	1
CKAP4	cytoskeleton-associated protein 4	10,425194	12,0417801	-3,552323	-11,73156	1
BMPER	BMP binding endothelial regulator	3,68014086	5,81568895	-3,5559882	-11,761403	1
GALNT1	UDP-N-acetyl-alpha-D-galactosamine:polypeptide N-acetylgalactosaminyltra	7,08617665	10,1484269	-3,5594617	-11,789754	1
STK32B	serine/threonine kinase 32B	4,99845059	8,68347428	-3,5669092	-11,850773	1
ZNF599	zinc finger protein 599	3,16037344	3,94810886	-3,5670056	-11,851565	0,98570316
MRAP2	melanocortin 2 receptor accessory protein 2	3,58357079	5,56809481	-3,5731021	-11,901752	1
MANSC1	MANSC domain containing 1	3,52580237	5,38205709	-3,5750167	-11,917557	1
FRMD3	FERM domain containing 3	3,19467694	4,04188617	-3,5817541	-11,973343	0,99390102
EDIL3	EGF-like repeats and discoidin I-like domains 3	8,62138291	11,2794452	-3,586302	-12,011147	1
EMP1	epithelial membrane protein 1	5,14942628	8,92102502	-3,5899678	-12,041705	1
CD70	CD70 molecule	3,61282172	5,66943935	-3,5901695	-12,043389	1
ETV5	ets variant 5	3,60653985	5,6533023	-3,5917476	-12,05657	1
AMIGO2	adhesion molecule with Ig-like domain 2	6,50422032	9,87436456	-3,5983316	-12,111718	1
NUDT16P1	nudix (nucleoside diphosphate linked moiety X)-type motif 16 pseudogene 1	4,10892038	7,28370246	-3,6024048	-12,145962	1
WNT5A	wingless-type MMTV integration site family, member 5A	8,38913203	11,1711171	-3,6038499	-12,158134	1
NPNT	nephronectin	3,52520056	5,42198124	-3,6151113	-12,253409	1
ZNF32	zinc finger protein 32	4,04527269	7,0540527	-3,6176995	-12,275412	1
NXPH2	neurexophilin 2	3,68164006	5,8806776	-3,6181141	-12,27894	1
ARL10	ADP-ribosylation factor-like 10	4,05758737	7,11078382	-3,6203328	-12,297838	1
TIMP2	TIMP metallopeptidase inhibitor 2	11,1232807	12,5035644	-3,6224163	-12,315611	0,99999986
FAM126A	family with sequence similarity 126, member A	3,49781757	5,3304753	-3,6244011	-12,332566	1
PSTPIP2	proline-serine-threonine phosphatase interacting protein 2	3,60057491	5,6686825	-3,624543	-12,333779	1

C5orf25	chromosome 5 open reading frame 25	3,46434107	5,19750198	-3,630078	-12,381189	1
TGFB1I1	transforming growth factor beta 1 induced transcript 1	7,39176784	10,4238001	-3,6303285	-12,383339	1
HTRA1	HtrA serine peptidase 1	10,0412258	11,8773141	-3,6421836	-12,485517	1
MGLL	monoglyceride lipase	7,28633649	10,3571259	-3,6501904	-12,555002	1
LCA5	Leber congenital amaurosis 5	3,0995867	3,83404156	-3,6522798	-12,573198	0,97094366
TUB	tubby homolog (mouse)	3,89825694	6,53351165	-3,6524866	-12,575001	1
STXBP6	syntaxin binding protein 6 (amisyn)	4,4555201	8,12387812	-3,6569785	-12,614215	1
FAM176A	family with sequence similarity 176, member A	3,93785548	6,68429318	-3,6576251	-12,619869	1
GALNT1	UDP-N-acetyl-alpha-D-galactosamine:polypeptide N-acetylgalactosaminyltra	8,88644278	11,4306547	-3,6616352	-12,654997	1
C5orf62	chromosome 5 open reading frame 62	4,18310098	7,63229489	-3,6623939	-12,661654	1
IL7R	interleukin 7 receptor	6,9416579	10,1404884	-3,6698945	-12,727653	1
MGLL	monoglyceride lipase	7,4741003	10,5212995	-3,6700351	-12,728894	1
FAIM3	Fas apoptotic inhibitory molecule 3	4,07834678	7,29122432	-3,6702659	-12,73093	1
TLR4	toll-like receptor 4	3,39416669	4,8967692	-3,6709518	-12,736984	0,99999999
CTGF	connective tissue growth factor	7,4933943	10,538156	-3,6711138	-12,738414	1
CD163L1	CD163 molecule-like 1	4,49617541	8,19484116	-3,6739146	-12,763168	1
FSTL1	follistatin-like 1	9,02453768	11,4919953	-3,6820089	-12,834978	1
ZNF44	zinc finger protein 44	3,98705789	6,93470382	-3,6874116	-12,883133	1
SCG5	secretogranin V (7B2 protein)	4,57231542	8,30148981	-3,6877151	-12,885844	1
AASS	aminoadipate-semialdehyde synthase	3,46545841	5,26983154	-3,6894938	-12,901741	1
ABLIM3	actin binding LIM protein family, member 3	5,93531357	9,72463893	-3,689618	-12,902851	1
HSF2BP	heat shock transcription factor 2 binding protein	6,00245022	9,75526735	-3,6936518	-12,938978	1
ARHGAP29	Rho GTPase activating protein 29	3,80295555	6,28702069	-3,6942782	-12,944597	1
IL18R1	interleukin 18 receptor 1	3,08198302	3,81473821	-3,7177916	-13,157301	0,97033306
IGSF10	immunoglobulin superfamily, member 10	3,8466859	6,45275115	-3,7194113	-13,172081	1
TPM1	tropomyosin 1 (alpha)	10,9669644	12,4295568	-3,7213106	-13,189433	0,99999998
NIPAL4	NIPA-like domain containing 4	3,84047442	6,44285054	-3,7271769	-13,243172	1
CPEB1	cytoplasmic polyadenylation element binding protein 1	5,51301326	9,51350393	-3,7299083	-13,268269	1
SMARCA2	SWI/SNF related, matrix associated, actin dependent regulator of chromatir	4,43958299	8,1908312	-3,7322609	-13,289924	1
CNRIP1	cannabinoid receptor interacting protein 1	5,03779901	8,9426429	-3,7325271	-13,292376	1
KIAA1324	KIAA1324	4,65985566	8,45613788	-3,7346627	-13,312067	1

ITGA11	integrin, alpha 11	4,65649518	8,45591563	-3,7379441	-13,34238	1
PDE4B	phosphodiesterase 4B, cAMP-specific	4,93737394	8,81110359	-3,7394436	-13,356255	1
STK32B	serine/threonine kinase 32B	5,61215177	9,60935842	-3,7510776	-13,464396	1
NAV3	neuron navigator 3	4,37494104	8,13222884	-3,7563006	-13,513229	1
RNASE4	ribonuclease, RNase A family, 4	3,00150978	3,65780171	-3,7580476	-13,529603	0,92875537
ANXA3	annexin A3	9,84081874	11,8320513	-3,7590368	-13,538883	1
RHOB	ras homolog gene family, member B	9,89116299	11,8563101	-3,7659415	-13,603835	1
ZNF675	zinc finger protein 675	3,05526424	3,77116254	-3,7685366	-13,628327	0,96361845
NMU	neuromedin U	5,16490509	9,17604583	-3,7755848	-13,69507	1
FOSL1	FOS-like antigen 1	6,67109765	10,0800003	-3,7821093	-13,757146	1
SERPINE2	serpin peptidase inhibitor, clade E (nexin, plasminogen activator inhibitor ty	8,87210223	11,4864577	-3,7844856	-13,779824	1
TIMP3	TIMP metallopeptidase inhibitor 3	8,26863464	11,216374	-3,7849763	-13,784512	1
FBXO25	F-box protein 25	4,37744446	8,18555237	-3,7979649	-13,909174	1
GPR137B	G protein-coupled receptor 137B	3,62013657	5,89822888	-3,7996153	-13,925095	1
COMMD3	COMM domain containing 3	9,50893348	11,7209439	-3,8090451	-14,016411	1
CYP2B7P1	cytochrome P450, family 2, subfamily B, polypeptide 7 pseudogene 1	3,97465909	7,11862578	-3,8135836	-14,060574	1
ZNF229	zinc finger protein 229	3,22315015	4,23990081	-3,8199908	-14,123158	0,99963882
CKAP4	cytoskeleton-associated protein 4	10,891303	12,4133426	-3,8226329	-14,149046	1
FRAS1	Fraser syndrome 1	3,6317833	5,95573813	-3,8245449	-14,16781	1
BDNF	brain-derived neurotrophic factor	7,28909358	10,495492	-3,8294068	-14,215637	1
AMOT	angiomotin	3,28493322	4,4927406	-3,8312547	-14,233857	0,99999178
CTHRC1	collagen triple helix repeat containing 1	4,30702147	8,12459916	-3,8321561	-14,242753	1
SERPINE2	serpin peptidase inhibitor, clade E (nexin, plasminogen activator inhibitor ty	11,2729039	12,6756818	-3,8322567	-14,243746	0,99999992
PTHLH	parathyroid hormone-like hormone	4,16297223	7,85201197	-3,8367897	-14,28857	1
NMU	neuromedin U	8,2802027	11,2564781	-3,8382509	-14,303049	1
KCTD16	potassium channel tetramerisation domain containing 16	3,30295139	4,58743005	-3,8413953	-14,334258	0,99999849
KCTD14	potassium channel tetramerisation domain containing 14	4,0204055	7,40255653	-3,8517919	-14,437929	1
FLI1	Friend leukemia virus integration 1	3,73633033	6,2778528	-3,8527781	-14,447802	1
CPEB1	cytoplasmic polyadenylation element binding protein 1	4,3594193	8,23033385	-3,8563371	-14,483487	1
UBASH3B	ubiquitin associated and SH3 domain containing B	5,85628527	9,83274461	-3,8603086	-14,523412	1
COL4A4	collagen, type IV, alpha 4	3,26228214	4,4196101	-3,8675462	-14,596456	0,99997734

MYH10	myosin, heavy chain 10, non-muscle	3,9399881	7,09807624	-3,8855669	-14,779923	1
HKR1	HKR1, GLI-Kruppel zinc finger family member	4,16281855	7,93257911	-3,8907567	-14,833187	1
TP63	tumor protein p63	3,33489037	4,8200521	-3,8974343	-14,902002	0,99999999
EMP1	epithelial membrane protein 1	3,93345825	7,12141184	-3,9130437	-15,064111	1
CTGF	connective tissue growth factor	7,34294617	10,604282	-3,9164691	-15,099921	1
CGA	glycoprotein hormones, alpha polypeptide	7,02851159	10,3833841	-3,9272511	-15,213193	1
TMEM200A	transmembrane protein 200A	3,30295139	4,68405022	-3,9433809	-15,384236	0,99999986
FOSL1	FOS-like antigen 1	7,14643697	10,4831541	-3,9531134	-15,48837	1
HHAT	hedgehog acyltransferase	4,05893555	7,74833353	-3,9559299	-15,518636	1
PPP2R3C	protein phosphatase 2, regulatory subunit B'', gamma	3,00150978	3,7090674	-3,9584388	-15,545647	0,95981493
ZCCHC18	zinc finger, CCHC domain containing 18	3,39508649	5,26665074	-3,9683597	-15,652918	1
SMARCA2	SWI/SNF related, matrix associated, actin dependent regulator of chromatin	4,11982225	7,95036917	-3,9712807	-15,684642	1
COL4A4	collagen, type IV, alpha 4	3,49718421	5,70645557	-3,9738833	-15,712962	1
CRMP1	collapsin response mediator protein 1	5,89671109	9,94014719	-3,9747198	-15,722075	1
IL7R	interleukin 7 receptor	8,70413952	11,5232355	-3,9784206	-15,762458	1
RTN1	reticulon 1	3,71535132	6,36646859	-3,9809368	-15,789973	1
CPEB1	cytoplasmic polyadenylation element binding protein 1	4,51897492	8,59146131	-3,9998336	-15,998155	1
KCNMA1	potassium large conductance calcium-activated channel, subfamily M, alpha	5,13859066	9,42379273	-4,0021619	-16,023994	1
BDNF	brain-derived neurotrophic factor	7,17382758	10,5434348	-4,0068571	-16,076229	1
MGLL	monoglyceride lipase	8,26303422	11,3530214	-4,0093786	-16,104351	1
GPNMB	glycoprotein (transmembrane) nmb	5,14761577	9,44711323	-4,0114897	-16,127934	1
FBXO25	F-box protein 25	4,00736032	7,67462921	-4,017736	-16,197912	1
THBS1	thrombospondin 1	6,35318778	10,1215158	-4,019526	-16,218023	1
ZNF571	zinc finger protein 571	3,30362042	4,77625621	-4,0256039	-16,28649	0,99999999
ABLIM3	actin binding LIM protein family, member 3	5,04247586	9,32685498	-4,0298391	-16,334372	1
NHLRC1	NHL repeat containing 1	3,5166249	5,83144384	-4,0307929	-16,345175	1
CTAG2	cancer/testis antigen 2	4,79855367	8,97893376	-4,0312038	-16,349831	1
SMARCA2	SWI/SNF related, matrix associated, actin dependent regulator of chromatin	3,85464818	7,00465335	-4,0485654	-16,547775	1
ALPL	alkaline phosphatase, liver/bone/kidney	4,40324142	8,51227126	-4,0506106	-16,571251	1
CRMP1	collapsin response mediator protein 1	5,74268779	9,94595411	-4,0513879	-16,580181	1
MYEF2	myelin expression factor 2	3,0752944	3,90596874	-4,0537625	-16,607494	0,99224552

EREG	epiregulin	3,74931581	6,58145949	-4,0594188	-16,672735	1
PLCL1	phospholipase C-like 1	3,53014377	5,93083448	-4,0843425	-16,963271	1
KCTD12	potassium channel tetramerisation domain containing 12	3,89689031	7,29701641	-4,0864101	-16,987599	1
TGFBI	transforming growth factor, beta-induced, 68kDa	11,9763962	13,2923588	-4,0894248	-17,023134	0,99999931
CXCR7	chemokine (C-X-C motif) receptor 7	4,55307506	8,73435047	-4,0897737	-17,027251	1
VCAN	versican	3,35610608	5,21008929	-4,0953844	-17,093601	1
ABLIM3	actin binding LIM protein family, member 3	5,7321933	9,98477003	-4,1030227	-17,184342	1
MYH10	myosin, heavy chain 10, non-muscle	4,72912466	8,97262382	-4,1032615	-17,187187	1
FAM171A1	family with sequence similarity 171, member A1	4,9351818	9,27100704	-4,1074134	-17,236721	1
KCNG3	potassium voltage-gated channel, subfamily G, member 3	3,71045891	6,51624999	-4,1120356	-17,292033	1
NRIP3	nuclear receptor interacting protein 3	4,29701943	8,45093729	-4,1130732	-17,304474	1
C11orf63	chromosome 11 open reading frame 63	3,06304601	3,89730712	-4,1196729	-17,383816	0,99264089
LHFP	lipoma HMGIC fusion partner	3,90746368	7,4287328	-4,1214347	-17,405057	1
NID1	nidogen 1	4,53778484	8,75607367	-4,1246062	-17,443362	1
KCTD12	potassium channel tetramerisation domain containing 12	4,14997627	8,24036027	-4,1275006	-17,478393	1
AMIGO2	adhesion molecule with Ig-like domain 2	7,27689302	10,719918	-4,1357858	-17,579058	1
GPNMB	glycoprotein (transmembrane) nmb	5,91069539	10,0810912	-4,1500245	-17,753413	1
FOSL1	FOS-like antigen 1	7,45051428	10,8739087	-4,1522844	-17,781245	1
NUPR1	nuclear protein, transcriptional regulator, 1	5,99273966	10,1132175	-4,1610239	-17,889286	1
DNER	delta/notch-like EGF repeat containing	5,41779897	9,88792257	-4,1699992	-18,000926	1
PID1	phosphotyrosine interaction domain containing 1	6,12359172	10,158151	-4,1713449	-18,017725	1
PSG2	pregnancy specific beta-1-glycoprotein 2	3,00095227	3,76632252	-4,1728841	-18,036958	0,9805704
PRKAR2B	protein kinase, cAMP-dependent, regulatory, type II, beta	3,51058384	5,97322075	-4,1810967	-18,139927	1
ZNF83	zinc finger protein 83	4,02026478	8,01517151	-4,1816452	-18,146825	1
SLC30A4	solute carrier family 30 (zinc transporter), member 4	3,12262693	4,09421493	-4,1998976	-18,377869	0,99914837
THBS1	thrombospondin 1	7,3565548	10,8345281	-4,2017308	-18,401236	1
S1PR1	sphingosine-1-phosphate receptor 1	3,91649749	7,65506394	-4,203932	-18,429333	1
TCEAL3	transcription elongation factor A (SII)-like 3	5,51301326	9,97834124	-4,2063739	-18,460554	1
PAK3	p21 protein (Cdc42/Rac)-activated kinase 3	3,17126599	4,27327425	-4,2202284	-18,638688	0,99993112
GPNMB	glycoprotein (transmembrane) nmb	5,03558246	9,5743791	-4,2324733	-18,797558	1
TGFBI	transforming growth factor, beta-induced, 68kDa	11,9885557	13,3440896	-4,2336278	-18,812605	0,99999974

C21orf7	chromosome 21 open reading frame 7	3,32039359	5,17148405	-4,2345147	-18,824175	1
IGFBP7	insulin-like growth factor binding protein 7	3,45308582	5,85096427	-4,2456424	-18,96993	1
C16orf45	chromosome 16 open reading frame 45	5,50926585	10,029821	-4,2653078	-19,230279	1
FMN1	formin 1	3,23902095	4,63955696	-4,2703244	-19,297264	0,99999992
SMARCA2	SWI/SNF related, matrix associated, actin dependent regulator of chromatir	3,79606696	7,18925816	-4,2915252	-19,582937	1
TIMP3	TIMP metallopeptidase inhibitor 3	6,56762978	10,3974132	-4,2919513	-19,588721	1
AKR1C2	aldo-keto reductase family 1, member C2 (dihydrodiol dehydrogenase 2; bil	6,71594809	10,4677765	-4,2926365	-19,598027	1
ANKRD1	ankyrin repeat domain 1 (cardiac muscle)	3,28969659	5,03683795	-4,2982496	-19,674425	1
STK32B	serine/threonine kinase 32B	3,88125525	7,74013591	-4,3229914	-20,014746	1
HKR1	HKR1, GLI-Kruppel zinc finger family member	4,51598672	8,97628307	-4,3371955	-20,212775	1
KCNH1	potassium voltage-gated channel, subfamily H (eag-related), member 1	3,7090674	6,85345529	-4,3447333	-20,318659	1
TAGLN	transgelin	5,95644939	10,2411281	-4,3467629	-20,347263	1
LBH	limb bud and heart development homolog (mouse)	5,10443636	9,80240536	-4,3482818	-20,368698	1
VCAN	versican	3,15593652	4,28737752	-4,3485521	-20,372514	0,99996179
SEMA5A	sema domain, seven thrombospondin repeats (type 1 and type 1-like), trans	3,29364918	5,15349876	-4,3537271	-20,445722	1
PRUNE2	prune homolog 2 (Drosophila)	3,42976165	5,88891033	-4,3541117	-20,451173	1
ZNF214	zinc finger protein 214	2,99446879	3,80613787	-4,3624078	-20,569115	0,98980262
FBN1	fibrillin 1	3,44454514	5,95952448	-4,3690073	-20,663422	1
WNT5A	wingless-type MMTV integration site family, member 5A	8,76719367	11,7367866	-4,3765084	-20,771138	1
LAMA4	laminin, alpha 4	8,7612445	11,7360897	-4,3785618	-20,800724	1
KCNS3	potassium voltage-gated channel, delayed-rectifier, subfamily S, member 3	3,48696704	6,12727407	-4,3903236	-20,970998	1
THBS1	thrombospondin 1	8,44899789	11,6489369	-4,3939362	-21,023577	1
ITGAV	integrin, alpha V (vitronectin receptor, alpha polypeptide, antigen CD51)	3,27911284	5,10372547	-4,3964044	-21,059575	1
PRUNE2	prune homolog 2 (Drosophila)	3,52025704	6,24313393	-4,3974538	-21,074899	1
ALPL	alkaline phosphatase, liver/bone/kidney	5,36994748	10,0854209	-4,4050869	-21,186699	1
HHIP	hedgehog interacting protein	3,37585632	5,74079604	-4,4141861	-21,320747	1
RPS6KA2	ribosomal protein S6 kinase, 90kDa, polypeptide 2	7,76019753	11,3160974	-4,415595	-21,341579	1
PDE4B	phosphodiesterase 4B, cAMP-specific	3,94650015	8,19382142	-4,4196559	-21,401736	1
ZNF83	zinc finger protein 83	3,94740833	8,22326657	-4,4352961	-21,635014	1
TCEAL6	transcription elongation factor A (SII)-like 6	4,42304652	8,98103376	-4,4388659	-21,688613	1
ETV5	ets variant 5	3,28544205	5,23038271	-4,4421272	-21,737696	1

KCNK2	potassium channel, subfamily K, member 2	3,59834275	6,57237591	-4,453212	-21,90536	1
COL14A1	collagen, type XIV, alpha 1	3,14607739	4,31011176	-4,4541495	-21,9196	0,99998017
CLDN1	claudin 1	3,47588896	6,17527235	-4,4652911	-22,089535	1
DZIP1	DAZ interacting protein 1	3,22253979	4,75670806	-4,475275	-22,24293	1
STXBP6	syntaxin binding protein 6 (amisyn)	3,93345825	8,25686256	-4,4801192	-22,317743	1
AASS	aminoadipate-semialdehyde synthase	3,22128879	4,75800655	-4,4839018	-22,376334	1
RND3	Rho family GTPase 3	10,1660051	12,2374729	-4,4863095	-22,413709	1
NRP1	neuropilin 1	10,087683	12,2103289	-4,5079973	-22,753196	1
CRLF1	cytokine receptor-like factor 1	5,9140683	10,3594337	-4,5223588	-22,980826	1
CPEB1	cytoplasmic polyadenylation element binding protein 1	3,31033369	5,54336563	-4,5252562	-23,027026	1
KRT17	keratin 17	11,4389013	13,0097432	-4,534432	-23,173948	1
TMOD2	tropomodulin 2 (neuronal)	3,0883985	4,13362574	-4,5346473	-23,177408	0,99979146
ALPL	alkaline phosphatase, liver/bone/kidney	4,68559204	9,45330991	-4,5362945	-23,203885	1
ODZ2	odz, odd Oz/ten-m homolog 2 (Drosophila)	4,68702213	9,45902044	-4,539065	-23,248488	1
ALPL	alkaline phosphatase, liver/bone/kidney	5,31646654	10,1830641	-4,5398478	-23,261107	1
IGFBP7	insulin-like growth factor binding protein 7	9,09245769	11,8934368	-4,5403334	-23,268938	1
LBH	limb bud and heart development homolog (mouse)	5,12023985	10,039655	-4,540744	-23,27556	1
CPEB1	cytoplasmic polyadenylation element binding protein 1	3,85510369	8,10195832	-4,5496743	-23,420084	1
MYL9	myosin, light chain 9, regulatory	9,96752176	12,1757481	-4,5527043	-23,469323	1
C16orf45	chromosome 16 open reading frame 45	7,50996502	11,2303019	-4,5555252	-23,515257	1
KCNMA1	potassium large conductance calcium-activated channel, subfamily M, alpha	3,30715445	5,57775518	-4,5617261	-23,616546	1
NID1	nidogen 1	3,33137968	5,72210214	-4,5658053	-23,683417	1
HAS2	hyaluronan synthase 2	3,36165907	5,89000727	-4,5842607	-23,988327	1
IGFBP7	insulin-like growth factor binding protein 7	3,35696252	5,88059281	-4,5932365	-24,138038	1
C21orf7	chromosome 21 open reading frame 7	3,26428689	5,32214516	-4,6000673	-24,252596	1
MYL9	myosin, light chain 9, regulatory	10,0197566	12,2174993	-4,6114547	-24,444782	1
DKK3	dickkopf homolog 3 (Xenopus laevis)	5,61320847	10,362221	-4,6175438	-24,548174	1
KANK1	KN motif and ankyrin repeat domains 1	3,32906931	5,7846748	-4,6210059	-24,607154	1
BEX2	brain expressed X-linked 2	3,23032118	5,05347726	-4,6294471	-24,751551	1
TIMP3	TIMP metallopeptidase inhibitor 3	9,35785257	12,0002922	-4,6327624	-24,808496	1
PTK7	PTK7 protein tyrosine kinase 7	2,7521516	3,37720781	-4,6347187	-24,842159	0,90021508

ODZ2	odz, odd Oz/ten-m homolog 2 (Drosophila)	4,98279929	10,0076105	-4,6378519	-24,896169	1
IL15	interleukin 15	3,42055534	6,18758385	-4,6416505	-24,961807	1
LBH	limb bud and heart development homolog (mouse)	3,88767675	8,41466733	-4,6551559	-25,196577	1
CPEB1	cytoplasmic polyadenylation element binding protein 1	3,76418198	7,86516997	-4,6558714	-25,209076	1
RPS6KA2	ribosomal protein S6 kinase, 90kDa, polypeptide 2	6,31189691	10,5599723	-4,661234	-25,302955	1
NRIP3	nuclear receptor interacting protein 3	3,16393793	4,55832296	-4,6639038	-25,349823	0,9999999
CXorf26	chromosome X open reading frame 26	3,96115392	8,61519871	-4,6666068	-25,397363	1
FLI1	Friend leukemia virus integration 1	3,43891666	6,28963106	-4,6731803	-25,513348	1
RPS6KA2	ribosomal protein S6 kinase, 90kDa, polypeptide 2	7,88957503	11,5757891	-4,6797691	-25,630133	1
ZC4H2	zinc finger, C4H2 domain containing	3,64225321	7,14533425	-4,6893525	-25,800953	1
ZNF285	zinc finger protein 285	3,32024912	5,84221181	-4,6970579	-25,939124	1
RERG	RAS-like, estrogen-regulated, growth inhibitor	4,32760115	9,1897574	-4,717215	-26,304085	1
ZNF85	zinc finger protein 85	3,13320893	4,44603953	-4,7411755	-26,744596	0,99999925
PRKAR2B	protein kinase, cAMP-dependent, regulatory, type II, beta	3,11429171	4,353787	-4,7458031	-26,830521	0,99999578
TM6SF1	transmembrane 6 superfamily member 1	3,20681782	5,05507467	-4,7641421	-27,173756	1
SGIP1	SH3-domain GRB2-like (endophilin) interacting protein 1	2,75846644	3,41377469	-4,7696756	-27,278182	0,92799198
AFP	alpha-fetoprotein	3,51527106	6,69029154	-4,7718175	-27,318712	1
DCLK1	doublecortin-like kinase 1	3,48837896	6,59671228	-4,7788846	-27,452861	1
GREM1	gremlin 1	3,639886	7,32846628	-4,7816881	-27,506261	1
TGFBI	transforming growth factor, beta-induced, 68kDa	11,5233974	13,154286	-4,8145576	-28,14014	1
PDE4B	phosphodiesterase 4B, cAMP-specific	3,67379995	7,71773928	-4,8385954	-28,612932	1
SMARCA2	SWI/SNF related, matrix associated, actin dependent regulator of chromatin	3,58158343	7,13521997	-4,8575585	-28,991508	1
UBASH3B	ubiquitin associated and SH3 domain containing B	3,19854397	5,15349876	-4,8613263	-29,067323	1
SPHK1	sphingosine kinase 1	8,6644018	11,9339235	-4,8633212	-29,107544	1
PHLDA1	pleckstrin homology-like domain, family A, member 1	3,45876551	6,60965971	-4,8741338	-29,326516	1
ARMCX1	armadillo repeat containing, X-linked 1	3,63694626	7,54243896	-4,8774529	-29,394063	1
TNFAIP6	tumor necrosis factor, alpha-induced protein 6	4,61868208	9,86834916	-4,9305207	-30,495421	1
KRCC1	lysine-rich coiled-coil 1	3,13716866	4,64706429	-4,9310731	-30,507099	1
LIMS2	LIM and senescent cell antigen-like domains 2	7,10979004	11,2141054	-4,9527044	-30,967959	1
KCNK2	potassium channel, subfamily K, member 2	3,16925388	4,99814153	-4,9585725	-31,094176	1
NCK2	NCK adaptor protein 2	4,44991579	9,64835197	-4,9599523	-31,123929	1

MAGED1	melanoma antigen family D, 1	3,14264801	4,73259884	-4,9647542	-31,227696	1
DET1	de-etiolated homolog 1 (Arabidopsis)	3,26281173	5,9254375	-4,9764459	-31,481795	1
FMN1	formin 1	3,03154911	4,14079542	-4,9803924	-31,568031	0,99994037
THBS1	thrombospondin 1	3,60250791	7,5813444	-4,9915502	-31,813124	1
DKK3	dickkopf homolog 3 (Xenopus laevis)	6,1925238	10,7569841	-4,9973686	-31,941687	1
CRB1	crumbs homolog 1 (Drosophila)	3,31526789	6,22868399	-5,0122642	-32,273188	1
GLIPR1	GLI pathogenesis-related 1	9,29649275	12,1332077	-5,0233401	-32,521909	1
ZC4H2	zinc finger, C4H2 domain containing	3,5940588	7,61387549	-5,0282292	-32,632309	1
GBP1	guanylate binding protein 1, interferon-inducible	3,21449574	5,69706726	-5,0408097	-32,918111	1
HAS2	hyaluronan synthase 2	3,34297766	6,38881753	-5,049036	-33,106348	1
SLCO1B3	solute carrier organic anion transporter family, member 1B3	3,23057946	5,84009421	-5,0523238	-33,181881	1
CPA3	carboxypeptidase A3 (mast cell)	3,76937089	8,73369009	-5,0544523	-33,230874	1
PLSCR4	phospholipid scramblase 4	3,4647329	6,91201003	-5,0696693	-33,583234	1
CRMP1	collapsin response mediator protein 1	6,09283767	10,7942919	-5,0880328	-34,013436	1
SH3GL3	SH3-domain GRB2-like 3	3,49268603	7,10138454	-5,1003277	-34,304541	1
KCNK2	potassium channel, subfamily K, member 2	3,14215654	4,97991592	-5,1195533	-34,76475	1
SMARCA2	SWI/SNF related, matrix associated, actin dependent regulator of chromatir	3,28828443	6,32569722	-5,1781442	-36,205683	1
C5orf25	chromosome 5 open reading frame 25	3,39351153	6,76754104	-5,1834761	-36,339738	1
PID1	phosphotyrosine interaction domain containing 1	3,24219575	6,11152884	-5,1841698	-36,357216	1
ST3GAL6	ST3 beta-galactoside alpha-2,3-sialyltransferase 6	3,1107347	4,74584989	-5,191687	-36,547151	1
FLI1	Friend leukemia virus integration 1	3,10120489	4,68639148	-5,2134192	-37,101849	1
KCNMA1	potassium large conductance calcium-activated channel, subfamily M, alpha	3,62912381	8,60227826	-5,2790037	-38,827414	1
C3orf67	chromosome 3 open reading frame 67	3,55481925	8,04010572	-5,2836967	-38,953923	1
CRMP1	collapsin response mediator protein 1	7,06327307	11,4332677	-5,2864877	-39,029356	1
TMEM156	transmembrane protein 156	3,18213011	5,89374883	-5,2932868	-39,213727	1
FAP	fibroblast activation protein, alpha	3,78180812	9,23333235	-5,3617197	-41,118612	1
SMARCA2	SWI/SNF related, matrix associated, actin dependent regulator of chromatir	4,03481757	9,61687768	-5,3822215	-41,707113	1
KCTD12	potassium channel tetramerisation domain containing 12	3,48428587	7,6788181	-5,3832897	-41,738004	1
CXorf26	chromosome X open reading frame 26	3,99727503	9,58146993	-5,3911716	-41,966656	1
CHSY3	chondroitin sulfate synthase 3	3,24299347	6,44181354	-5,4193595	-42,794678	1
AMOT	angiomotin	3,28034467	6,65612679	-5,4536561	-43,824209	1

GREM1	gremlin 1	3,11852825	5,61538722	-5,4855475	-44,803747	1
KHDRBS3	KH domain containing, RNA binding, signal transduction associated 3	3,12507775	5,72241824	-5,4882065	-44,886402	1
TUB	tubby homolog (mouse)	3,6614543	9,14918687	-5,4914361	-44,986995	1
IL1R1	interleukin 1 receptor, type I	3,31846492	6,90622917	-5,5101941	-45,575737	1
RSPO3	R-spondin 3 homolog (Xenopus laevis)	3,84047442	9,56726532	-5,5464195	-46,734611	1
KCTD12	potassium channel tetramerisation domain containing 12	3,46874157	8,17305575	-5,5807536	-47,86017	1
ADRA2A	adrenergic, alpha-2A-, receptor	3,1672458	6,29713059	-5,5957782	-48,361204	1
S1PR1	sphingosine-1-phosphate receptor 1	3,11636678	5,88445929	-5,5961067	-48,372214	1
SLC43A3	solute carrier family 43, member 3	4,36531533	10,3706954	-5,6036553	-48,625976	1
FAM171A1	family with sequence similarity 171, member A1	3,56702762	9,02066521	-5,6049921	-48,671053	1
UCHL1	ubiquitin carboxyl-terminal esterase L1 (ubiquitin thiolesterase)	3,42665727	7,86297297	-5,6265965	-49,40539	1
TAGLN	transgelin	6,5006212	11,363066	-5,6949741	-51,80337	1
FBXO25	F-box protein 25	3,39502692	7,78801452	-5,7054836	-52,182117	1
LST-3TM12	organic anion transporter LST-3b	3,07888811	5,76522528	-5,7483196	-53,754723	1
GPNMB	glycoprotein (transmembrane) nmb	3,29635061	7,21900009	-5,7749706	-54,756967	1
BCAT1	branched chain amino-acid transaminase 1, cytosolic	4,07959832	10,1545714	-5,7755153	-54,777644	1
CTHRC1	collagen triple helix repeat containing 1	3,310907	7,43821918	-5,8421407	-57,366663	1
SEMA5A	sema domain, seven thrombospondin repeats (type 1 and type 1-like), trans	3,20681782	6,87667953	-5,8508366	-57,713486	1
AKR1C3	aldo-keto reductase family 1, member C3 (3-alpha hydroxysteroid dehydrog	9,46004573	12,4662388	-5,8533186	-57,812864	1
MAMDC2	MAM domain containing 2	3,04579148	5,36064512	-5,8645403	-58,264301	1
GREM1	gremlin 1	3,24162806	7,2024285	-5,9437139	-61,55115	1
BCAT1	branched chain amino-acid transaminase 1, cytosolic	4,19748661	10,5689695	-5,9511186	-61,867877	1
KCTD12	potassium channel tetramerisation domain containing 12	3,25491862	7,33151555	-5,9724507	-62,789471	1
ZNF711	zinc finger protein 711	3,04579148	5,95649961	-5,983146	-63,256683	1
TAGLN	transgelin	6,89567998	11,8392238	-5,9853722	-63,354368	1
TMSB15A	thymosin beta 15a	3,68492767	9,90382872	-5,9899215	-63,554463	1
EREG	epiregulin	3,07880736	6,37672047	-6,0041182	-64,18295	1
IGFBP4	insulin-like growth factor binding protein 4	8,08121226	12,3662951	-6,0178821	-64,798211	1
TNFRSF11B	tumor necrosis factor receptor superfamily, member 11b	3,85573101	10,2247459	-6,0699792	-67,180901	1
NRIP3	nuclear receptor interacting protein 3	3,3083476	8,03957164	-6,0725975	-67,302934	1
NTN4	netrin 4	3,17424997	7,16932593	-6,1488698	-70,956836	1

TAGLN	transgelin	6,84046854	11,9320883	-6,1609536	-71,553655	1
TAGLN	transgelin	6,28818061	11,5962627	-6,1628757	-71,649052	1
DCLK1	doublecortin-like kinase 1	3,00150978	5,57101289	-6,1804006	-72,524704	1
UCHL1	ubiquitin carboxyl-terminal esterase L1 (ubiquitin thiolesterase)	3,24299347	7,70514408	-6,1875204	-72,883505	1
PLA2R1	phospholipase A2 receptor 1, 180kDa	2,96786865	4,66002723	-6,2089416	-73,973757	1
RNF182	ring finger protein 182	3,75358504	10,2678245	-6,2096465	-74,009909	1
TAGLN	transgelin	6,41555504	11,7065622	-6,2312058	-75,124197	1
BCAT1	branched chain amino-acid transaminase 1, cytosolic	3,45450972	9,83773815	-6,2631608	-76,80673	1
BCAT1	branched chain amino-acid transaminase 1, cytosolic	3,51710242	10,0002147	-6,2725878	-77,310249	1
SLC43A3	solute carrier family 43, member 3	3,86475072	10,5741656	-6,3404935	-81,036137	1
IGFBP4	insulin-like growth factor binding protein 4	5,59676311	11,7202335	-6,5507479	-93,750072	1
MAGEA11	melanoma antigen family A, 11	3,29697301	9,79093541	-6,5592086	-94,301484	1
GPNMB	glycoprotein (transmembrane) nmb	3,47927922	10,4129196	-6,6205481	-98,397387	1
BCAT1	branched chain amino-acid transaminase 1, cytosolic	3,29284772	9,95319312	-6,6400431	-99,736045	1
NPNT	nephronectin	2,93023369	5,54322221	-6,6788185	-102,45301	1
PRUNE2	prune homolog 2 (Drosophila)	2,95150197	6,64757221	-6,7036513	-104,23177	1
MANSC1	MANSC domain containing 1	2,90788751	4,91634127	-6,8432395	-114,82075	1
ACTC1	actin, alpha, cardiac muscle 1	3,98580621	11,5053998	-6,8700676	-116,9759	1
SLC43A3	solute carrier family 43, member 3	3,74079422	11,1232398	-6,8980326	-119,26547	1
AKR1C2	aldo-keto reductase family 1, member C2 (dihydrodiol dehydrogenase 2; bil	7,80952415	12,8282609	-7,0661232	-134,00316	1
ARMCX1	armadillo repeat containing, X-linked 1	2,96780564	7,49127437	-7,0750013	-134,83033	1
RERG	RAS-like, estrogen-regulated, growth inhibitor	2,99491112	7,9359705	-7,1837819	-145,38977	1
SLC43A3	solute carrier family 43, member 3	3,34696342	11,8206841	-7,8435267	-229,68721	1
IGFBP4	insulin-like growth factor binding protein 4	7,88149374	13,1551849	-7,8682829	-233,66258	1
SPINK6	serine peptidase inhibitor, Kazal type 6	5,74233493	13,0486061	-8,4937581	-360,47566	1
IGFBP4	insulin-like growth factor binding protein 4	8,57731452	13,4267832	-8,9731991	-502,57641	1
EREG	epiregulin	2,68258214	5,0009299	-9,0409185	-526,72951	1
